# Comparison for the effects of different components of temperature variability on mortality: A multi-country time-series study

**DOI:** 10.1016/j.envint.2024.108712

**Published:** 2024-05-03

**Authors:** Bo Wen, Yao Wu, Yuming Guo, Antonio Gasparrini, Shilu Tong, Ala Overcenco, Aleš Urban, Alexandra Schneider, Alireza Entezari, Ana Maria Vicedo-Cabrera, Antonella Zanobetti, Antonis Analitis, Ariana Zeka, Aurelio Tobias, Baltazar Nunes, Barrak Alahmad, Ben Armstrong, Bertil Forsberg, Shih-Chun Pan, Carmen Íñiguez, Caroline Ameling, César De la Cruz Valencia, Christofer Åström, Danny Houthuijs, Do Van Dung, Dominic Royé, Ene Indermitte, Eric Lavigne, Fatemeh Mayvaneh, Fiorella Acquaotta, Francesca de’Donato, Shilpa Rao, Francesco Sera, Gabriel Carrasco-Escobar, Haidong Kan, Hans Orru, Ho Kim, Iulian-Horia Holobaca, Jan Kyselý, Joana Madureira, Joel Schwartz, Jouni J.K. Jaakkola, Klea Katsouyanni, Magali Hurtado Diaz, Martina S. Ragettli, Masahiro Hashizume, Mathilde Pascal, Micheline de Sousa Zanotti Stagliorio Coélho, Nicolás Valdés Ortega, Niilo Ryti, Noah Scovronick, Paola Michelozzi, Patricia Matus Correa, Patrick Goodman, Paulo Hilario Nascimento Saldiva, Raanan Raz, Rosana Abrutzky, Samuel Osorio, Tran Ngoc Dang, Valentina Colistro, Veronika Huber, Whanhee Lee, Xerxes Seposo, Yasushi Honda, Yoonhee Kim, Yue Leon Guo, Michelle L. Bell, Shanshan Li

**Affiliations:** a Climate, Air Quality Research Unit, School of Public Health and Preventive Medicine, Monash University, Melbourne, Australia; b Department of Public Health, Environments and Society, London School of Hygiene & Tropical Medicine, London, UK; c Centre for Statistical Methodology, London School of Hygiene & Tropical Medicine, London, UK; d Centre on Climate Change & Planetary Health, London School of Hygiene & Tropical Medicine, London, UK; e Shanghai Children’s Medical Centre, Shanghai Jiao Tong University, Shanghai, China; f School of Public Health, Institute of Environment and Population Health, Anhui Medical University, Hefei, China; g Center for Global Health, Nanjing Medical University, Nanjing, China; h School of Public Health and Social Work, Queensland University of Technology, Brisbane, Australia; i National Agency for Public Health of the Ministry of Health, Labour and Social Protection of the Republic of Moldova, Republic of Moldova; j Institute of Atmospheric Physics, Czech Academy of Sciences, Prague, Czech Republic; k Faculty of Environmental Sciences, Czech University of Life Sciences, Prague, Czech Republic; l Institute of Epidemiology, Helmholtz Zentrum München – German Research Center for Environmental Health, Neuherberg, Germany; m Faculty of Geography and Environmental Sciences, Hakim Sabzevari University, Sabzevar, Iran; n Institute of Social and Preventive Medicine, University of Bern, Bern, Switzerland; o Oeschger Center for Climate Change Research, University of Bern, Bern, Switzerland; p Department of Environmental Health, Harvard T.H. Chan School of Public Health, Harvard University, Boston, MA, USA; q Department of Hygiene, Epidemiology and Medical Statistics, National and Kapodistrian University of Athens, Athens, Greece; r Institute for Environment, Health and Societies, Brunel University London, London, UK; s Institute of Environmental Assessment and Water Research, Spanish Council for Scientific Research, Barcelona, Spain; t School of Tropical Medicine and Global Health, Nagasaki University, Nagasaki, Japan; u Department of Epidemiology, Instituto Nacional de Saúde Dr Ricardo Jorge, Porto, Portugal; v Centro de Investigação em Saúde Pública, Escola Nacional de Saúde Pública, Universidade NOVA de Lisboa, Lisbon, Portugal; w Department of Public Health and Clinical Medicine, Umeå University, Umeå, Sweden; x National Institute of Environmental Health Science, National Health Research Institutes, Zhunan, Taiwan; y Department of Statistics and Computational Research, Universitat de València, València, Spain; z CIBER of Epidemiology and Public Health, Madrid, Spain; aa National Institute for Public Health and the Environment (RIVM), Centre for Sustainability and Environmental Health, Bilthoven, Netherlands; ab Department of Environmental Health, National Institute of Public Health, Cuernavaca Morelos, Mexico; ac Department of Environmental Health, Faculty of Public Health, University of Medicine and Pharmacy at Ho Chi Minh City, Ho Chi Minh City, Viet Nam; ad Department of Geography, University of Santiago de Compostela, Santiago de Compostela, Spain; ae Institute of Family Medicine and Public Health, University of Tartu, Tartu, Estonia; af School of Epidemiology & Public Health, Faculty of Medicine, University of Ottawa, Ottawa, ON, Canada; ag Air Health Science Division, Health Canada, Ottawa, ON, Canada; ah Department of Earth Sciences, University of Torino, Turin, Italy; ai Department of Epidemiology, Lazio Regional Health Service, Rome, Italy; aj Norwegian Institute of Public Health, Oslo, Norway; ak Department of Statistics, Computer Science and Applications “G. Parenti”, University of Florence, Florence, Italy; al Health Innovation Lab, Institute of Tropical Medicine “Alexander von Humboldt”, Universidad Peruana Cayetano Heredia, Lima, Peru; am Scripps Institution of Oceanography, University of California San Diego, La Jolla, CA, USA; an Department of Environmental Health, School of Public Health, Fudan University, Shanghai, China; ao Graduate School of Public Health, Seoul National University, Seoul, South Korea; ap Faculty of Geography, Babeş-Bolyai University, Cluj-Napoca, Romania; aq Environmental Health Department, Instituto Nacional de Saúde Dr Ricardo Jorge, Porto, Portugal; ar EPIUnit – Instituto de Saúde Pública, Universidade do Porto, Porto, Portugal; as Laboratório para a Investigação Integrativa e Translacional em Saúde Populacional (ITR), Porto, Portugal; at Center for Environmental and Respiratory Health Research (CERH), University of Oulu, Oulu, Finland; au Medical Research Center Oulu (MRC Oulu), Oulu University Hospital and University of Oulu, Oulu, Finland; av School of Population Health and Environmental Sciences, King’s College London, London, UK; aw Swiss Tropical and Public Health Institute, Basel, Switzerland; ax University of Basel, Basel, Switzerland; ay Department of Global Health Policy, Graduate School of Medicine, The University of Tokyo, Tokyo, Japan; az Santé Publique France, Department of Environmental and Occupational Health, French National Public Health Agency, Saint Maurice, France; ba Department of Pathology, Faculty of Medicine, University of São Paulo, Brazil; bb Department of Public Health, Universidad de los Andes, Santiago, Chile; bc Gangarosa Department of Environmental Health, Rollins School of Public Health, Emory University, Atlanta, GA, USA; bd School of Physics, Technological University Dublin, Dublin, Ireland; be INSPER, São Paulo, Brazil; bf Braun School of Public Health and Community Medicine, The Hebrew University of Jerusalem, Israel; bg Universidad de Buenos Aires, Facultad de Ciencias Sociales, Instituto de Investigaciones Gino Germani, Buenos Aires, Argentina; bh Department of Environmental Health, University of São Paulo, São Paulo, Brazil; bi Department of Quantitative Methods, School of Medicine, University of the Republic, Montevideo, Uruguay; bj IBE-Chair of Epidemiology, LMU Munich, Munich, Germany; bk Department of Physical, Chemical and Natural Systems, Universidad Pablo de Olavide, Sevilla, Spain; bl School of the Environment, Yale University, New Haven, CT, USA; bm Department of Occupational and Environmental Medicine, School of Medicine, Ewha Womans University, Seoul, South Korea; bn Center for Climate Change Adaptation, National Institute for Environmental Studies, Tsukuba, Japan; bo Department of Global Environmental Health, Graduate School of Medicine, University of Tokyo, Tokyo, Japan; bp Environmental and Occupational Medicine, National Taiwan University College of Medicine and NTU Hospital, National Taiwan University, Taipei, Taiwan; bq Graduate Institute of Environmental and Occupational Health Sciences, National Taiwan University College of Public Health, National Taiwan University, Taipei, Taiwan

**Keywords:** Temperature variability, Mortality, Inter-day, Intra-day

## Abstract

**Background:**

Temperature variability (TV) is associated with increased mortality risk. However, it is still unknown whether intra-day or inter-day TV has different effects.

**Objectives:**

We aimed to assess the association of intra-day TV and inter-day TV with all-cause, cardiovascular, and respiratory mortality.

**Methods:**

We collected data on total, cardiovascular, and respiratory mortality and meteorology from 758 locations in 47 countries or regions from 1972 to 2020. We defined inter-day TV as the standard deviation (SD) of daily mean temperatures across the lag interval, and intra-day TV as the average SD of minimum and maximum temperatures on each day. In the first stage, inter-day and intra-day TVs were modelled simultaneously in the quasi-Poisson time-series model for each location. In the second stage, a multi-level analysis was used to pool the location-specific estimates.

**Results:**

Overall, the mortality risk due to each interquartile range [IQR] increase was higher for intra-day TV than for inter-day TV. The risk increased by 0.59% (95% confidence interval [CI]: 0.53, 0.65) for all-cause mortality, 0.64% (95% CI: 0.56, 0.73) for cardiovascular mortality, and 0.65% (95% CI: 0.49, 0.80) for respiratory mortality per IQR increase in intra-day TV_0–7_ (0.9 °C). An IQR increase in inter-day TV_0–7_ (1.6 °C) was associated with 0.22% (95% CI: 0.18, 0.26) increase in all-cause mortality, 0.44% (95% CI: 0.37, 0.50) increase in cardiovascular mortality, and 0.31% (95% CI: 0.21, 0.41) increase in respiratory mortality. The proportion of all-cause deaths attributable to intra-day TV_0–7_ and inter-day TV_0–7_ was 1.45% and 0.35%, respectively. The mortality risks varied by lag interval, climate area, season, and climate type.

**Conclusions:**

Our results indicated that intra-day TV may explain the main part of the mortality risk related to TV and suggested that comprehensive evaluations should be proposed in more countries to help protect human health.

## 1. Introduction

Interest in understanding the health impacts of short-term temperature variations has grown in recent decades, as human-induced climate change is increasing the unstable weather conditions in both frequency and intensity across the globe (Guoet al., 2021; IPCC, 2012; Stott, 2016). Studies have reported that increased mortality and morbidity risk was associated with temperature variability (TV), which is defined as the standard deviation of daily maximum and minimum temperatures during several days (Guoet al., 2016; Zhaoet al., 2018; Zhaoet al., 2019). Another two indices of temperature variation frequently used in previous studies were diurnal temperature range (DTR, defined as the difference between daily maximum and minimum temperatures) and temperature change between the neighbouring days (TCN, defined as the difference between daily mean temperatures of two neighbouring days) (Chenget al., 2014; Leeet al., 2018; Linet al., 2013; Maet al., 2020).

Some studies have reported potential differentiated effects of temperature variation within the same day or between neighbouring days by using DTR and TCN as indicators for intra-day and inter-day temperature variation (Hu, 2021; Vicedo-Cabreraet al., 2016). However, there are some limitations. First, only absolute changes in temperature were considered in definitions of DTR or TCN and therefore the effect estimates of the two indicators were not comparable. Second, DTR can only capture the temperature change within one single day. Similarly, TCN can only capture the temperature change within two neighbouring days. These two indicators, however, are unable to measure the temperature variation during a longer period (≥3 days). This may hinder the comprehensive evaluation of health impacts associated with temperature variation. Compared with DTR and TCN, TV has been identified as a better metric to represent temperature variation, as temperature variation is a continuous process and evaluating its impact within a short exposure window (e.g., from two days to seven days) could provide a more comprehensive view (Guoet al., 2016). It is therefore helpful to apply the same framework to define the inter-day and intra-day TV and to further compare their impacts.

In this study, we applied two novel indicators proposed by us recently, intra-day TV and inter-day TV (Wenet al., 2023), to reveal the mortality risk associated with different components of TV. A global analysis within the Multi-Country Multi-City (MCC) Collaborative Research Network was performed to assess the association of intra-day TV and inter-day TV with all-cause, cardiovascular, and respiratory mortality in 758 locations of 47 countries or regions. Following the most comprehensive and standardized analytical framework, we aimed to investigate the health impacts of intra-day and inter-day TVs at the global, regional, and country-level and to further identify potentially vulnerable or susceptible areas.

## 2. Methods

### 2.1. Data collection

Mortality data and meteorological data were obtained from the MCC Collaborative Research Network (https://mccstudy.lshtm.ac.uk/), which has been described in detail in previous studies (Gasparriniet al., 2015; Liuet al., 2019). A total of 758 locations from 47 countries or regions were included in the present study. Generally, we obtained daily counts of mortality data from local authorities of each country or region. We classified causes of death using the international classification of diseases, 9th and 10th revision (ICD-9 and ICD-10) codes, where available. In each location, mortality data for non-external causes (ICD-9: 0–799; ICD-10: A0-R99) were collected while all-cause mortality data were alternatively collected if data on non-external causes were unavailable. We also collected daily counts of mortality specifically for cardiovascular (ICD-10: I00-I99) and respiratory causes (ICD-10: J00-J99). Daily weather data including daily minimum, mean, and maximum temperatures and relative humidity (RH) were collected from local meteorological bureaus or other statistical authorities. Detailed descriptions of data and missing values were provided in the supplementary eMethods.

### 2.2. Definition of inter-day and intra-day temperature variability

In this study, we applied a new definition for inter-day and intra-day TV, computing these indices as the standard deviation (SD) of daily minimum, mean, and maximum temperatures within the lag intervals (Guoet al., 2016; Xuet al., 2020). Details are provided in our previous study(Wenet al., 2023). Briefly, we computed inter-day TV as the SD of daily mean temperature during 0–*L* lag days, and intra-day TV as the average SD of minimum temperature and maximum temperature on each day, which was calculated as follows:

$$TV_{\text{intra}-day,0-L}=\sqrt{\frac{\sum VAR_{l}}{2L+1}}$$

$$TV_{\text{inter}-day,0-L}=\sqrt{\frac{2L\times VAR_{\text{tmean}}}{2L+1}}$$

where *L* is the number of preceding days defining the lag interval (e.g., *L* = 1 when calculating TV_0–1_, *L* = 2 when calculating TV_0–2_, and so on), *VAR*_*l*_ is the variance of daily temperature deviations on lag day *l*, *VAR*_*tmean*_ is the variance of daily mean temperatures within the lag interval. The inter-day TV and intra-day TV could be regarded as components of TV defined in our previous studies because they have the following relationship:

$$TV_{0-L}=\sqrt{T{V_{\text{inter}-day,0-L}}^{2}+T{V_{\text{intra}-day,0-L}}^{2}}$$

Consistent with the previous definition, both inter-day and intra-day TV could account for the lag effects (Guoet al., 2016; Xuet al., 2020). Following these studies, we used inter-day and intra-day TV_0–1_ to TV_0–7_ as the temperature variability exposure.

### 2.3. Statistical analyses

We assessed the associations of mortality with inter-day TV and intra-day TV using a two-stage analytical framework, which has been widely applied in previous multi-center time-series studies (Bellet al., 2005; Gasparriniet al., 2012; Liuet al., 2019). Briefly, a same time-series model was applied for each location and location-specific estimates were then pooled through meta-analyses.

#### 2.3.1. Main analyses

In the first stage, we used a generalized linear regression model with quasi-Poisson family in each location to obtain location-specific estimates. Location-specific daily all-cause or cause-specific mortality counts were treated as the dependent variable in separate models. We simultaneously modelled inter-day TV and intra-day TV using linear functions according to previous studies (Guoet al., 2016; Wenet al., 2023; Xuet al., 2020). In each model, we adjusted for daily mean temperature using a distributed lag non-linear model (DLNM) (Gasparriniet al., 2010). A natural cubic spline with four degrees of freedom was applied for both exposure–response dimension and lag dimension up to 21 days (equally-spaced knots in the log scale of lag days) in the cross-basis function of daily mean temperature (Guoet al., 2016). We also controlled long-term trend and seasonal variations in the model by adding a natural cubic spline of time with seven degrees of freedom (df) per year, and controlled the effects of day of the week by adding an indicator variable for day of the week.

In the second stage, a random-effects multilevel meta-analytical method was applied to pool the location-specific estimations under a hierarchical structure (locations and countries/regions) (Seraet al., 2019). Best linear unbiased predictions (BLUP) were used to obtain location-specific and country-specific associations between mortality with inter-day TV and intra-day TV, which can borrow strength across units within the same level and provide more precise estimations, especially for locations with small data size (Chenet al., 2021; Vicedo-Cabreraet al., 2020). We also examined the heterogeneity across locations using the Cochran Q test and I^2^ statistic. We used fixed effect meta-regressions to test the statistical differences in effect estimations for inter-day TV and intra-day TV (Xuet al., 2019).

The associations of mortality with inter-day TV and intra-day TV were reported as percentage change associated with per interquartile range (IQR) increase of location-specific inter-day TV or intra-day TV and 95 % confidence intervals (95 % CI). We further computed the fractions of deaths attributable to inter-day TV and intra-day TV (above the minimum TV). First, the location-specific number of deaths attributable to inter-day TV or intra-day TV was calculated using pooled effect estimates of 758 locations. Then, the attributable number of deaths in each location was summed to the country-level and the global level, and were then divided by the total number of deaths at each level to derive the attributable fractions (AF) with 95 % CIs. The calculation procedures are shown in detail in the supplementary eMethods.

#### 2.3.2. Stratified analyses

First, we divided 758 locations into four climate groups (cold, moderate cold, moderate warm, and warm areas) by the quantiles of annual mean temperatures (≤25th [10.6 °C], 25th–50th [13.9 °C], 50th–75th [18.8 °C], and ≥75th) during the study period. We pooled the location-specific estimates using a random-effect multilevel meta-analysis to obtain associations in four groups. Second, we examined the associations of mortality with inter-day TV or intra-day TV in different seasons. Stratified analyses were conducted for cold season (4 coldest months), warm season (4 hottest months), and moderate season (other months). The seasons were defined using monthly mean temperatures in each location. Besides, we performed the stratified analyses in Köppen-Geiger climate groups. The Köppen-Geiger climate system divides the locations into five main groups: tropical, dry, temperate, continental, and polar.

#### 2.3.3. Sensitivity analyses

Several sensitivity analyses were conducted to assess the robustness of our results. First, associations of mortality with inter-day TV or intra-day TV were expressed as the relative risk associated with per 1 °C increase in inter-day or intra-day TV. Secondly, lag days of daily mean temperature in our models were changed from 21 to 28. Thirdly, we also changed the degrees of freedom for daily mean temperature (3, 4, 5, and 6 df). Fourth, we used daily minimum temperature or daily maximum temperature to replace the daily mean temperature in our models using the same cross-basis function. Fifth, we adjusted for RH in our models to assess whether RH could confound the associations using the same DLNM model as daily mean temperature. In addition, we performed separate models by including only one of TV indicators (inter-day TV and intra-day TV) to check whether the associations were confounded by themselves. Finally, we further examined the temporal trends in mortality risk linked to inter-day and intra-day TV by dividing the study period into distinct groups: 1972–1980, 1981–1990, 1991–2000, 2001–2010, and 2011–2020. All the analyses were performed by R software (V4.0.3). The “dlnm” package was used to perform the distributed lag linear models and the ‘mixmeta’ package was used to perform the meta-regression models.

## 3. Results

### 3.1. Descriptive statistics

**Fig. 1.**
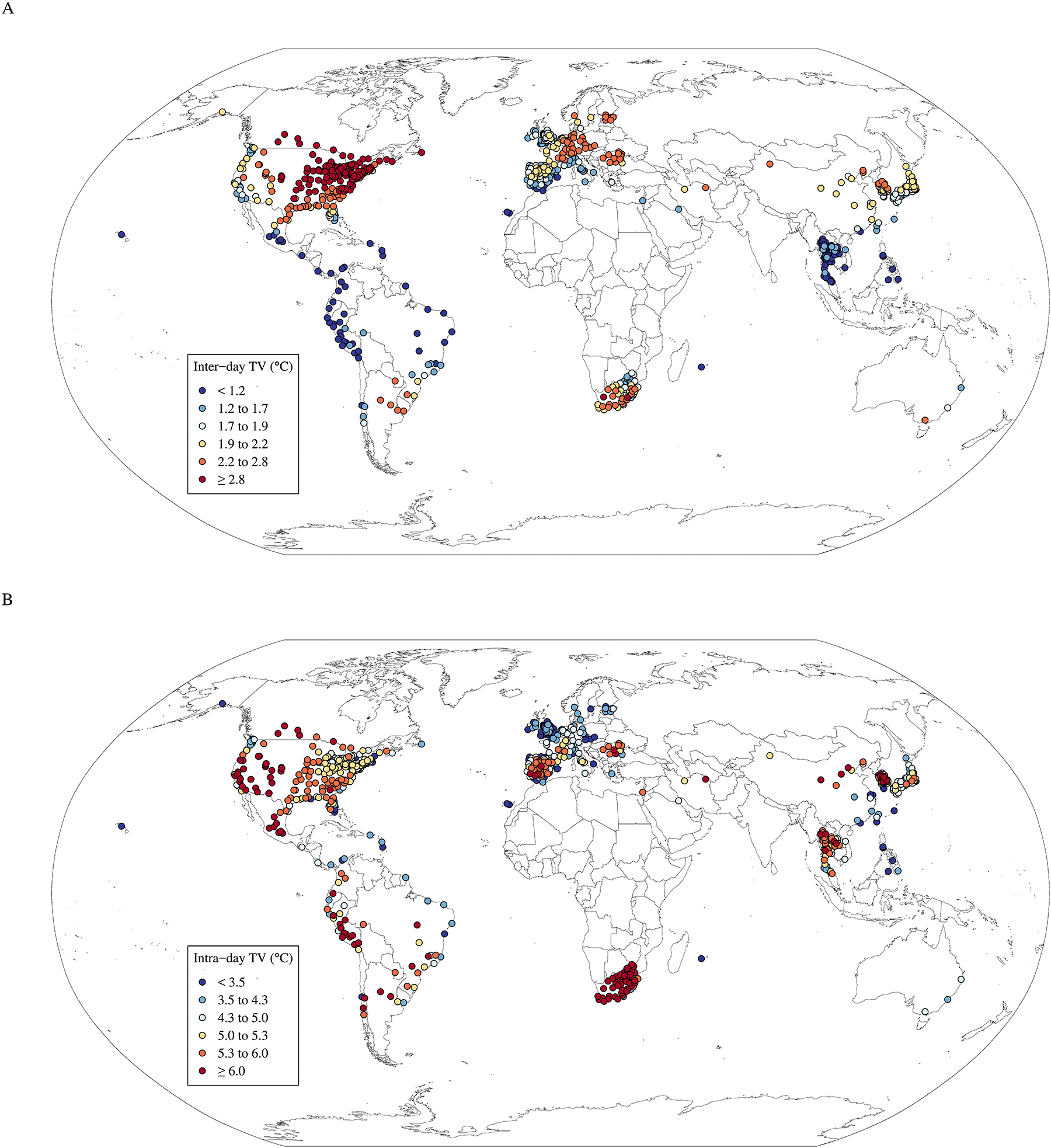
Location of study areas and mean values of inter-day and intra-day temperature variability (°C, TV_0–7_).

This study included a total of 126.6 million deaths in 758 locations of 47 countries or regions covering an average period from 1972 to 2020, including 37.9 million cardiovascular deaths and 13.0 million respiratory deaths (Table 1). Overall, the median inter-day TV_0–7_ was 2.0 °C (interquartile range [IQR]: 1.7 °C, 2.6 °C), which was lower than the median intra-day TV_0–7_ (4.9 °C; IQR: 3.9 °C, 5.5 °C). The summary of inter-day and intra-day TV0–1 to TV0–7 and the proportion of zero death counts are presented in supplementary Table S1 and S2. Fig. 1 shows the average inter-day TV_0–7_ and the average intra-day TV_0–7_ in each location. The highest inter-day TV_0–7_ were mainly observed for locations in the USA and Canada while the lowest levels were mainly observed in Southeast Asia and South America. By contrast, locations with the highest intra-day TV_0–7_ were obtained mainly in South Africa and the USA while the lowest values were mainly observed in Western Europe.

### 3.2. TV-mortality associations

**Fig. 2.**
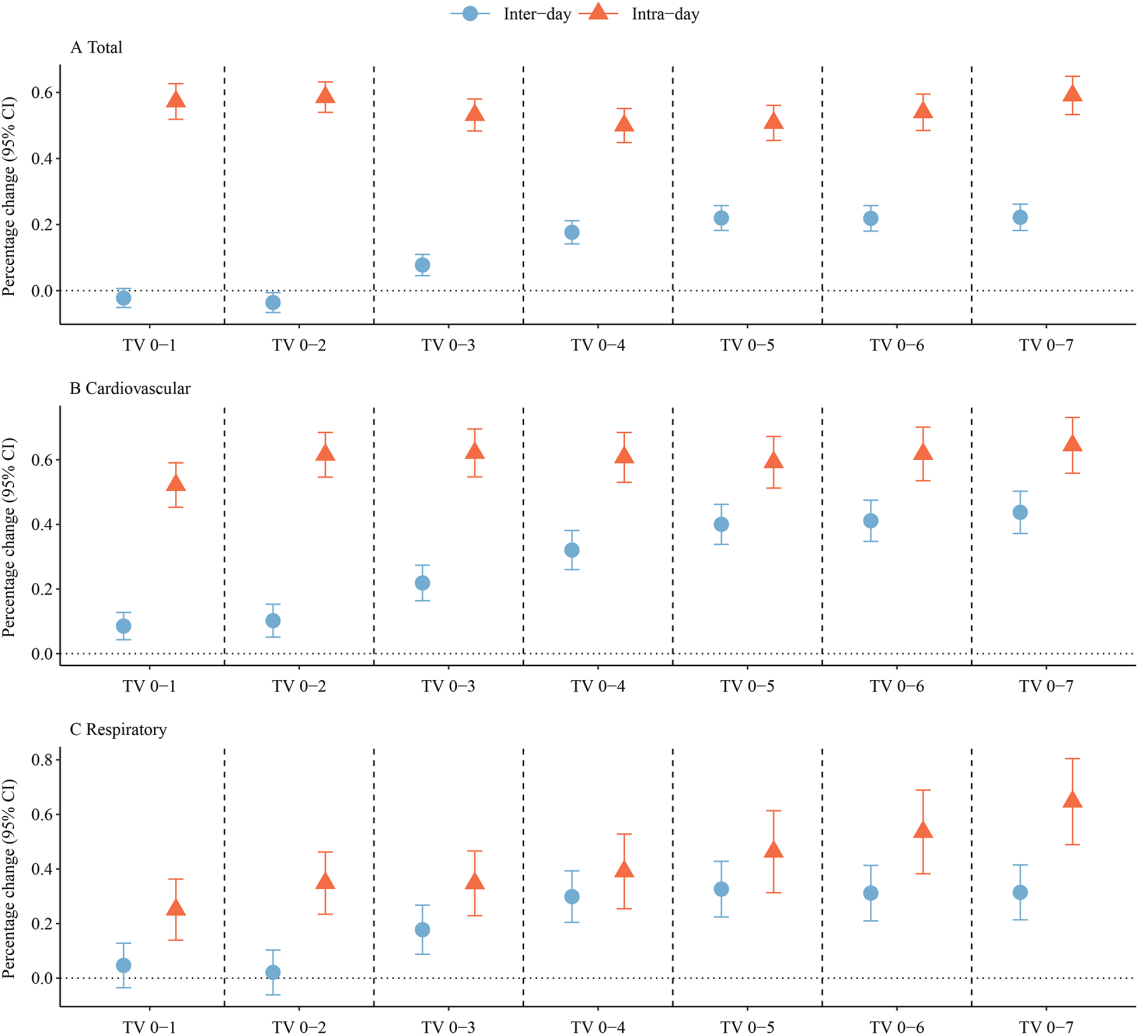
Overall percentage change (%) of mortality risk over one interquartile range of inter-day and intra-day TV (TV_0–1_ to TV_0–7_).

The pooled results of short-term TV-mortality associations across study locations are shown in Fig. 2. The results for test of heterogeneity across locations are shown in supplementary Table S3 and the exact values for Fig. 2 are shown in supplementary Table S4. On average, each IQR increase in intra-day TV_0–7_ was associated with a 0.59 % increase in all-cause mortality (95 % CI: 0.53 %, 0.65 %), 0.64 % increase in cardiovascular mortality (95 % CI: 0.56 %, 0.73 %), and 0.65 % increase in respiratory mortality (95 % CI: 0.49 %, 0.80 %). The mortality risks associated with inter-day TV_0–7_ were lower than the intra-day TV_0–7_. An IQR increase in exposure to inter-day TV_0–7_ elevated the all-cause mortality risk by 0.22 % (95 % CI: 0.18 %, 0.26 %), cardiovascular mortality risk by 0.44 % (95 % CI: 0.37 %, 0.50 %), and respiratory mortality risk by 0.31 % (95 % CI: 0.21 %, 0.41 %). The mortality risks for intra-day and inter-day TV_0–7_ were the highest among all the lag intervals. The exact values of mortality risk for each country or region were shown in supplementary Table S5–S7.

### 3.3. Stratified results

**Fig. 3.**
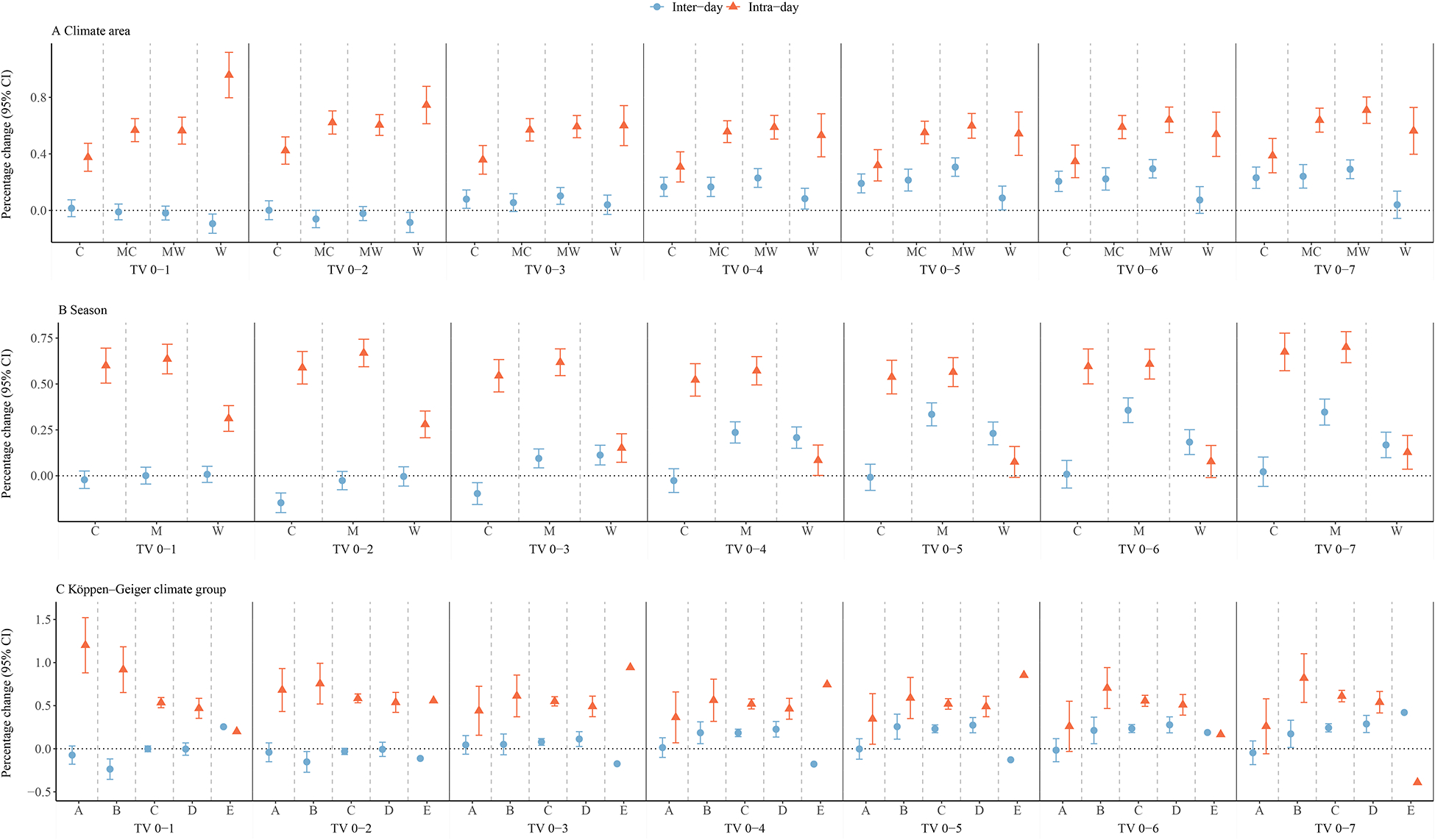
Overall percentage change (%) of mortality risk for inter-day and intra-day TV (TV_0–1_ to TV_0–7_) in different climate areas (A), seasons (B), and Köppen–Geiger climate groups (C). Definition of abbreviations: (A) C = cold, MC = moderate cold, MW = moderate warm, W = warm; (B) C = cold, M = moderate, W = warm; (C) A = tropical, B = dry, C = temperate, D = continental, and E = polar.

Fig. 3 shows the stratified results by climate areas, seasons, and Köppen-Geiger climate groups. The mortality risks related to intra-day TV_0–7_ were higher than the risks of inter-day TV_0–7_ in all climate areas while the difference between mortality risk of inter-day and intra-day TV_0–7_ was greater in the warm area. When stratified by season, lower mortality risks related to inter-day TV_0–7_ were observed in the moderate season and warm season compared with intra-day TV_0–7_, while inter-day TV_0–7_ was associated with slightly higher mortality risk in the cold season. When examining the effect estimates in different Köppen-Geiger climate groups, the differences in mortality risk of inter-day and intra-day TV_0–7_ were greater in the dry climate and the polar climate. The exact values of Fig. 3 were presented in supplementary Table S8–S16.

### 3.4. Geographical variations

**Fig. 4.**
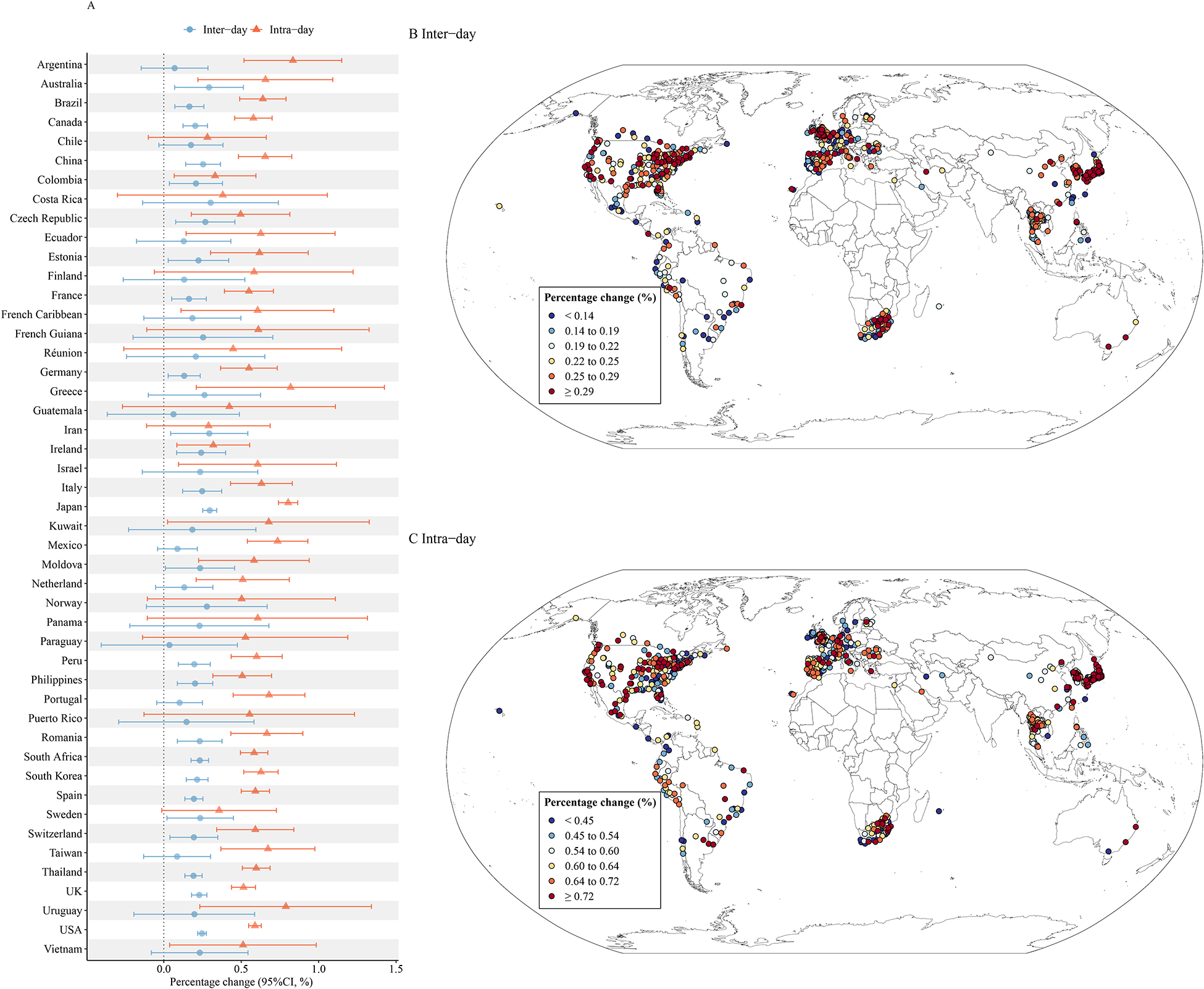
Overall percentage change (%) of mortality risk for inter-day and intra-day TV_0–7_ in all countries/regions (A) and locations (B and C).

Fig. 4 shows the mortality risks related to inter-day and intra-day TV_0–7_ in each country (Fig. 4A) and each location (Fig. 4B and 4C). The highest mortality risks associated with intra-day TV_0–7_ were found in Argentina, Greece, and Japan, whereas the lowest mortality risks were estimated for Ireland, Iran, and Chile. Referring to inter-day TV_0–7_, the highest mortality risks were found in Costa Rica, Iran, and Japan, while the lowest mortality risks were found in Argentina, Guatemala, and Paraguay. The highest mortality risks of inter-day TV_0–7_ and highest intra-day TV_0–7_ were mainly observed for locations in North America and Japan, while the lowest levels were mainly observed in Europe and South Africa.

### 3.5. Attributable burden

Table 2 depicts the mortality fractions attributable to the exposure of intra-day TV_0–7_ and inter-day TV_0–7_ and Table 3 provides the annual average attributable deaths. The attributable fractions and the annual average attributable deaths of TV_0–1_ to TV_0–6_ are shown in supplementary Table S17–S22. Overall, a total of 1.45 % (95 % CI: 1.31 %, 1.60 %) and 0.35 % (95 % CI: 0.29 %, 0.41 %) of all-cause deaths were estimated to be attributable to intra-day TV_0–7_ and inter-day TV_0–7_ exposure across 758 locations, corresponding to 76,598 attributable deaths per year (95 % CI: 69,023, 84,160) for intra-day TV_0–7_ and 17,120 attributable deaths per year (95 % CI: 14,170, 20,068) for inter-day TV_0–7_. Specifically, 1.57 % (95 % CI: 1.35 %, 1.79 %) of cardiovascular mortality and 1.57 % (95 % CI: 1.18 %, 1.96 %) of respiratory mortality were associated with exposure to intra-day TV_0–7_ while the percentages related to inter-day TV_0–7_ exposure were 0.66 % (95 %CI: 0.56 %, 0.76 %) for cardiovascular mortality and 0.44 % (95 % CI: 0.28 %, 0.60 %) respiratory mortality. The attributable fractions varied substantially across different countries, with the largest fractions for all-cause mortality related to intra-day TV_0–7_ observed in Iran, Romania, and South Africa. Referring to inter-day TV_0–7_ exposure, Paraguay, Canada, and the Czech Republic had the highest attributable fractions for all-cause, cardiovascular, and respiratory mortality.

### 3.6. Sensitivity analyses

Sensitivity analyses show that our results were robust when we assessed the relative risk associated with per 1 °C increase in inter-day or intra-day TV, changed the lag days or the df the lag-response curve for daily mean temperatures, and when we used daily minimum temperature or daily maximum temperature to replace the daily mean temperature (supplementary Figure S1–S5). The results did not change substantially when we performed separate models by including only one of the TV indicators and when we adjusted for RH in our models (supplementary Figure S6 and S7). The temporal trends of mortality risk are presented in supplementary Figure S8, showing there is a decrease in the mortality risk associated with both inter-day and intra-day TV over the study period.

## 4. Discussion

To the best of our knowledge, this is the first and largest study to investigate the associations of daily mortality with intra-day TV and inter-day TV on a global scale. Generally, increased risks for all-cause, cardiovascular, and respiratory mortality were observed to be significantly associated with exposure to inter-day and intra-day TVs. We estimated that a total of 1.45 % and 0.35 % of all-cause deaths could be attributed to intra-day TV_0–7_ and inter-day TV_0–7_, corresponding to 76,598 and 17,120 annual average attributable deaths, respectively. We found that the mortality risks related to inter-day and intra-day TVs varied by climate areas, seasons, and Köppen–Geiger climate types. This global study also recognized regions with higher mortality risks associated with inter-day and intra-day TVs.

For all-cause mortality, we observed an increase of 0.59 % and 0.22 % in mortality risk per IQR increase in intra-day TV_0–7_ and inter-day TV_0–7_, respectively, which were consistent with previous studies (Guoet al., 2016; Yanget al., 2018; Zhanget al., 2019). The magnitude of our effect estimates for inter-day or intra-day TVs was lower than that of the total TV estimated in our previous study (Guoet al., 2016). Compared with another previous study using DTR as the indicator of intra-day temperature variation, we found a smaller mortality risk as well as a smaller attributable fraction related to the exposure of intra-day TV (Leeet al., 2018). The first reason is that we used the minimum intra-day TV of each location as the reference values to calculate the AF, while previous studies commonly used zero (assuming there is no temperature change) as the reference value. Another reason could be related to the inclusion of more communities and countries, and the differences in the lag period and structure. In the previous study, a longer lag period (up to 14 days) with a flexible lag structure was applied to DTR while we considered the impacts of intra-day TV to be the same across 0–7 lag days (Leeet al., 2018).

Our study provides new insights into the comparison of the health impacts of intra-day and inter-day temperature variations. Different from previous studies using DTR and TCN as indicators, we applied two novel indices, intra-day TV and inter-day TV, which enable us to directly compare the health impacts under the same framework (Huet al. 2021; Vicedo-Cabreraet al., 2016). Generally, we found that intra-day TV was associated with a higher mortality risk compared with inter-day TV. The results indicated that intra-day TV may explain the main part of the mortality risk related to temperature variation. There were positive associations between intra-day TV and mortality risk across all lag intervals while the associations between inter-day TV and all-cause mortality risk were negative with a shorter lag interval (TV_0–1_ and TV_0–2_). This may be reasonable because previous studies found that a negative TCN (temperature decrease between neighbouring days) presented a protective effect on mortality risk (Xiaoet al., 2021; Zhanet al., 2017). The potential mechanisms of this protective effect are still unclear, but may be related to factors such as causes of death, climate, geographic location, and demographics (Zhanet al., 2017). For example, unlike all-cause mortality, short-term inter-day TV exposure (TV_0–1_ and TV_0–2_) can significantly increase the risk of cardiovascular death. However, our results showed that inter-day TV elevated mortality risk with longer lag intervals, which indicated that inter-day temperature variation within a longer period would be more harmful. Previous studies have observed that longer durations of TV tend to have greater health impacts (Yanget al., 2018; Yanget al., 2021). For example, Yang et al reported that the non-accidental mortality risk increased by 0.60 % with every 1 °C increase in TV_0–7_, while it was 0.35 % for TV_0–1_ (Yanget al., 2018). This is also observed for specific causes, including cardiovascular and respiratory mortality (Yanget al., 2018). Our findings suggested that increasing trend of the mortality risk from total TV may be mainly contributed by the inter-day component.

The stratified analyses demonstrated that the effect estimates for mortality risks related to inter-day and intra-day TVs varied by climate areas, seasons, and Köppen–Geiger climate types. We have previously observed that people living in moderate areas were more vulnerable to long TV exposure (Guoet al., 2016). This could be explained by decomposing the total TV into intra-day and inter-day parts. For example, we found that mortality risks of inter-day TV in moderate cold or moderate warm areas increased substantially with the increment of the lag interval while the risks of intra-day TV remain stable, which may be the main driver of the elevated effects of long TV exposure in moderate areas. The season could be another important modification factor for the TV-mortality association. Consistent with previous studies, we observed that intra-day TV led to higher mortality risks in the cold and the moderate season compared with the warm season (Phosriet al., 2020; Qiuet al., 2013). By contrast, the mortality risks of inter-day TV in the warm season showed an upward trend with the increment of the lag interval, which even exceeded the effects of intra-day TV (TV_0–4_ to TV_0–7_). Affected mainly by local air mass advection due to anomalous atmospheric circulation, inter-day TV with lag intervals of 3–7 days could contribute to the occurrence of heatwaves and cold spells and thus may cause greater health hazards.(Guoet al., 2021) The TV effects also varied in different Köppen–Geiger climate areas. Our results showed that intra-day TV had higher acute effects (TV_0–1_) in the tropical and dry climate, which was consistent with a previous study showing that people living in warmer and less humid regions were more vulnerable to high DTR(Zhanget al., 2018). This might stem from a combination of factors, including physiological, technological, and behavioural changes or adaptations in response to the climate (Leeet al., 2018). For instance, more water evaporation from the body could be observed in dry and hot environments, which would further restrict blood flow in the vascular system, potentially influencing disease susceptibility (Fanget al., 2023). These impacts could further heighten vulnerability to TV exposure.

Our results revealed that the TV-related mortality burden showed heterogeneity between regions and countries. For example, higher fractions of premature deaths attributable to intra-day TV were observed in Iran, Romania, and South Africa, which were twice as high as in countries with a lower burden. Similarly, we observed higher fractions of death attributable to inter-day TV in Paraguay and the USA. Generally, previous studies considering the health impacts of temperature variation were mainly conducted in East Asia, America, Europe, and Oceania (Chenget al., 2014; Guoet al., 2016; Leeet al., 2018). Our results, however, showed that attention should also be paid to regions like Africa and the Middle East. More importantly, research is urgently needed in low- and middle-income countries, where few comprehensive assessments on the health impacts of climate change have been completed (Costelloet al., 2009). It has been widely accepted that climate change would exacerbate health inequities between regions, countries, and communities (Romanelloet al., 2021). Thus, further research is required to tackle the health threat of temperature variation, including proposing a standardized monitoring and evaluation system with indicators for the health risks related to climate change, continuing training and capacity building in climate and health, and raising sufficient human and financial resources through government aid and fiscal policy (Costelloet al., 2009; Ebi and Otmani Del Barrio, 2017).

In this study, we quantified that 1.45 % and 0.35 % of all-cause death could be attributed to intra-day TV_0–7_ and inter-day TV_0–7_, respectively. While these values were lower than that reported for cold-related deaths (7.29 % in a prior study), they substantially exceeded the AF attributable to hot temperature (0.42 %) (Gasparriniet al., 2015). Currently, Heat-Health Warning Systems (HHWS) developed in many countries primarily rely on the mean temperature for reference value determination (Casanuevaet al., 2019). However, our estimations indicated that TV may lead to a comparable or even greater mortality burden compared to heat exposure. Notably, TV not only has a direct impact on health, but also interacts with ambient temperature, potentially amplifying its effect on mortality (Wu et al., 2022). Ignoring the effects of TV would underestimate the mortality burden attributable to extreme temperatures. Therefore, it is crucial to incorporate inter-day and intra-day TV into the existing early warning systems. By splitting TV into the inter-day and intra-day component within a unified framework, this study empowers policymakers to prioritize the metric with the greatest health impact according to the local climate, allowing for preventive measures and timely responses to mitigate adverse effects. For example, healthcare professionals can proactively identify vulnerable populations (e.g., children, the elderly, and those with underlying conditions) and collaborate with patients and their caregivers to implement primary and secondary prevention strategies (e.g., education, medication review/adjustment, and routine assessments) during periods of dramatical temperature fluctuation.

This study has some strengths. First, this is the first and largest global investigation of the health impacts of intra-day and inter-day TV. Using the MCC dataset from 758 communities of 47 countries or regions, our results could provide robust estimations and give a comprehensive evaluation on a global scale. Second, we applied a new method to decompose the TV into intra-day and inter-day parts based on the same framework, which enables us to compare the two indicators directly. However, several limitations should also be acknowledged. First, our study had insufficient coverage in less industrialized areas as only a few communities were included in regions like Africa and Central Asia. Thus, the current findings should be interpreted with caution. Secondly, exposure misclassification may be introduced as we used daily weather data at the community level. Third, we were unable to provide the gender- and age-specific associations due to the lack of demographic information. As a result, further investigations are needed to identify vulnerable populations in more countries. Furthermore, the inter-day and intra-day TV proposed in this study are incapable of capturing the direction of temperature change in a short period (typically within two days), while prior studies suggested that negative and positive TCN may exert differential mortality impacts (Xiaoet al., 2021; Zhanet al., 2017). Therefore, it is necessary to incorporate additional factors, such as season, climatic characteristics, and location, in the analysis of health impacts of inter-day TV.

In conclusion, the current multi-country time-series analysis provides evidence on the associations between the exposure to TV and daily all-cause, cardiovascular, and respiratory mortality. Our results indicated that intra-day TV may explain the main part of the mortality risk related to temperature variation while the mortality risks varied by lag interval, climate area, season, and climate type. The findings suggest that comprehensive evaluations should be proposed in more countries to help protect human health.

## Supplementary Material

Supplementary material

Appendix A. Supplementary data

Supplementary data to this article can be found online at https://doi.org/10.1016/j.envint.2024.108712.

## Figures and Tables

**Table 1.** Summary of study periods, number of deaths, median (IQR) of inter-day and intra-day TV in each country/region.

Country/region	No of locations	Period	Number of deaths (in thousands)			TV, Median (IQR)	
			Total	Cardiovascular	Respiratory	Inter-day TV (°C)	Intra-day TV (°C)
Argentina	3	2005–2015	686.3	NA	NA	2.6 (2.5, 2.7)	6.3 (5.1, 6.8)
Australia	3	1988–2009	1,178.00	NA	NA	1.7 (1.4, 2.4)	4.5 (4.2, 4.7)
Brazil	17	1997–2018	3,822.60	1,201.00	452.8	1.2 (0.9, 1.8)	5.2 (4.2, 5.8)
Canada	26	1986–2015	3,733.70	1,264.60	316.5	3.2 (2.9, 3.5)	5.1 (4.5, 5.7)
Chile	4	2004–2014	325.5	NA	NA	1.5 (0.9, 1.6)	5.6 (3.3, 6.3)
China	15	1996–2015	1,069.20	411.9	144.7	2.1 (1.9, 2.2)	5.1 (3.4, 5.7)
Colombia	5	1998–2013	956.5	267.9	99.8	0.8 (0.7, 0.9)	5.0 (3.8, 5.4)
Costa Rica	1	2000–2017	31.1	9.3	2.7	0.8 (0.8, 0.8)	4.5 (4.5, 4.6)
Czech Republic	4	1994–2015	711.9	360	39.9	2.6 (2.5, 2.7)	3.2 (3.0, 3.3)
Ecuador	2	2014–2018	112.3	32.8	13	0.9 (0.8, 1.0)	4.9 (3.7, 6.2)
Estonia	5	1997–2018	167.8	85.7	5.6	2.4 (2.3, 2.6)	4.2 (3.9, 4.4)
Finland	1	1994–2014	153.3	57.4	9.7	2.3 (2.1, 2.3)	3.4 (3.4, 3.6)
France	18	2000–2015	1,753.60	NA	109.9	2.1 (1.9, 2.3)	4.8 (4.1, 5.1)
French Caribbean	2	2000–2015	46.2	NA	NA	0.6 (0.6, 0.6)	3.8 (3.5, 3.9)
French Guiana	1	2000–2015	7.1	NA	NA	0.6 (0.5, 0.6)	4.1 (4.0, 4.2)
Réunion	1	2000–2015	13.9	NA	NA	0.7 (0.6, 0.7)	3.5 (3.4, 3.6)
Germany	12	1993–2015	3,105.90	NA	NA	2.3 (2.2, 2.4)	4.5 (4.3, 4.7)
Greece	1	2001–2010	288	136.2	28.8	1.8 (1.7, 1.8)	4.1 (4.0, 4.3)
Guatemala	1	2009–2016	62.7	NA	NA	0.9 (0.9, 1.0)	4.8 (4.6, 4.8)
Iran	2	2002–2015	817.9	357.7	59.4	2.2 (2.1, 2.3)	5.5 (5.2, 6.7)
Ireland	6	1984–2007	1,058.20	340.3	164.1	1.6 (1.5, 1.8)	3.6 (3.2, 3.9)
Israel	1	1985–2020	350.6	NA	NA	1.4 (1.3, 1.5)	5.7 (5.6, 5.9)
Italy	11	1987–2010	820.4	NA	NA	1.5 (1.4, 1.6)	4.4 (3.7, 4.9)
Japan	47	1972–2015	39,917.60	13,631.40	5,028.30	1.8 (1.7, 1.9)	4.5 (4.1, 5.0)
Kuwait	1	2000–2016	73.7	35.3	5.7	1.3 (1.3, 1.3)	4.3 (4.3, 4.4)
Mexico	10	1998–2014	2,980.10	765.2	284.2	1.4 (1.0, 1.9)	7.0 (6.2, 7.5)
Moldova	4	2001–2010	59.9	NA	NA	2.5 (2.4, 2.6)	5.2 (4.7, 5.7)
Netherland	5	1995–2016	453.4	NA	NA	2.1 (2.0, 2.2)	4.2 (4.0, 4.5)
Norway	1	1979–2018	212.1	79.8	21.1	2.2 (2.1, 2.3)	3.8 (3.7, 3.9)
Panama	1	2013–2016	11.5	3.9	1	0.8 (0.8, 0.8)	4.0 (3.9, 4.1)
Paraguay	1	2004–2019	48	15.4	4.4	2.7 (2.7, 2.8)	5.7 (5.6, 5.7)
Peru	18	2008–2014	633.1	NA	NA	0.9 (0.8, 1.0)	6.2 (5.1, 7.2)
Philippines	13	2006–2019	821.5	296.9	118.5	0.6 (0.6, 0.7)	2.8 (2.7, 2.8)
Portugal	6	1980–2018	1,925.30	718.6	181.4	1.8 (1.6, 1.9)	5.0 (4.6, 5.7)
Puerto Rico	1	2009–2016	26.6	NA	NA	0.7 (0.7, 0.7)	3.5 (3.4, 3.6)
Romania	8	1994–2016	951.1	NA	NA	2.3 (2.2, 2.5)	5.6 (5.2, 6.1)
South Africa	52	1997–2013	8,509.10	1,299.70	1,046.90	2.0 (1.7, 2.4)	7.5 (6.8, 8.4)
South Korea	36	1997–2018	3,070.40	701.6	222.3	2.1 (1.9, 2.2)	5.3 (4.4, 6.1)
Spain	52	1990–2014	3,017.10	1,042.00	340.5	1.7 (1.4, 1.9)	5.1 (3.6, 5.7)
Sweden	3	1990–2016	717.3	310.8	54.9	1.9 (1.8, 2.0)	3.6 (3.4, 3.8)
Switzerland	8	1995–2013	243.6	90.7	16	2.2 (2.0, 2.3)	4.1 (3.7, 4.5)
Taiwan	3	1994–2014	1,209.60	269.4	116.5	1.5 (1.3, 1.8)	3.7 (3.4, 4.4)
Thailand	62	1999–2008	1,827.90	338.6	225.4	0.9 (0.7, 1.1)	5.2 (4.6, 5.6)
UK	70	1990–2016	6,167.10	2,258.30	910.4	1.7 (1.6, 1.9)	3.3 (2.8, 3.5)
Uruguay	1	2012–2016	153.6	NA	NA	2.3 (2.2, 2.3)	4.1 (4.1, 4.3)
USA	211	1979–2006	32,194.90	11,482.40	2,941.50	2.8 (2.2, 3.1)	5.1 (4.5, 5.6)
Vietnam	2	2009–2013	108.2	24.4	9	1.3 (0.8, 1.4)	4.4 (4.3, 4.5)
Pooled	758	1972–2020	126,605.10	37,889.30	12,975.00	2.0 (1.7, 2.6)	4.9 (3.9, 5.5)

Note. NA means data are unavailable in this country or region.

**Table 2.** Attributable fractions (%) of both inter-day TV_0–7_ and intra-day TV_0–7_ in each country/region.

Country/region	All-cause mortality		Cardiovascular mortality		Respiratory mortality	
	Inter-day	Intra-day	Inter-day	Intra-day	Inter-day	Intra-day
Argentina	0.43 (0.35, 0.50)	1.65 (1.49, 1.81)	NA	NA	NA	NA
Australia	0.35 (0.29, 0.40)	1.43 (1.29, 1.57)	NA	NA	NA	NA
Brazil	0.26 (0.21, 0.30)	1.61 (1.45, 1.77)	0.50 (0.42, 0.58)	1.82 (1.56, 2.07)	0.35 (0.22, 0.47)	1.76 (1.32, 2.20)
Canada	0.51 (0.43, 0.60)	1.67 (1.51, 1.84)	0.96 (0.82, 1.11)	1.87 (1.61, 2.13)	0.68 (0.44, 0.92)	1.78 (1.34, 2.22)
Chile	0.21 (0.17, 0.25)	1.75 (1.58, 1.92)	NA	NA	NA	NA
China	0.32 (0.26, 0.37)	1.13 (1.02, 1.24)	0.61 (0.52, 0.71)	1.31 (1.13, 1.50)	0.40 (0.26, 0.54)	1.11 (0.84, 1.39)
Colombia	0.14 (0.12, 0.17)	1.21 (1.09, 1.33)	0.27 (0.23, 0.31)	1.35 (1.16, 1.54)	0.19 (0.12, 0.25)	1.31 (0.99, 1.64)
Costa Rica	0.12 (0.10, 0.14)	1.40 (1.26, 1.54)	0.22 (0.19, 0.25)	1.57 (1.35, 1.79)	0.16 (0.10, 0.21)	1.50 (1.12, 1.87)
Czech Republic	0.44 (0.37, 0.52)	1.25 (1.13, 1.37)	0.82 (0.70, 0.95)	1.39 (1.20, 1.59)	0.58 (0.37, 0.79)	1.33 (1.00, 1.66)
Ecuador	0.13 (0.11, 0.15)	1.19 (1.07, 1.31)	0.24 (0.20, 0.28)	1.27 (1.09, 1.45)	0.17 (0.11, 0.23)	1.30 (0.98, 1.63)
Estonia	0.39 (0.32, 0.46)	1.39 (1.25, 1.53)	0.74 (0.62, 0.85)	1.55 (1.33, 1.77)	0.52 (0.34, 0.71)	1.50 (1.13, 1.88)
Finland	0.40 (0.33, 0.47)	1.30 (1.17, 1.43)	0.75 (0.63, 0.86)	1.46 (1.26, 1.67)	0.55 (0.35, 0.74)	1.42 (1.06, 1.77)
France	0.34 (0.28, 0.40)	1.54 (1.39, 1.69)	NA	NA	0.45 (0.29, 0.61)	1.62 (1.22, 2.03)
French Caribbean	0.10 (0.08, 0.11)	1.15 (1.04, 1.27)	NA	NA	NA	NA
French Guiana	0.09 (0.07, 0.11)	1.45 (1.30, 1.59)	NA	NA	NA	NA
Réunion	0.10 (0.09, 0.12)	0.89 (0.80, 0.97)	NA	NA	NA	NA
Germany	0.39 (0.32, 0.45)	1.55 (1.40, 1.70)	NA	NA	NA	NA
Greece	0.31 (0.26, 0.36)	1.52 (1.37, 1.67)	0.58 (0.49, 0.67)	1.70 (1.46, 1.93)	0.40 (0.26, 0.54)	1.65 (1.24, 2.06)
Guatemala	0.14 (0.12, 0.17)	1.34 (1.21, 1.47)	NA	NA	NA	NA
Iran	0.38 (0.31, 0.44)	1.95 (1.76, 2.14)	0.70 (0.59, 0.81)	2.14 (1.84, 2.44)	0.50 (0.32, 0.68)	2.05 (1.54, 2.56)
Ireland	0.28 (0.23, 0.33)	1.16 (1.05, 1.28)	0.52 (0.44, 0.60)	1.29 (1.11, 1.48)	0.37 (0.24, 0.50)	1.25 (0.94, 1.56)
Israel	0.26 (0.21, 0.30)	1.41 (1.27, 1.55)	NA	NA	NA	NA
Italy	0.27 (0.22, 0.31)	1.56 (1.41, 1.71)	NA	NA	NA	NA
Japan	0.32 (0.26, 0.37)	1.34 (1.21, 1.48)	0.59 (0.50, 0.69)	1.51 (1.30, 1.72)	0.41 (0.26, 0.56)	1.45 (1.09, 1.81)
Kuwait	0.25 (0.21, 0.29)	1.14 (1.03, 1.25)	0.47 (0.40, 0.54)	1.28 (1.10, 1.46)	0.33 (0.21, 0.45)	1.21 (0.91, 1.52)
Mexico	0.23 (0.19, 0.27)	1.81 (1.63, 1.99)	0.43 (0.37, 0.50)	2.02 (1.74, 2.30)	0.30 (0.19, 0.41)	2.01 (1.51, 2.51)
Moldova	0.41 (0.34, 0.48)	1.70 (1.53, 1.87)	NA	NA	NA	NA
Netherland	0.35 (0.29, 0.41)	1.39 (1.25, 1.52)	NA	NA	NA	NA
Norway	0.40 (0.33, 0.46)	1.42 (1.28, 1.56)	0.74 (0.63, 0.86)	1.59 (1.37, 1.82)	0.54 (0.35, 0.73)	1.53 (1.15, 1.91)
Panama	0.15 (0.13, 0.18)	0.96 (0.87, 1.06)	0.29 (0.24, 0.33)	1.07 (0.92, 1.22)	0.19 (0.12, 0.26)	1.01 (0.76, 1.26)
Paraguay	0.54 (0.45, 0.63)	1.73 (1.56, 1.90)	1.02 (0.86, 1.17)	1.93 (1.66, 2.20)	0.72 (0.46, 0.98)	1.87 (1.40, 2.33)
Peru	0.12 (0.10, 0.14)	1.61 (1.45, 1.77)	NA	NA	NA	NA
Philippines	0.10 (0.09, 0.12)	0.82 (0.74, 0.90)	0.19 (0.16, 0.22)	0.92 (0.79, 1.05)	0.13 (0.09, 0.18)	0.88 (0.66, 1.10)
Portugal	0.30 (0.24, 0.35)	1.75 (1.58, 1.93)	0.55 (0.46, 0.63)	1.95 (1.67, 2.22)	0.39 (0.25, 0.52)	1.87 (1.41, 2.34)
Puerto Rico	0.11 (0.09, 0.12)	1.05 (0.95, 1.15)	NA	NA	NA	NA
Romania	0.42 (0.35, 0.49)	2.15 (1.94, 2.36)	NA	NA	NA	NA
South Africa	0.34 (0.28, 0.40)	2.24 (2.02, 2.47)	0.63 (0.53, 0.72)	2.53 (2.18, 2.89)	0.44 (0.28, 0.60)	2.53 (1.90, 3.15)
South Korea	0.37 (0.31, 0.44)	1.36 (1.22, 1.49)	0.70 (0.59, 0.81)	1.52 (1.30, 1.73)	0.49 (0.32, 0.67)	1.50 (1.13, 1.87)
Spain	0.27 (0.22, 0.31)	1.63 (1.47, 1.79)	0.50 (0.42, 0.57)	1.81 (1.55, 2.06)	0.35 (0.23, 0.48)	1.80 (1.35, 2.24)
Sweden	0.33 (0.27, 0.39)	1.29 (1.16, 1.42)	0.62 (0.52, 0.72)	1.44 (1.24, 1.64)	0.44 (0.28, 0.60)	1.38 (1.04, 1.73)
Switzerland	0.37 (0.31, 0.43)	1.41 (1.27, 1.55)	0.69 (0.58, 0.80)	1.57 (1.35, 1.79)	0.48 (0.31, 0.65)	1.50 (1.12, 1.87)
Taiwan	0.28 (0.23, 0.33)	1.26 (1.14, 1.39)	0.54 (0.46, 0.62)	1.43 (1.22, 1.63)	0.37 (0.24, 0.51)	1.40 (1.05, 1.74)
Thailand	0.16 (0.13, 0.19)	1.31 (1.18, 1.44)	0.30 (0.25, 0.34)	1.46 (1.26, 1.67)	0.21 (0.13, 0.28)	1.43 (1.07, 1.78)
UK	0.29 (0.24, 0.34)	1.15 (1.04, 1.27)	0.55 (0.46, 0.63)	1.28 (1.10, 1.46)	0.39 (0.25, 0.53)	1.22 (0.91, 1.52)
Uruguay	0.34 (0.28, 0.40)	1.33 (1.20, 1.46)	NA	NA	NA	NA
USA	0.43 (0.36, 0.51)	1.35 (1.22, 1.49)	0.81 (0.69, 0.94)	1.50 (1.29, 1.71)	0.57 (0.37, 0.78)	1.49 (1.12, 1.86)
Vietnam	0.13 (0.11, 0.16)	1.17 (1.06, 1.29)	0.26 (0.22, 0.30)	1.34 (1.15, 1.52)	0.17 (0.11, 0.23)	1.25 (0.94, 1.56)
Pooled	0.35 (0.29, 0.41)	1.45 (1.31, 1.60)	0.66 (0.56, 0.76)	1.57 (1.35, 1.79)	0.44 (0.28, 0.60)	1.57 (1.18, 1.96)

Note. NA means data are unavailable in this country or region.

**Table 3.** Annual average attributable deaths of inter-day TV_0–7_ and intra-day TV_0–7_ in each country/region.

Country/region	All-cause mortality		Cardiovascular mortality		Respiratory mortality	
	Inter-day	Intra-day	Inter-day	Intra-day	Inter-day	Intra-day
Argentina	265 (220, 311)	1,029 (928, 1,131)	NA	NA	NA	NA
Australia	185 (153, 217)	764 (688, 839)	NA	NA	NA	NA
Brazil	464 (384, 544)	2,871 (2,587, 3,154)	281 (238, 324)	1,018 (875, 1,161)	73 (47, 99)	371 (279, 464)
Canada	640 (530, 750)	2,079 (1,874, 2,285)	407 (344, 469)	788 (678, 899)	72 (46, 97)	188 (141, 234)
Chile	93 (77, 109)	783 (706, 860)	NA	NA	NA	NA
China	766 (634, 898)	2,908 (2,621, 3,196)	592 (501, 683)	1,348 (1,158, 1,537)	126 (81, 171)	382 (287, 477)
Colombia	86 (71, 101)	726 (654, 798)	45 (38, 52)	227 (195, 259)	12 (7, 16)	82 (61, 102)
Costa Rica	2 (2, 2)	24 (22, 27)	1 (1, 1)	8 (7, 9)	0 (0, 0)	2 (2, 3)
Czech Republic	143 (118, 168)	405 (365, 445)	135 (114, 155)	228 (196, 260)	11 (7, 14)	24 (18, 30)
Ecuador	30 (24, 35)	267 (241, 294)	16 (13, 18)	84 (72, 95)	4 (3, 6)	34 (25, 42)
Estonia	30 (25, 35)	106 (96, 116)	29 (24, 33)	60 (52, 69)	1 (1, 2)	4 (3, 5)
Finland	29 (24, 34)	95 (86, 104)	20 (17, 24)	40 (34, 46)	3 (2, 3)	7 (5, 8)
France	373 (309, 438)	1,688 (1,521, 1,855)	NA	NA	31 (20, 42)	112 (84, 139)
French Caribbean	3 (2, 3)	33 (30, 37)	NA	NA	NA	NA
French Guiana	0 (0, 0)	6 (6, 7)	NA	NA	NA	NA
Réunion	1 (1, 1)	8 (7, 8)	NA	NA	NA	NA
Germany	521 (431, 611)	2,091 (1,884, 2,297)	NA	NA	NA	NA
Greece	89 (73, 104)	439 (395, 482)	79 (67, 91)	231 (198, 263)	12 (7, 16)	47 (36, 59)
Guatemala	11 (9, 13)	105 (95, 116)	NA	NA	NA	NA
Iran	232 (192, 272)	1,228 (1,107, 1,349)	187 (159, 216)	581 (500, 662)	22 (14, 30)	92 (69, 115)
Ireland	123 (102, 144)	514 (463, 565)	74 (62, 85)	184 (158, 209)	25 (16, 34)	85 (64, 107)
Israel	25 (21, 29)	137 (124, 151)	NA	NA	NA	NA
Italy	141 (117, 166)	786 (708, 863)	NA	NA	NA	NA
Japan	2,869 (2,374, 3,363)	12,185 (10,978, 13,389)	1,841 (1,558, 2,124)	4,686 (4,026, 5,343)	471 (302, 640)	1,660 (1,246, 2,073)
Kuwait	11 (9, 13)	50 (45, 54)	10 (8, 11)	26 (23, 30)	1 (1, 2)	4 (3, 5)
Mexico	398 (329, 466)	3,177 (2,863, 3,490)	195 (165, 226)	909 (782, 1,037)	50 (32, 68)	337 (253, 420)
Moldova	25 (21, 30)	104 (94, 114)	NA	NA	NA	NA
Netherland	72 (60, 84)	286 (258, 314)	NA	NA	NA	NA
Norway	21 (17, 25)	76 (68, 83)	15 (13, 17)	32 (27, 36)	3 (2, 4)	8 (6, 10)
Panama	4 (4, 5)	28 (25, 30)	3 (2, 3)	10 (9, 12)	0 (0, 1)	2 (2, 3)
Paraguay	16 (13, 19)	52 (47, 57)	10 (8, 11)	19 (16, 21)	2 (1, 3)	5 (4, 6)
Peru	109 (90, 128)	1,458 (1,314, 1,601)	NA	NA	NA	NA
Philippines	75 (62, 88)	582 (524, 640)	50 (43, 58)	235 (202, 268)	14 (9, 19)	90 (68, 113)
Portugal	146 (121, 171)	865 (780, 951)	101 (85, 117)	358 (308, 409)	18 (11, 24)	87 (65, 109)
Puerto Rico	3 (3, 4)	35 (31, 38)	NA	NA	NA	NA
Romania	173 (143, 202)	889 (801, 976)	NA	NA	NA	NA
South Africa	1,689 (1,398, 1,980)	11,236 (10,129, 12,341)	479 (405, 553)	1,938 (1,666, 2,208)	270 (173, 367)	1,557 (1,170, 1,941)
South Korea	518 (429, 607)	1,892 (1,705, 2,079)	223 (188, 257)	484 (416, 552)	50 (32, 68)	152 (114, 189)
Spain	324 (268, 380)	1,967 (1,773, 2,161)	208 (176, 239)	753 (647, 858)	48 (31, 66)	245 (184, 305)
Sweden	88 (73, 103)	343 (309, 377)	71 (60, 82)	166 (143, 189)	9 (6, 12)	28 (21, 35)
Switzerland	47 (39, 55)	181 (163, 199)	33 (28, 38)	75 (64, 85)	4 (3, 5)	13 (9, 16)
Taiwan	162 (134, 190)	728 (656, 800)	69 (59, 80)	183 (157, 209)	21 (13, 28)	77 (58, 97)
Thailand	294 (243, 344)	2,388 (2,152, 2,624)	100 (85, 116)	496 (426, 565)	47 (30, 63)	322 (242, 402)
UK	668 (553, 783)	2,635 (2,374, 2,896)	456 (386, 526)	1,073 (922, 1,224)	131 (84, 179)	410 (308, 512)
Uruguay	105 (87, 123)	408 (368, 449)	NA	NA	NA	NA
USA	5,015 (4,151, 5,878)	15,629 (14,082, 17,173)	3,347 (2,832, 3,860)	6,173 (5,305, 7,040)	603 (387, 819)	1,571 (1,179, 1,961)
Vietnam	35 (29, 41)	312 (281, 342)	15 (13, 18)	79 (68, 91)	4 (2, 5)	28 (21, 35)
Pooled	17,120 (14,170, 20,068)	76,598 (69,023, 84,160)	9,091 (7,692, 10,487)	22,492 (19,329, 25,645)	2,137 (1,369, 2,903)	8,027 (6,027, 10,017)

Note. NA means data are unavailable in this country or region.

## Data Availability

Data were collected within the MCC Collaborative Research Network under a data sharing agreement and cannot be made publicly available. Researchers can refer to MCC participants, who are listed as coauthors of this Article, for information on accessing the data for each country.

## Data availability

The authors do not have permission to share data.

## References

[R1] Bell ML, Dominici F, Samet JM, 2005. A meta-analysis of time-series studies of ozone and mortality with comparison to the national morbidity, mortality, and air pollution study. Epidimiology 16, 436–445. 10.1097/01.ede.0000165817.40152.85 PMC3581312 15951661

[R2] Casanueva A, Burgstall A, Kotlarski S, Messeri A, Morabito M, Flouris AD, Nybo L, Spirig C, Schwierz C, 2019. Overview of existing heat-health warning systems in Europe. Int. J. Environ. Res. Public Health10.3390/ijerph16152657 PMC6695887 31349585

[R3] Chen K, Breitner S, Wolf K, Stafoggia M, Sera F, Vicedo-Cabrera AM, Guo Y, Tong S, Lavigne E, Matus P, Valdes N, Kan H, Jaakkola JJK, Ryti NRI, Huber V, Scortichini M, Hashizume M, Honda Y, Nunes B, Madureira J, Holobaca IH, Fratianni S, Kim H, Lee W, Tobias A, Iniguez C, Forsberg B, Astrom C, Ragettli MS, Guo YL, Chen BY, Li S, Milojevic A, Zanobetti A, Schwartz J, Bell ML, Gasparrini A, Schneider A, 2021. Ambient carbon monoxide and daily mortality: a global time-series study in 337 cities. Lancet Planet Health 5, e191–e199. 33838734 10.1016/S2542-5196(21)00026-7

[R4] Cheng J, Xu Z, Zhu R, Wang X, Jin L, Song J, Su H, 2014. Impact of diurnal temperature range on human health: a systematic review. Int. J. Biometeorol 58, 2011–2024. 24535132 10.1007/s00484-014-0797-5

[R5] Costello A, Abbas M, Allen A, Ball S, Bell S, Bellamy R, Friel S, Groce N, Johnson A, Kett M, Lee M, Levy C, Maslin M, McCoy D, McGuire B, Montgomery H, Napier D, Pagel C, Patel J, De Oliveira JAP, Redclift N, Rees H, Rogger D, Scott J, Stephenson J, Twigg J, Wolff J, Patterson C, 2009. Managing the health effects of climate change. Lancet 373, 1693–1733. 19447250 10.1016/S0140-6736(09)60935-1

[R6] Ebi KL, Otmani Del Barrio M, 2017. Lessons learned on health adaptation to climate variability and change: experiences across low- and middle-income countries. Environ. Health Perspect 125, 065001. 28632491 10.1289/EHP405 PMC5743455

[R7] Fang W, Li Z, Gao J, Meng R, He G, Hou Z, Zhu S, Zhou M, Zhou C, Xiao Y, Yu M, Huang B, Xu X, Lin L, Xiao J, Jin D, Qin M, Yin P, Xu Y, Hu J, Liu T, Huang C, Ma W, 2023. The joint and interaction effect of high temperature and humidity on mortality in China. Environ. Int 171, 107669. 36508749 10.1016/j.envint.2022.107669

[R8] Gasparrini A, Armstrong B, Kenward MG, 2010. Distributed lag non-linear models. Stat. Med 29, 2224–2234. 20812303 10.1002/sim.3940 PMC2998707

[R9] Gasparrini A, Armstrong B, Kenward MG, 2012. Multivariate meta-analysis for non-linear and other multi-parameter associations. Stat. Med 31, 3821–3839. 22807043 10.1002/sim.5471 PMC3546395

[R10] Gasparrini A, Guo Y, Hashizume M, Lavigne E, Zanobetti A, Schwartz J, Tobias A, Tong S, Rocklov J, Forsberg B, Leone M, De Sario M, Bell ML, Guo YL, Wu CF, Kan H, Yi SM, de Sousa Zanotti Stagliorio Coelho M, Saldiva PH, Honda Y, Kim H, Armstrong B, 2015. Mortality risk attributable to high and low ambient temperature: a multicountry observational study. Lancet 386, 369–375. 26003380 10.1016/S0140-6736(14)62114-0 PMC4521077

[R11] Guo F, Do V, Cooper R, Huang Y, Zhang P, Ran J, Zhang Q, Tian L, Fu Z, 2021. Trends of temperature variability: Which variability and what health implications? Sci. Total Environ 768, 144487. 33444866 10.1016/j.scitotenv.2020.144487

[R12] Guo Y, Gasparrini A, Armstrong BG, Tawatsupa B, Tobias A, Lavigne E, Coelho MS, Pan X, Kim H, Hashizume M, Honda Y, Guo YL, Wu CF, Zanobetti A, Schwartz JD, Bell ML, Overcenco A, Punnasiri K, Li S, Tian L, Saldiva P, Williams G, Tong S, 2016. Temperature variability and mortality: a multi-country study. Environ. Health Perspect 124, 1554–1559. 27258598 10.1289/EHP149 PMC5047764

[R13] Hu Y, Cheng J, Yin Y, Liu S, Tan J, Li S, Wu M, Yan C, Yu G, Hu Y, Tong S, 2021. Association of childhood asthma with intra-day and inter-day temperature variability in Shanghai, China. Environ. Res 112350. 34762926 10.1016/j.envres.2021.112350

[R14] IPCC. Managing the Risks of Extreme Events and Disasters to Advance Climate Change Adaptation Special Report of the Intergovernmental Panel on Climate Change Preface. Cambridge University Press 2012:582 pp.

[R15] Lee W, Bell ML, Gasparrini A, Armstrong BG, Sera F, Hwang S, Lavigne E, Zanobetti A, Coelho MDSZS, Saldiva PHN, Osorio S, Tobias A, Zeka A, Goodman PG, Forsberg B, Rocklöv J, Hashizume M, Honda Y, Guo Y-L-L, Seposo X, Van Dung D, Dang TN, Tong S, Guo Y, Kim H, 2018. Mortality burden of diurnal temperature range and its temporal changes: A multi-country study. Environ. Int 110, 123–130. 29089167 10.1016/j.envint.2017.10.018

[R16] Lin H, Zhang Y, Xu Y, Xu X, Liu T, Luo Y, Xiao J, Wu W, Ma W, 2013. Temperature changes between neighboring days and mortality in summer: a distributed lag non-linear time series analysis. PLoS One 8, e66403. 23826095 10.1371/journal.pone.0066403 PMC3691212

[R17] Liu C, Chen R, Sera F, Vicedo-Cabrera AM, Guo Y, Tong S, Coelho M, Saldiva PHN, Lavigne E, Matus P, Valdes Ortega N, Osorio Garcia S, Pascal M, Stafoggia M, Scortichini M, Hashizume M, Honda Y, Hurtado-Diaz M, Cruz J, Nunes B, Teixeira JP, Kim H, Tobias A, Iniguez C, Forsberg B, Astrom C, Ragettli MS, Guo YL, Chen BY, Bell ML, Wright CY, Scovronick N, Garland RM, Milojevic A, Kysely J, Urban A, Orru H, Indermitte E, Jaakkola JJK, Ryti NRI, Katsouyanni K, Analitis A, Zanobetti A, Schwartz J, Chen J, Wu T, Cohen A, Gasparrini A, Kan H, 2019. Ambient particulate air pollution and daily mortality in 652 cities. N. Engl. J. Med 381, 705–715. 31433918 10.1056/NEJMoa1817364 PMC7891185

[R18] Ma Y, Jiao H, Zhang Y, Cheng B, Feng F, Yu Z, Ma B, 2020. Impact of temperature changes between neighboring days on COPD in a city in Northeast China. Environ. Sci. Pollut. Res 27, 4849–4857. 10.1007/s11356-019-07313-1 31845269

[R19] Phosri A, Sihabut T, Jaikanlaya C, 2020. Short-term effects of diurnal temperature range on hospital admission in Bangkok, Thailand. Sci. Total Environ 717, 137202. 32062282 10.1016/j.scitotenv.2020.137202

[R20] Qiu H, Tak-Sun Yu I, Tse LA, Tian L, Wang X, Wong TW, 2013. Is greater temperature change within a day associated with increased emergency hospital admissions for heart failure? Circulat Heart Failure 6, 930–935. 10.1161/CIRCHEARTFAILURE.113.000360 23935005

[R21] Romanello M, McGushin A, Di Napoli C, Drummond P, Hughes N, Jamart L, Kennard H, Lampard P, Solano Rodriguez B, Arnell N, Ayeb-Karlsson S, Belesova K, Cai W, Campbell-Lendrum D, Capstick S, Chambers J, Chu L, Ciampi L, Dalin C, Dasandi N, Dasgupta S, Davies M, Dominguez-Salas P, Dubrow R, Ebi KL, Eckelman M, Ekins P, Escobar LE, Georgeson L, Grace D, Graham H, Gunther SH, Hartinger S, He K, Heaviside C, Hess J, Hsu S-C, Jankin S, Jimenez MP, Kelman I, Kiesewetter G, Kinney PL, Kjellstrom T, Kniveton D, Lee JKW, Lemke B, Liu Y, Liu Z, Lott M, Lowe R, Martinez-Urtaza J, Maslin M, McAllister L, McMichael C, Mi Z, Milner J, Minor K, Mohajeri N, Moradi-Lakeh M, Morrissey K, Munzert S, Murray KA, Neville T, Nilsson M, Obradovich N, Sewe MO, Oreszczyn T, Otto M, Owfi F, Pearman O, Pencheon D, Rabbaniha M, Robinson E, Rocklöv J, Salas RN, Semenza JC, Sherman J, Shi L, Springmann M, Tabatabaei M, Taylor J, Trinanes J, Shumake-Guillemot J, Vu B, Wagner F, Wilkinson P, Winning M, Yglesias M, Zhang S, Gong P, Montgomery H, Costello A, Hamilton IT, 2021. report of the Lancet Countdown on health and climate change: code red for a healthy future. Lancet 2021 (398), 1619–1662. 10.1016/S0140-6736(21)01787-6 PMC7616807 34687662

[R22] Sera F, Armstrong B, Blangiardo M, Gasparrini A, 2019. An extended mixed-effects framework for meta-analysis. Stat. Med 38, 5429–5444. 31647135 10.1002/sim.8362

[R23] Stott P, 2016. How climate change affects extreme weather events. Science 352, 1517–1518. 27339968 10.1126/science.aaf7271

[R24] Vicedo-Cabrera AM, Forsberg B, Tobias A, Zanobetti A, Schwartz J, Armstrong B, Gasparrini A, 2016. Associations of inter- and intraday temperature change with mortality. Am. J. Epidemiol 183, 286–293. 26811244 10.1093/aje/kwv205 PMC4753281

[R25] Vicedo-Cabrera AM, Sera F, Liu C, Armstrong B, Milojevic A, Guo Y, Tong S, Lavigne E, Kysely J, Urban A, Orru H, Indermitte E, Pascal M, Huber V, Schneider A, Katsouyanni K, Samoli E, Stafoggia M, Scortichini M, Hashizume M, Honda Y, Ng CFS, Hurtado-Diaz M, Cruz J, Silva S, Madureira J, Scovronick N, Garland RM, Kim H, Tobias A, Iniguez C, Forsberg B, Astrom C, Ragettli MS, Roosli M, Guo YL, Chen BY, Zanobetti A, Schwartz J, Bell ML, Kan H, Gasparrini A, 2020. Short term association between ozone and mortality: global two stage time series study in 406 locations in 20 countries. BMJ (clinical Research Ed) 368, m108. 10.1136/bmj.m108 PMC7190035 32041707

[R26] Wen B, Wu Y, Guo Y, Li S, 2023. A new method to separate the impacts of interday and intraday temperature variability on mortality. BMC Med. Res. Method 23. 10.1186/s12874-023-01914-8 PMC10105159 37061686

[R27] Wu Y, Wen B, Li S, Gasparrini A, Tong S, Overcenco A, Urban A, Schneider A, Entezari A, Vicedo-Cabrera AM, Zanobetti A, Analitis A, Zeka A, Tobias A, Alahmad B, Armstrong B, Forsberg B, Íñiguez C, Ameling C, De La Cruz Valencia C, Åström C, Houthuijs D, Van Dung D, Royé D, Indermitte E, Lavigne E, Mayvaneh F, Acquaotta F, De’Donato F, Sera F, Carrasco G, Kan H, Orru H, Kim H, Holobaca I-H, Kyselý J, Madureira J, Schwartz J, Katsouyanni K, Hurtado-Diaz M, Ragettli MS, Hashizume M, Pascal M, De Sousa Zanotti Stagliorio Coélho M, Scovronick N, Michelozzi P, Goodman P, Nascimento Saldiva PH, Abrutzky R, Osorio S, Dang TN, Colistro V, Huber V, Lee W, Seposo X, Honda Y, Bell ML, Guo Y Fluctuating temperature modifies heat-mortality association in the globe. The Innovation 2022:100225. 35340394 10.1016/j.xinn.2022.100225 PMC8942841

[R28] Xiao Y, Meng C, Huang S, Duan Y, Liu G, Yu S, Peng J, Cheng J, Yin P, 2021. Short-term effect of temperature change on non-accidental mortality in Shenzhen, China. Int. J. Environ. Res. Public Health 18, 8760. 34444520 10.3390/ijerph18168760 PMC8392083

[R29] Xu R, Zhao Q, Coelho MSZS, Saldiva PHN, Abramson MJ, Li S, Guo Y, 2019. The association between heat exposure and hospitalization for undernutrition in Brazil during 2000–2015: A nationwide case-crossover study. Plos Med 16, e1002950. 31661490 10.1371/journal.pmed.1002950 PMC6818759

[R30] Xu R, Zhao Q, Coelho MSZS, Saldiva PHN, Abramson MJ, Li S, Guo Y, 2020. Socioeconomic inequality in vulnerability to all-cause and cause-specific hospitalisation associated with temperature variability: a time-series study in 1814 Brazilian cities. The Lancet Planetary Health 4, e566–e576. 33278374 10.1016/S2542-5196(20)30251-5

[R31] Yang Z, Yang J, Zhou M, Yin P, Chen Z, Zhao Q, Hu K, Liu Q, Ou C-Q, 2021. Hourly temperature variability and mortality in 31 major Chinese cities: Effect modification by individual characteristics, season and temperature zone. Environ. Int 156, 106746. 34247007 10.1016/j.envint.2021.106746

[R32] Yang J, Zhou M, Li M, Liu X, Yin P, Sun Q, Wang J, Wu H, Wang B, Liu Q, 2018. Vulnerability to the impact of temperature variability on mortality in 31 major Chinese cities. Environ. Pollut 239, 631–637. 29709834 10.1016/j.envpol.2018.04.090

[R33] Zhan Z, Zhao Y, Pang S, Zhong X, Wu C, Ding Z, 2017. Temperature change between neighboring days and mortality in United States: A nationwide study. Sci. Total Environ 584–585, 1152–1161. 10.1016/j.scitotenv.2017.01.177 28162760

[R34] Zhang Y, Peng M, Wang L, Yu C, 2018. Association of diurnal temperature range with daily mortality in England and Wales: A nationwide time-series study. Sci. Total Environ 619–620, 291–300. 10.1016/j.scitotenv.2017.11.056 29154047

[R35] Zhang Y, Xiang Q, Yu C, Bao J, Ho HC, Sun S, Ding Z, Hu K, Zhang L, 2019. Mortality risk and burden associated with temperature variability in China, United Kingdom and United States: Comparative analysis of daily and hourly exposure metrics. Environ. Res 179, 108771. 31574448 10.1016/j.envres.2019.108771

[R36] Zhao Q, Coelho M, Li S, Saldiva PHN, Hu K, Abramson MJ, Huxley RR, Guo Y, 2018. Spatiotemporal and demographic variation in the association between temperature variability and hospitalizations in Brazil during 2000–2015: a nationwide time-series study. Environ. Int 120, 345–353. 30114624 10.1016/j.envint.2018.08.021

[R37] Zhao Q, Li S, Coelho MSZS, Saldiva PHN, Hu K, Huxley RR, Abramson MJ, Guo Y, 2019. Temperature variability and hospitalization for ischaemic heart disease in Brazil: A nationwide case-crossover study during 2000–2015. Sci. Total Environ 664, 707–712. 30763851 10.1016/j.scitotenv.2019.02.066

