# Supplementary material for: Comparison for the effects of different components of temperature variability on mortality: A multi-country time-series study
Source: Environ Int. Author manuscript; Available in PMC 2025 Aug 2. (PMC12317778; doi:10.1016/j.envint.2024.108712)
Supplement: Supplementary material [file NIHMS2098854-supplement-Supplementary_material.docx]

**Comparison for the effects of different components of temperature variability on mortality: a multi-country time-series study**

Table of content

[1. eMethods. 4](#_Toc134456110)

[1.1 Description of the data 4](#_Toc134456111)

[1.2 Relationship with pre-existing indices 5](#_Toc134456112)

[1.3 Calculation of attributable fraction (AF) 5](#_Toc134456113)

[2. Results 7](#_Toc134456114)

[Table S1. Summary of inter-day and intra-day TV_0–1_ to TV_0–6_ in 47 countries/regions 7](#_Toc134456115)

[Table S2. Results for test of heterogeneity across all cities 9](#_Toc134456116)

[Table S3. Percentage change (%) of all-cause mortality risk per IQR increase in TV. 10](#_Toc134456117)

[Table S4. Percentage change (%) of cardiovascular mortality risk per IQR increase in TV. 12](#_Toc134456118)

[Table S5. Percentage change (%) of respiratory mortality risk per IQR increase in TV. 14](#_Toc134456119)

[Table S6. Percentage change (%) of all-cause mortality risk by climate areas per IQR increase in TV. 16](#_Toc134456120)

[Table S7. Percentage change (%) of cardiovascular mortality risk by climate areas per IQR increase in TV. 17](#_Toc134456121)

[Table S8. Percentage change (%) of respiratory mortality risk by climate areas per IQR increase in TV. 18](#_Toc134456122)

[Table S9. Percentage change (%) of all-cause mortality risk by seasons per IQR increase in TV. 19](#_Toc134456123)

[Table S10. Percentage change (%) of cardiovascular mortality risk by seasons per IQR increase in TV. 20](#_Toc134456124)

[Table S11. Percentage change (%) of respiratory mortality risk by seasons per IQR increase in TV. 21](#_Toc134456125)

[Table S12. Percentage change (%) of all-cause mortality risk by Köppen-Geiger climate groups per IQR increase in TV. 22](#_Toc134456126)

[Table S13. Percentage change (%) of cardiovascular mortality risk by Köppen-Geiger climate groups per IQR increase in TV. 23](#_Toc134456127)

[Table S14. Percentage change (%) of respiratory mortality risk by Köppen-Geiger climate groups per IQR increase in TV. 24](#_Toc134456128)

[Table S15. Attributable fractions (%) of both inter-day TV and intra-day TV for all-cause mortality 25](#_Toc134456129)

[Table S16. Attributable fractions (%) of both inter-day TV and intra-day TV for cardiovascular mortality 27](#_Toc134456130)

[Table S17. Attributable fractions (%) of both inter-day TV and intra-day TV for respiratory mortality 29](#_Toc134456131)

[Table S18. Annual average attributable deaths of both inter-day TV and intra-day TV for all-cause mortality 31](#_Toc134456132)

[Table S19. Annual average attributable deaths of both inter-day TV and intra-day TV for cardiovascular mortality 33](#_Toc134456133)

[Table S20. Annual average attributable deaths of both inter-day TV and intra-day TV for respiratory mortality 35](#_Toc134456134)

[Figure S1. Sensitivity analyses of comparing effects of inter-day and intra-day TV per 1℃ increase in TV. 37](#_Toc134456135)

[Figure S2. Sensitivity analyses of comparing effects of inter-day and intra-day TV per 1℃ increase of TV in all countries/regions (A) and locations (B and C). 38](#_Toc134456136)

[Figure S3. Sensitivity analyses to change lag days for daily mean temperatures (21 to 28 days) 39](#_Toc134456137)

[Figure S4. Sensitivity analyses to change df for daily mean temperatures (3–6 df) 40](#_Toc134456138)

[Figure S5. Sensitivity analyses to change daily mean temperature to daily maximum or minimum temperature. T-max = daily maximum temperature, T-mean = daily mean temperature, T-min = daily minimum temperature. 41](#_Toc134456139)

[Figure S6. Sensitivity analyses to include only one of TV indicators (inter-day TV and intra-day TV) in the models. 42](#_Toc134456140)

[Figure S7. Sensitivity analyses to include relative humidity in the model. 43](#_Toc134456141)

# 1. eMethods.

## 1.1 Description of the data

A detailed description of the mortality and environmental data used in the present study was provided in this section. As mentioned before, Mortality data and environment data were obtained from the Multi-country Multi-city (MCC) Collaborative Research Network (https://mccstudy.lshtm.ac.uk/). Generally, we have examined associations between mortality and many environmental exposures in the previous studies, including temperature variability (TV),(Guoet al. 2016) ambient temperature,(Gasparriniet al. 2015) particulate matter,(Liuet al. 2019) ozone,(Vicedo-Cabreraet al. 2020) nitrogen dioxide(Menget al. 2021) and carbon monoxide (Chenet al. 2021). The current MCC network covers 760 locations from 47 countries or regions. For some countries, daily minimum temperatures and maximum temperatures (used to calculate inter-day and intra-day TV) were not available. Thus, we collected weather data from from the ECMWF Reanalysis 5th Generation (ERA5) land dataset at 0.5˚ × 0.5˚ spatial resolution (https://cds.climate.copernicus.eu/cdsapp#!/home) for these countries.

For locations with daily minimum temperatures and maximum temperatures, we also checked the missing values and replaced all the observations of weather data when the missing rate was over 80% using the ERA5 dataset. For locations with a missing rate lower than 80%, we replaced the missing values on each location using the ERA5 dataset. For mortality data, two types of missing values were identified: missing at random and missing not at random. For the first type, missing values only could be observed on random days during the study period. For the second type, mortality data could be missing for several entire months in specific year on some locations. In that case, we excluded relative years containing non-random missing values on these locations. Finally, 758 locations included in this study all have a coverage over 2 years. The overall missing rate for all-cause mortality, cardiovascular mortality, respiratory mortality, and minimum/maximum temperature was 0.001%, 0.08%, 0.14%, and 0.00014%, respectively.

## 1.2 Relationship with pre-existing indices

Diurnal temperature range (DTR) and temperature change between the neighbouring days (TCN)were previously used as indicators for intra-day and inter-day temperature (Huet al. 2021; Vicedo-Cabreraet al. 2016). Our newly defined intra-day and inter-day TV could be good proxy for DTR and TCN. For intra-day TV and DTR, we could give the relationship with the following equation:

$${TV}_{intra-day,0-L}=\sqrt{\frac{\sum\left[ \left( T_{l,min}-\bar{T_{l}} \right)^{2}+\left( T_{l,max}-\bar{T_{l}} \right)^{2} \right]}{2L+1}}=\sqrt{\frac{\sum\left( T_{l,max}-T_{l,min} \right)^{2}}{2(2L+1)}}=\sqrt{\frac{\sum({DTR}_{l}^{2})}{2(2L+1)}}$$

For inter-day TV_0–1_ and TCN, their relationship could be expressed as:

$$\mathrm{TV}_{inter-day, 0-1}=\frac{TCN}{\sqrt{3}}$$

It would be difficult to express the relationship between inter-day TV and TCN. Nevertheless, the decomposition made the percentage change in mortality risk and attributable fraction of the two indices comparable.

## 1.3 Calculation of attributable fraction (AF)

We calculated the attributable fractions (AF) using the following equations:

$${RR}_{i,t}=exp(\beta\times({TV}_{i,t}-{TV}_{i,ref}))$$

$${AN}_{i,t}={Number of deaths}_{i,t}\times({RR}_{i,t}-1)/{RR}_{i,t}$$

$$AN=\sum_{i} \sum_{t} {AN}_{i,t}$$

$$AF=\frac{AN}{Total number of deaths}$$

where $\beta$ is the pooled effect estimates associated with per 1 ℃ increase of inter-day TV or intra-day TV, ${TV}_{i,t}$ is the inter-day TV or intra-day TV for city *i* on day *t*, ${TV}_{i,ref}$ is the minimum inter-day TV or intra-day TV (defined as the reference TV or the counterfactual scenario), ${RR}_{i,t}$ is the cumulative relative risk of inter-day or intra-day TV in city *i* on day *t*, ${AN}_{i,t}$ is the attributable number of deaths for city *i* on day *t*, and $AF$ is the attributable fraction calculated at the global level. The AFs at the country level were calculated using the same method. Additionally, 95% confidence interval (CI) was calculated using the 95% CI of pooled effect estimates.

**References:**

Chen, K.; Breitner, S.; Wolf, K.; Stafoggia, M.; Sera, F.; Vicedo-Cabrera, A.M.; Guo, Y.; Tong, S.; Lavigne, E.; Matus, P.; Valdes, N.; Kan, H.; Jaakkola, J.J.K.; Ryti, N.R.I.; Huber, V.; Scortichini, M.; Hashizume, M.; Honda, Y.; Nunes, B.; Madureira, J.; Holobaca, I.H.; Fratianni, S.; Kim, H.; Lee, W.; Tobias, A.; Iniguez, C.; Forsberg, B.; Astrom, C.; Ragettli, M.S.; Guo, Y.L.; Chen, B.Y.; Li, S.; Milojevic, A.; Zanobetti, A.; Schwartz, J.; Bell, M.L.; Gasparrini, A.; Schneider, A. Ambient carbon monoxide and daily mortality: a global time-series study in 337 cities. Lancet Planet Health 2021;5:e191-e199

Gasparrini, A.; Guo, Y.; Hashizume, M.; Lavigne, E.; Zanobetti, A.; Schwartz, J.; Tobias, A.; Tong, S.; Rocklov, J.; Forsberg, B.; Leone, M.; De Sario, M.; Bell, M.L.; Guo, Y.L.; Wu, C.F.; Kan, H.; Yi, S.M.; de Sousa Zanotti Stagliorio Coelho, M.; Saldiva, P.H.; Honda, Y.; Kim, H.; Armstrong, B. Mortality risk attributable to high and low ambient temperature: a multicountry observational study. Lancet 2015;386:369-375

Guo, Y.; Gasparrini, A.; Armstrong, B.G.; Tawatsupa, B.; Tobias, A.; Lavigne, E.; Coelho, M.S.; Pan, X.; Kim, H.; Hashizume, M.; Honda, Y.; Guo, Y.L.; Wu, C.F.; Zanobetti, A.; Schwartz, J.D.; Bell, M.L.; Overcenco, A.; Punnasiri, K.; Li, S.; Tian, L.; Saldiva, P.; Williams, G.; Tong, S. Temperature Variability and Mortality: A Multi-Country Study. Environmental health perspectives 2016;124:1554-1559

Hu, Y.; Cheng, J.; Yin, Y.; Liu, S.; Tan, J.; Li, S.; Wu, M.; Yan, C.; Yu, G.; Hu, Y.; Tong, S. Association of childhood asthma with intra-day and inter-day temperature variability in Shanghai, China. Environmental research 2021:112350

Liu, C.; Chen, R.; Sera, F.; Vicedo-Cabrera, A.M.; Guo, Y.; Tong, S.; Coelho, M.; Saldiva, P.H.N.; Lavigne, E.; Matus, P.; Valdes Ortega, N.; Osorio Garcia, S.; Pascal, M.; Stafoggia, M.; Scortichini, M.; Hashizume, M.; Honda, Y.; Hurtado-Diaz, M.; Cruz, J.; Nunes, B.; Teixeira, J.P.; Kim, H.; Tobias, A.; Iniguez, C.; Forsberg, B.; Astrom, C.; Ragettli, M.S.; Guo, Y.L.; Chen, B.Y.; Bell, M.L.; Wright, C.Y.; Scovronick, N.; Garland, R.M.; Milojevic, A.; Kysely, J.; Urban, A.; Orru, H.; Indermitte, E.; Jaakkola, J.J.K.; Ryti, N.R.I.; Katsouyanni, K.; Analitis, A.; Zanobetti, A.; Schwartz, J.; Chen, J.; Wu, T.; Cohen, A.; Gasparrini, A.; Kan, H. Ambient Particulate Air Pollution and Daily Mortality in 652 Cities. The New England journal of medicine 2019;381:705-715

Meng, X.; Liu, C.; Chen, R.; Sera, F.; Vicedo-Cabrera, A.M.; Milojevic, A.; Guo, Y.; Tong, S.; Coelho, M.; Saldiva, P.H.N.; Lavigne, E.; Correa, P.M.; Ortega, N.V.; Osorio, S.; Garcia; Kysely, J.; Urban, A.; Orru, H.; Maasikmets, M.; Jaakkola, J.J.K.; Ryti, N.; Huber, V.; Schneider, A.; Katsouyanni, K.; Analitis, A.; Hashizume, M.; Honda, Y.; Ng, C.F.S.; Nunes, B.; Teixeira, J.P.; Holobaca, I.H.; Fratianni, S.; Kim, H.; Tobias, A.; Iniguez, C.; Forsberg, B.; Astrom, C.; Ragettli, M.S.; Guo, Y.L.; Pan, S.C.; Li, S.; Bell, M.L.; Zanobetti, A.; Schwartz, J.; Wu, T.; Gasparrini, A.; Kan, H. Short term associations of ambient nitrogen dioxide with daily total, cardiovascular, and respiratory mortality: multilocation analysis in 398 cities. BMJ (Clinical research ed) 2021;372:n534

Vicedo-Cabrera, A.M.; Forsberg, B.; Tobias, A.; Zanobetti, A.; Schwartz, J.; Armstrong, B.; Gasparrini, A. Associations of Inter- and Intraday Temperature Change With Mortality. American journal of epidemiology 2016;183:286-293

Vicedo-Cabrera, A.M.; Sera, F.; Liu, C.; Armstrong, B.; Milojevic, A.; Guo, Y.; Tong, S.; Lavigne, E.; Kysely, J.; Urban, A.; Orru, H.; Indermitte, E.; Pascal, M.; Huber, V.; Schneider, A.; Katsouyanni, K.; Samoli, E.; Stafoggia, M.; Scortichini, M.; Hashizume, M.; Honda, Y.; Ng, C.F.S.; Hurtado-Diaz, M.; Cruz, J.; Silva, S.; Madureira, J.; Scovronick, N.; Garland, R.M.; Kim, H.; Tobias, A.; Iniguez, C.; Forsberg, B.; Astrom, C.; Ragettli, M.S.; Roosli, M.; Guo, Y.L.; Chen, B.Y.; Zanobetti, A.; Schwartz, J.; Bell, M.L.; Kan, H.; Gasparrini, A. Short term association between ozone and mortality: global two stage time series study in 406 locations in 20 countries. BMJ (Clinical research ed) 2020;368:m108

# 2. Results

## Table S1. Summary of inter-day and intra-day TV_0–1_ to TV_0–6_ in 47 countries/regions

| Country/region | TV 0–1, Median (IQR) | | TV 0–2, Median (IQR) | | TV 0–3, Median (IQR) | | TV 0–4, Median (IQR) | | TV 0–5, Median (IQR) | | TV 0–6, Median (IQR) | |
| --- | --- | --- | --- | --- | --- | --- | --- | --- | --- | --- | --- | --- |
|  | Inter-day | Intra-day | Inter-day | Intra-day | Inter-day | Intra-day | Inter-day | Intra-day | Inter-day | Intra-day | Inter-day | Intra-day |
| Argentina | 1.2 (1.2, 1.3) | 7.0 (5.6, 7.4) | 1.7 (1.6, 1.8) | 6.7 (5.4, 7.0) | 2.0 (1.9, 2.1) | 6.5 (5.3, 6.9) | 2.2 (2.1, 2.3) | 6.4 (5.2, 6.8) | 2.4 (2.2, 2.5) | 6.4 (5.2, 6.8) | 2.5 (2.4, 2.6) | 6.4 (5.1, 6.8) |
| Australia | 0.9 (0.7, 1.2) | 4.9 (4.6, 5.1) | 1.2 (1.0, 1.7) | 4.7 (4.4, 4.9) | 1.4 (1.1, 1.9) | 4.6 (4.3, 4.8) | 1.5 (1.3, 2.1) | 4.5 (4.2, 4.8) | 1.6 (1.3, 2.2) | 4.5 (4.2, 4.7) | 1.7 (1.4, 2.3) | 4.5 (4.2, 4.7) |
| Brazil | 0.6 (0.5, 0.8) | 5.7 (4.7, 6.4) | 0.8 (0.6, 1.1) | 5.4 (4.5, 6.1) | 1.0 (0.7, 1.3) | 5.3 (4.4, 6.0) | 1.1 (0.8, 1.5) | 5.3 (4.3, 5.9) | 1.1 (0.8, 1.6) | 5.3 (4.3, 5.9) | 1.2 (0.9, 1.7) | 5.2 (4.2, 5.8) |
| Canada | 1.6 (1.4, 1.7) | 5.5 (4.9, 6.1) | 2.2 (2.0, 2.3) | 5.3 (4.7, 5.9) | 2.5 (2.3, 2.7) | 5.2 (4.6, 5.8) | 2.8 (2.5, 3.0) | 5.1 (4.6, 5.7) | 2.9 (2.7, 3.2) | 5.1 (4.5, 5.7) | 3.1 (2.8, 3.3) | 5.1 (4.5, 5.7) |
| Chile | 0.7 (0.5, 0.8) | 6.0 (3.7, 6.9) | 1.0 (0.6, 1.1) | 5.8 (3.5, 6.6) | 1.2 (0.7, 1.3) | 5.7 (3.4, 6.5) | 1.3 (0.8, 1.4) | 5.6 (3.4, 6.4) | 1.4 (0.9, 1.5) | 5.6 (3.4, 6.4) | 1.4 (0.9, 1.6) | 5.6 (3.4, 6.3) |
| China | 0.9 (0.8, 1.0) | 5.5 (3.7, 6.3) | 1.3 (1.2, 1.4) | 5.3 (3.6, 6.0) | 1.5 (1.4, 1.7) | 5.2 (3.5, 5.9) | 1.7 (1.5, 1.9) | 5.2 (3.5, 5.8) | 1.8 (1.6, 2.0) | 5.1 (3.4, 5.8) | 1.9 (1.8, 2.1) | 5.1 (3.4, 5.7) |
| Colombia | 0.5 (0.4, 0.5) | 5.6 (4.2, 6.1) | 0.6 (0.6, 0.7) | 5.3 (4.0, 5.8) | 0.7 (0.6, 0.8) | 5.2 (4.0, 5.6) | 0.8 (0.7, 0.8) | 5.1 (3.9, 5.5) | 0.8 (0.7, 0.8) | 5.1 (3.9, 5.5) | 0.8 (0.7, 0.9) | 5.0 (3.9, 5.5) |
| Costa Rica | 0.5 (0.4, 0.5) | 5.0 (4.9, 5.1) | 0.6 (0.6, 0.6) | 4.8 (4.7, 4.8) | 0.7 (0.6, 0.7) | 4.7 (4.6, 4.7) | 0.7 (0.7, 0.7) | 4.6 (4.5, 4.7) | 0.7 (0.7, 0.7) | 4.6 (4.5, 4.6) | 0.7 (0.7, 0.8) | 4.5 (4.5, 4.6) |
| Czech Republic | 1.2 (1.1, 1.2) | 3.4 (3.2, 3.5) | 1.6 (1.6, 1.7) | 3.3 (3.1, 3.4) | 1.9 (1.9, 2.0) | 3.2 (3.0, 3.3) | 2.2 (2.1, 2.2) | 3.2 (3.0, 3.3) | 2.3 (2.2, 2.4) | 3.2 (3.0, 3.3) | 2.5 (2.4, 2.6) | 3.2 (3.0, 3.3) |
| Ecuador | 0.6 (0.5, 0.6) | 5.4 (4.1, 6.8) | 0.8 (0.6, 0.8) | 5.1 (3.9, 6.5) | 0.8 (0.7, 0.9) | 5.0 (3.8, 6.4) | 0.9 (0.7, 1.0) | 4.9 (3.8, 6.3) | 0.9 (0.7, 1.0) | 4.9 (3.7, 6.2) | 0.9 (0.8, 1.0) | 4.9 (3.7, 6.2) |
| Estonia | 1.1 (1.0, 1.2) | 4.6 (4.2, 4.8) | 1.5 (1.4, 1.6) | 4.4 (4.1, 4.6) | 1.8 (1.7, 1.9) | 4.3 (4.0, 4.5) | 2.0 (1.9, 2.1) | 4.3 (4.0, 4.5) | 2.1 (2.0, 2.3) | 4.3 (4.0, 4.5) | 2.3 (2.2, 2.5) | 4.2 (3.9, 4.4) |
| Finland | 1.0 (1.0, 1.1) | 3.7 (3.6, 3.9) | 1.4 (1.3, 1.5) | 3.6 (3.5, 3.7) | 1.7 (1.6, 1.8) | 3.5 (3.5, 3.7) | 1.8 (1.8, 2.0) | 3.5 (3.4, 3.6) | 2.0 (1.9, 2.1) | 3.5 (3.4, 3.6) | 2.1 (2.0, 2.2) | 3.5 (3.4, 3.6) |
| France | 1.0 (0.9, 1.0) | 5.2 (4.5, 5.5) | 1.4 (1.2, 1.4) | 5.0 (4.3, 5.3) | 1.6 (1.5, 1.7) | 4.9 (4.2, 5.2) | 1.8 (1.6, 1.9) | 4.9 (4.2, 5.2) | 1.9 (1.7, 2.0) | 4.8 (4.1, 5.1) | 2.0 (1.8, 2.2) | 4.8 (4.1, 5.1) |
| French Caribbean | 0.4 (0.3, 0.4) | 4.2 (3.9, 4.3) | 0.5 (0.5, 0.5) | 4.0 (3.7, 4.1) | 0.5 (0.5, 0.5) | 3.9 (3.6, 4.0) | 0.6 (0.5, 0.6) | 3.8 (3.6, 4.0) | 0.6 (0.6, 0.6) | 3.8 (3.6, 3.9) | 0.6 (0.6, 0.6) | 3.8 (3.5, 3.9) |
| French Guiana | 0.4 (0.3, 0.4) | 4.5 (4.4, 4.6) | 0.4 (0.4, 0.5) | 4.3 (4.2, 4.4) | 0.5 (0.5, 0.5) | 4.2 (4.1, 4.3) | 0.5 (0.5, 0.5) | 4.1 (4.1, 4.2) | 0.5 (0.5, 0.6) | 4.1 (4.0, 4.2) | 0.5 (0.5, 0.6) | 4.1 (4.0, 4.2) |
| Réunion | 0.4 (0.4, 0.4) | 3.9 (3.7, 4.0) | 0.5 (0.5, 0.5) | 3.7 (3.5, 3.8) | 0.6 (0.5, 0.6) | 3.6 (3.5, 3.7) | 0.6 (0.6, 0.6) | 3.6 (3.4, 3.7) | 0.6 (0.6, 0.7) | 3.5 (3.4, 3.7) | 0.7 (0.6, 0.7) | 3.5 (3.4, 3.6) |
| Germany | 1.0 (1.0, 1.1) | 4.9 (4.6, 5.1) | 1.5 (1.4, 1.5) | 4.7 (4.4, 4.9) | 1.7 (1.7, 1.8) | 4.6 (4.4, 4.8) | 1.9 (1.9, 2.0) | 4.5 (4.3, 4.8) | 2.1 (2.0, 2.2) | 4.5 (4.3, 4.7) | 2.2 (2.1, 2.3) | 4.5 (4.3, 4.7) |
| Greece | 0.7 (0.7, 0.8) | 4.5 (4.4, 4.7) | 1.1 (1.0, 1.1) | 4.3 (4.2, 4.5) | 1.3 (1.2, 1.3) | 4.2 (4.1, 4.4) | 1.4 (1.4, 1.5) | 4.2 (4.1, 4.4) | 1.6 (1.5, 1.6) | 4.2 (4.0, 4.3) | 1.7 (1.6, 1.7) | 4.1 (4.0, 4.3) |
| Guatemala | 0.5 (0.5, 0.5) | 5.3 (5.1, 5.3) | 0.7 (0.6, 0.7) | 5.0 (4.9, 5.1) | 0.7 (0.7, 0.8) | 4.9 (4.8, 5.0) | 0.8 (0.8, 0.9) | 4.9 (4.7, 4.9) | 0.9 (0.8, 0.9) | 4.8 (4.7, 4.9) | 0.9 (0.9, 0.9) | 4.8 (4.7, 4.8) |
| Iran | 0.9 (0.9, 1.0) | 6.1 (5.7, 7.4) | 1.3 (1.3, 1.4) | 5.8 (5.4, 7.1) | 1.6 (1.5, 1.7) | 5.7 (5.3, 6.9) | 1.8 (1.7, 1.9) | 5.6 (5.3, 6.8) | 1.9 (1.9, 2.1) | 5.6 (5.2, 6.8) | 2.1 (2.0, 2.2) | 5.5 (5.2, 6.8) |
| Ireland | 0.8 (0.7, 0.9) | 3.9 (3.5, 4.3) | 1.1 (1.0, 1.2) | 3.8 (3.4, 4.1) | 1.3 (1.1, 1.4) | 3.7 (3.3, 4.0) | 1.4 (1.3, 1.5) | 3.7 (3.3, 4.0) | 1.5 (1.3, 1.6) | 3.6 (3.2, 4.0) | 1.5 (1.4, 1.7) | 3.6 (3.2, 4.0) |
| Israel | 0.7 (0.7, 0.7) | 6.3 (6.1, 6.4) | 0.9 (0.9, 1.0) | 6.0 (5.8, 6.1) | 1.1 (1.0, 1.2) | 5.9 (5.7, 6.0) | 1.2 (1.1, 1.3) | 5.8 (5.6, 6.0) | 1.3 (1.2, 1.3) | 5.8 (5.6, 5.9) | 1.3 (1.3, 1.4) | 5.8 (5.6, 5.9) |
| Italy | 0.7 (0.6, 0.7) | 4.8 (4.0, 5.4) | 0.9 (0.9, 1.0) | 4.6 (3.8, 5.1) | 1.1 (1.0, 1.2) | 4.5 (3.7, 5.0) | 1.2 (1.2, 1.3) | 4.5 (3.7, 5.0) | 1.3 (1.3, 1.4) | 4.4 (3.7, 4.9) | 1.4 (1.3, 1.5) | 4.4 (3.7, 4.9) |
| Japan | 0.9 (0.8, 1.0) | 5.0 (4.5, 5.4) | 1.2 (1.1, 1.3) | 4.8 (4.3, 5.2) | 1.4 (1.3, 1.5) | 4.7 (4.2, 5.1) | 1.6 (1.5, 1.7) | 4.6 (4.2, 5.1) | 1.7 (1.6, 1.8) | 4.6 (4.2, 5.0) | 1.7 (1.6, 1.9) | 4.6 (4.1, 5.0) |
| Kuwait | 0.6 (0.6, 0.6) | 4.8 (4.7, 4.9) | 0.8 (0.8, 0.8) | 4.5 (4.5, 4.6) | 1.0 (1.0, 1.0) | 4.4 (4.4, 4.5) | 1.1 (1.1, 1.1) | 4.4 (4.3, 4.5) | 1.2 (1.1, 1.2) | 4.3 (4.3, 4.5) | 1.2 (1.2, 1.2) | 4.3 (4.3, 4.4) |
| Mexico | 0.7 (0.5, 0.9) | 7.7 (6.8, 8.3) | 0.9 (0.7, 1.3) | 7.4 (6.5, 7.9) | 1.1 (0.8, 1.5) | 7.2 (6.4, 7.8) | 1.2 (0.9, 1.7) | 7.1 (6.3, 7.7) | 1.2 (0.9, 1.8) | 7.1 (6.2, 7.6) | 1.3 (1.0, 1.8) | 7.0 (6.2, 7.6) |
| Moldova | 1.1 (1.1, 1.2) | 5.7 (5.1, 6.1) | 1.6 (1.5, 1.6) | 5.4 (4.9, 5.9) | 1.8 (1.8, 2.0) | 5.3 (4.8, 5.8) | 2.1 (2.0, 2.2) | 5.2 (4.7, 5.7) | 2.2 (2.2, 2.4) | 5.2 (4.7, 5.7) | 2.4 (2.3, 2.5) | 5.2 (4.7, 5.7) |
| Netherland | 1.0 (0.9, 1.0) | 4.6 (4.3, 4.9) | 1.3 (1.3, 1.4) | 4.4 (4.1, 4.7) | 1.6 (1.5, 1.6) | 4.3 (4.0, 4.6) | 1.7 (1.7, 1.8) | 4.3 (4.0, 4.6) | 1.9 (1.8, 2.0) | 4.2 (4.0, 4.5) | 2.0 (1.9, 2.1) | 4.2 (4.0, 4.5) |
| Norway | 1.0 (1.0, 1.1) | 4.1 (4.0, 4.3) | 1.4 (1.3, 1.4) | 4.0 (3.9, 4.1) | 1.6 (1.6, 1.7) | 3.9 (3.8, 4.0) | 1.8 (1.7, 1.9) | 3.9 (3.8, 4.0) | 2.0 (1.9, 2.1) | 3.9 (3.8, 4.0) | 2.1 (2.0, 2.2) | 3.8 (3.8, 4.0) |
| Panama | 0.5 (0.4, 0.5) | 4.4 (4.4, 4.5) | 0.6 (0.6, 0.6) | 4.2 (4.2, 4.3) | 0.7 (0.7, 0.7) | 4.1 (4.1, 4.2) | 0.7 (0.7, 0.8) | 4.1 (4.0, 4.2) | 0.8 (0.7, 0.8) | 4.0 (4.0, 4.1) | 0.8 (0.7, 0.8) | 4.0 (4.0, 4.1) |
| Paraguay | 1.2 (1.2, 1.3) | 6.2 (6.1, 6.3) | 1.7 (1.7, 1.8) | 5.9 (5.8, 6.0) | 2.1 (2.0, 2.2) | 5.8 (5.7, 5.9) | 2.3 (2.3, 2.4) | 5.8 (5.7, 5.8) | 2.5 (2.4, 2.6) | 5.7 (5.6, 5.8) | 2.6 (2.6, 2.7) | 5.7 (5.6, 5.7) |
| Peru | 0.5 (0.4, 0.6) | 6.9 (5.7, 8.0) | 0.6 (0.6, 0.8) | 6.6 (5.4, 7.6) | 0.7 (0.6, 0.9) | 6.4 (5.3, 7.4) | 0.8 (0.7, 0.9) | 6.3 (5.2, 7.3) | 0.8 (0.7, 0.9) | 6.3 (5.2, 7.3) | 0.8 (0.7, 1.0) | 6.2 (5.2, 7.2) |
| Philippines | 0.3 (0.3, 0.3) | 3.1 (2.9, 3.2) | 0.4 (0.4, 0.4) | 2.9 (2.8, 3.0) | 0.5 (0.4, 0.5) | 2.8 (2.7, 3.0) | 0.5 (0.5, 0.6) | 2.8 (2.7, 3.0) | 0.6 (0.5, 0.6) | 2.8 (2.7, 2.9) | 0.6 (0.6, 0.6) | 2.8 (2.7, 2.9) |
| Portugal | 0.8 (0.8, 0.9) | 5.5 (5.0, 6.2) | 1.1 (1.1, 1.2) | 5.2 (4.8, 6.0) | 1.3 (1.3, 1.4) | 5.1 (4.7, 5.8) | 1.5 (1.4, 1.6) | 5.1 (4.7, 5.8) | 1.6 (1.5, 1.7) | 5.1 (4.6, 5.7) | 1.7 (1.6, 1.8) | 5.0 (4.6, 5.7) |
| Puerto Rico | 0.4 (0.4, 0.4) | 3.9 (3.8, 4.0) | 0.5 (0.5, 0.5) | 3.7 (3.6, 3.8) | 0.6 (0.5, 0.6) | 3.6 (3.5, 3.7) | 0.6 (0.6, 0.6) | 3.6 (3.5, 3.7) | 0.6 (0.6, 0.6) | 3.6 (3.5, 3.7) | 0.7 (0.6, 0.7) | 3.5 (3.5, 3.6) |
| Romania | 1.0 (1.0, 1.1) | 6.1 (5.7, 6.6) | 1.4 (1.4, 1.5) | 5.8 (5.4, 6.4) | 1.7 (1.6, 1.8) | 5.7 (5.3, 6.2) | 1.9 (1.8, 2.0) | 5.7 (5.2, 6.2) | 2.1 (2.0, 2.2) | 5.6 (5.2, 6.1) | 2.2 (2.1, 2.4) | 5.6 (5.2, 6.1) |
| South Africa | 1.0 (0.8, 1.2) | 8.2 (7.4, 9.2) | 1.4 (1.2, 1.7) | 7.8 (7.1, 8.8) | 1.6 (1.4, 1.9) | 7.7 (6.9, 8.6) | 1.7 (1.5, 2.1) | 7.6 (6.9, 8.5) | 1.8 (1.6, 2.2) | 7.5 (6.8, 8.5) | 1.9 (1.7, 2.3) | 7.5 (6.8, 8.4) |
| South Korea | 1.0 (0.9, 1.0) | 5.8 (4.8, 6.6) | 1.4 (1.3, 1.4) | 5.6 (4.6, 6.4) | 1.6 (1.5, 1.7) | 5.5 (4.5, 6.2) | 1.7 (1.6, 1.9) | 5.4 (4.4, 6.2) | 1.9 (1.7, 2.0) | 5.4 (4.4, 6.1) | 2.0 (1.8, 2.1) | 5.3 (4.4, 6.1) |
| Spain | 0.8 (0.6, 0.8) | 5.6 (3.9, 6.3) | 1.1 (0.9, 1.2) | 5.4 (3.7, 6.0) | 1.3 (1.0, 1.4) | 5.3 (3.7, 5.9) | 1.4 (1.2, 1.6) | 5.2 (3.6, 5.8) | 1.5 (1.2, 1.7) | 5.2 (3.6, 5.7) | 1.6 (1.3, 1.9) | 5.1 (3.6, 5.7) |
| Sweden | 0.9 (0.8, 1.0) | 3.9 (3.7, 4.1) | 1.2 (1.2, 1.3) | 3.7 (3.5, 3.9) | 1.5 (1.4, 1.5) | 3.6 (3.4, 3.8) | 1.6 (1.5, 1.7) | 3.6 (3.4, 3.8) | 1.7 (1.6, 1.8) | 3.6 (3.4, 3.8) | 1.8 (1.7, 2.0) | 3.6 (3.4, 3.8) |
| Switzerland | 0.9 (0.9, 1.0) | 4.5 (4.0, 4.9) | 1.3 (1.2, 1.4) | 4.3 (3.8, 4.7) | 1.6 (1.5, 1.7) | 4.2 (3.8, 4.6) | 1.8 (1.7, 1.9) | 4.2 (3.7, 4.6) | 1.9 (1.8, 2.1) | 4.1 (3.7, 4.6) | 2.1 (1.9, 2.2) | 4.1 (3.7, 4.5) |
| Taiwan | 0.7 (0.6, 0.8) | 4.0 (3.7, 4.8) | 0.9 (0.8, 1.2) | 3.9 (3.6, 4.6) | 1.1 (1.0, 1.4) | 3.8 (3.5, 4.5) | 1.2 (1.1, 1.5) | 3.7 (3.5, 4.4) | 1.3 (1.2, 1.6) | 3.7 (3.5, 4.4) | 1.4 (1.3, 1.7) | 3.7 (3.5, 4.4) |
| Thailand | 0.4 (0.4, 0.5) | 5.7 (5.1, 6.2) | 0.6 (0.5, 0.7) | 5.5 (4.9, 5.9) | 0.7 (0.6, 0.8) | 5.3 (4.8, 5.7) | 0.8 (0.6, 0.9) | 5.3 (4.7, 5.7) | 0.8 (0.7, 0.9) | 5.2 (4.7, 5.6) | 0.9 (0.7, 1.0) | 5.2 (4.7, 5.6) |
| UK | 0.8 (0.8, 0.9) | 3.6 (3.0, 3.8) | 1.2 (1.0, 1.2) | 3.4 (2.9, 3.7) | 1.3 (1.2, 1.4) | 3.4 (2.9, 3.6) | 1.5 (1.3, 1.6) | 3.3 (2.8, 3.6) | 1.6 (1.4, 1.7) | 3.3 (2.8, 3.5) | 1.7 (1.5, 1.8) | 3.3 (2.8, 3.5) |
| Uruguay | 1.1 (1.1, 1.2) | 4.3 (4.3, 4.5) | 1.5 (1.5, 1.6) | 4.2 (4.2, 4.4) | 1.8 (1.7, 1.8) | 4.1 (4.1, 4.3) | 2.0 (1.9, 2.0) | 4.1 (4.1, 4.3) | 2.1 (2.0, 2.1) | 4.1 (4.1, 4.3) | 2.2 (2.2, 2.2) | 4.1 (4.1, 4.3) |
| USA | 1.3 (1.0, 1.5) | 5.5 (5.0, 6.1) | 1.8 (1.4, 2.1) | 5.3 (4.7, 5.8) | 2.1 (1.7, 2.4) | 5.2 (4.7, 5.7) | 2.4 (1.9, 2.7) | 5.2 (4.6, 5.7) | 2.5 (2.0, 2.9) | 5.1 (4.6, 5.6) | 2.7 (2.1, 3.0) | 5.1 (4.6, 5.6) |
| Vietnam | 0.6 (0.4, 0.6) | 4.9 (4.8, 4.9) | 0.8 (0.5, 0.9) | 4.6 (4.6, 4.7) | 1.0 (0.6, 1.0) | 4.5 (4.5, 4.6) | 1.1 (0.7, 1.2) | 4.5 (4.4, 4.5) | 1.2 (0.7, 1.3) | 4.4 (4.4, 4.5) | 1.3 (0.7, 1.3) | 4.4 (4.3, 4.5) |
| Pooled | 0.9 (0.8, 1.2) | 5.3 (4.2, 6.0) | 1.3 (1.1, 1.7) | 5.1 (4.0, 5.8) | 1.5 (1.3, 2.0) | 5.0 (4.0, 5.7) | 1.7 (1.4, 2.2) | 4.9 (3.9, 5.6) | 1.8 (1.5, 2.4) | 4.9 (3.9, 5.6) | 1.9 (1.6, 2.5) | 4.9 (3.9, 5.5) |

## Table S2. The proportion of zero death counts in 47 countries/regions

| Country/region | Proportion (%) |
| --- | --- |
| Argentina | 0.3 |
| Australia | 0.2 |
| Brazil | 2.7 |
| Canada | 0.9 |
| Chile | 1.1 |
| China | 0.5 |
| Colombia | 0 |
| Costa Rica | 1.3 |
| Czech Republic | 0 |
| Ecuador | 0 |
| Estonia | 10.2 |
| Finland | 0 |
| France | 0.2 |
| French Caribbean | 2.3 |
| French Guiana | 30.9 |
| Réunion | 10.3 |
| Germany | 0 |
| Greece | 0 |
| Guatemala | 0 |
| Iran | 0.9 |
| Ireland | 0 |
| Israel | 0 |
| Italy | 11.6 |
| Japan | 0 |
| Kuwait | 0.1 |
| Mexico | 0.1 |
| Moldova | 41.4 |
| Netherland | 0.2 |
| Norway | 1 |
| Panama | 0.1 |
| Paraguay | 0 |
| Peru | 2 |
| Philippines | 0 |
| Portugal | 0 |
| Puerto Rico | 0 |
| Romania | 0.1 |
| South Africa | 0.5 |
| South Korea | 5.3 |
| Spain | 10.4 |
| Sweden | 0 |
| Switzerland | 4.8 |
| Taiwan | 0 |
| Thailand | 1.8 |
| UK | 6.7 |
| Uruguay | 2.5 |
| USA | 0 |
| Vietnam | 3.2 |
| Pooled | 3.1 |

## Table S3. Results for test of heterogeneity across all locations

|  | All-cause mortality | | Cardiovascular mortality | | Respiratory mortality | |
| --- | --- | --- | --- | --- | --- | --- |
|  | I^2^ (%) | Cochran *P-*value | I^2^ (%) | Cochran *P-*value | I^2^ (%) | Cochran *P-*value |
| TV 0–1 | 18.1 | <0.01 | 0.0 | 0.92 | 5.3 | 0.15 |
| TV 0–2 | 10.9 | 0.01 | 3.5 | 0.25 | 4.7 | 0.18 |
| TV 0–3 | 11.0 | 0.01 | 0.8 | 0.43 | 9.9 | 0.02 |
| TV 0–4 | 19.9 | <0.01 | 10.2 | 0.02 | 10.7 | 0.02 |
| TV 0–5 | 22.8 | <0.01 | 10.2 | 0.02 | 15.3 | <0.01 |
| TV 0–6 | 24.7 | <0.01 | 15.5 | <0.01 | 13.7 | <0.01 |
| TV 0–7 | 25.8 | <0.01 | 15.6 | <0.01 | 8.3 | 0.05 |

## Table S4. Pooled percentage change (%) of all-cause, cardiovascular, and respiratory mortality risk per IQR increase in TV.

| Lag | TV | Percentage change (%, 95%CI) | *p* for difference |
| --- | --- | --- | --- |
| All-cause |  |  |  |
| TV 0-1 | Inter-day | -0.02 (-0.05, 0.01) | <0.001 |
| TV 0-1 | Intra-day | 0.57 (0.52, 0.63) | Ref |
| TV 0-2 | Inter-day | -0.04 (-0.07, -0.01) | <0.001 |
| TV 0-2 | Intra-day | 0.59 (0.54, 0.63) | Ref |
| TV 0-3 | Inter-day | 0.08 (0.05, 0.11) | <0.001 |
| TV 0-3 | Intra-day | 0.53 (0.48, 0.58) | Ref |
| TV 0-4 | Inter-day | 0.18 (0.14, 0.21) | <0.001 |
| TV 0-4 | Intra-day | 0.50 (0.45, 0.55) | Ref |
| TV 0-5 | Inter-day | 0.22 (0.18, 0.26) | <0.001 |
| TV 0-5 | Intra-day | 0.51 (0.45, 0.56) | Ref |
| TV 0-6 | Inter-day | 0.22 (0.18, 0.26) | <0.001 |
| TV 0-6 | Intra-day | 0.54 (0.48, 0.60) | Ref |
| TV 0-7 | Inter-day | 0.22 (0.18, 0.26) | <0.001 |
| TV 0-7 | Intra-day | 0.59 (0.53, 0.65) | Ref |
| Cardiovascular |  |  |  |
| TV 0-1 | Inter-day | 0.09 (0.04, 0.13) | <0.001 |
| TV 0-1 | Intra-day | 0.52 (0.45, 0.59) | Ref |
| TV 0-2 | Inter-day | 0.10 (0.05, 0.15) | <0.001 |
| TV 0-2 | Intra-day | 0.62 (0.55, 0.68) | Ref |
| TV 0-3 | Inter-day | 0.22 (0.16, 0.27) | <0.001 |
| TV 0-3 | Intra-day | 0.62 (0.55, 0.70) | Ref |
| TV 0-4 | Inter-day | 0.32 (0.26, 0.38) | <0.001 |
| TV 0-4 | Intra-day | 0.61 (0.53, 0.68) | Ref |
| TV 0-5 | Inter-day | 0.40 (0.34, 0.46) | <0.001 |
| TV 0-5 | Intra-day | 0.59 (0.51, 0.67) | Ref |
| TV 0-6 | Inter-day | 0.41 (0.35, 0.47) | <0.001 |
| TV 0-6 | Intra-day | 0.62 (0.53, 0.70) | Ref |
| TV 0-7 | Inter-day | 0.44 (0.37, 0.50) | <0.001 |
| TV 0-7 | Intra-day | 0.64 (0.56, 0.73) | Ref |
| Respiratory |  |  |  |
| TV 0-1 | Inter-day | 0.05 (-0.04, 0.13) | 0.004 |
| TV 0-1 | Intra-day | 0.25 (0.14, 0.36) | Ref |
| TV 0-2 | Inter-day | 0.02 (-0.06, 0.10) | <0.001 |
| TV 0-2 | Intra-day | 0.35 (0.23, 0.46) | Ref |
| TV 0-3 | Inter-day | 0.18 (0.09, 0.27) | 0.025 |
| TV 0-3 | Intra-day | 0.35 (0.23, 0.47) | Ref |
| TV 0-4 | Inter-day | 0.30 (0.20, 0.39) | 0.276 |
| TV 0-4 | Intra-day | 0.39 (0.25, 0.53) | Ref |
| TV 0-5 | Inter-day | 0.33 (0.22, 0.43) | 0.139 |
| TV 0-5 | Intra-day | 0.46 (0.31, 0.61) | Ref |
| TV 0-6 | Inter-day | 0.31 (0.21, 0.41) | 0.017 |
| TV 0-6 | Intra-day | 0.54 (0.38, 0.69) | Ref |
| TV 0-7 | Inter-day | 0.31 (0.21, 0.41) | <0.001 |
| TV 0-7 | Intra-day | 0.65 (0.49, 0.80) | Ref |

## Table S5. Percentage change (%) of all-cause mortality risk per IQR increase in TV.

| Country/region | TV 0–1 | | TV 0–2 | | TV 0–3 | | TV 0–4 | | TV 0–5 | | TV 0–6 | | TV 0–7 | |
| --- | --- | --- | --- | --- | --- | --- | --- | --- | --- | --- | --- | --- | --- | --- |
|  | Inter-day | Intra-day | Inter-day | Intra-day | Inter-day | Intra-day | Inter-day | Intra-day | Inter-day | Intra-day | Inter-day | Intra-day | Inter-day | Intra-day |
| Argentina | -0.07 (-0.22, 0.07) | 1.08 (0.77, 1.39) | -0.12 (-0.26, 0.02) | 0.94 (0.69, 1.19) | -0.04 (-0.20, 0.11) | 0.91 (0.65, 1.17) | 0.02 (-0.16, 0.21) | 0.85 (0.57, 1.13) | 0.07 (-0.14, 0.27) | 0.82 (0.53, 1.11) | 0.10 (-0.11, 0.31) | 0.81 (0.51, 1.11) | 0.07 (-0.15, 0.29) | 0.83 (0.52, 1.15) |
| Australia | 0.02 (-0.12, 0.16) | 0.25 (-0.07, 0.58) | -0.06 (-0.19, 0.08) | 0.51 (0.26, 0.76) | 0.06 (-0.10, 0.21) | 0.53 (0.27, 0.79) | 0.18 (-0.00, 0.36) | 0.50 (0.22, 0.78) | 0.22 (0.01, 0.43) | 0.54 (0.24, 0.83) | 0.25 (0.04, 0.47) | 0.59 (0.25, 0.92) | 0.29 (0.07, 0.51) | 0.66 (0.22, 1.09) |
| Brazil | -0.04 (-0.10, 0.02) | 0.74 (0.59, 0.90) | -0.06 (-0.12, -0.00) | 0.66 (0.55, 0.77) | 0.06 (-0.00, 0.13) | 0.57 (0.46, 0.69) | 0.14 (0.06, 0.21) | 0.53 (0.41, 0.65) | 0.17 (0.08, 0.26) | 0.53 (0.41, 0.66) | 0.16 (0.07, 0.25) | 0.57 (0.44, 0.70) | 0.17 (0.07, 0.26) | 0.64 (0.49, 0.79) |
| Canada | -0.03 (-0.08, 0.02) | 0.57 (0.45, 0.70) | -0.04 (-0.09, 0.01) | 0.59 (0.50, 0.69) | 0.07 (0.02, 0.13) | 0.55 (0.45, 0.65) | 0.16 (0.10, 0.23) | 0.50 (0.40, 0.61) | 0.21 (0.13, 0.28) | 0.51 (0.40, 0.62) | 0.20 (0.13, 0.28) | 0.54 (0.42, 0.65) | 0.20 (0.12, 0.28) | 0.58 (0.46, 0.70) |
| Chile | -0.03 (-0.17, 0.10) | 0.37 (-0.08, 0.82) | -0.04 (-0.16, 0.08) | 0.46 (0.21, 0.70) | 0.05 (-0.09, 0.19) | 0.39 (0.11, 0.67) | 0.14 (-0.03, 0.31) | 0.31 (-0.03, 0.64) | 0.17 (-0.02, 0.36) | 0.27 (-0.07, 0.62) | 0.18 (-0.02, 0.38) | 0.28 (-0.08, 0.64) | 0.18 (-0.03, 0.38) | 0.28 (-0.10, 0.66) |
| China | -0.02 (-0.09, 0.05) | 0.61 (0.42, 0.79) | -0.04 (-0.10, 0.03) | 0.63 (0.50, 0.76) | 0.07 (-0.00, 0.15) | 0.61 (0.47, 0.74) | 0.17 (0.08, 0.26) | 0.60 (0.45, 0.75) | 0.23 (0.12, 0.33) | 0.61 (0.45, 0.76) | 0.24 (0.13, 0.35) | 0.63 (0.46, 0.79) | 0.25 (0.14, 0.37) | 0.65 (0.48, 0.83) |
| Colombia | 0.00 (-0.11, 0.11) | 0.51 (0.24, 0.77) | -0.02 (-0.13, 0.09) | 0.54 (0.34, 0.74) | 0.10 (-0.02, 0.22) | 0.40 (0.19, 0.62) | 0.15 (0.01, 0.30) | 0.37 (0.14, 0.60) | 0.18 (0.02, 0.34) | 0.36 (0.12, 0.60) | 0.19 (0.02, 0.36) | 0.33 (0.08, 0.58) | 0.21 (0.04, 0.38) | 0.33 (0.07, 0.60) |
| Costa Rica | -0.04 (-0.31, 0.23) | 0.71 (-0.04, 1.47) | -0.03 (-0.29, 0.23) | 0.60 (0.09, 1.10) | 0.10 (-0.19, 0.38) | 0.50 (-0.04, 1.03) | 0.19 (-0.16, 0.55) | 0.42 (-0.17, 1.00) | 0.26 (-0.15, 0.67) | 0.42 (-0.19, 1.03) | 0.27 (-0.15, 0.69) | 0.42 (-0.22, 1.04) | 0.30 (-0.14, 0.74) | 0.38 (-0.30, 1.06) |
| Czech Republic | -0.02 (-0.14, 0.11) | 0.28 (-0.05, 0.61) | -0.00 (-0.13, 0.12) | 0.47 (0.23, 0.71) | 0.10 (-0.03, 0.24) | 0.42 (0.17, 0.68) | 0.21 (0.05, 0.37) | 0.40 (0.12, 0.67) | 0.27 (0.09, 0.45) | 0.40 (0.12, 0.69) | 0.25 (0.07, 0.44) | 0.45 (0.16, 0.75) | 0.27 (0.08, 0.46) | 0.50 (0.18, 0.81) |
| Ecuador | 0.01 (-0.18, 0.19) | 0.80 (0.27, 1.33) | -0.04 (-0.22, 0.14) | 0.62 (0.26, 0.98) | 0.05 (-0.15, 0.25) | 0.60 (0.22, 0.98) | 0.12 (-0.12, 0.37) | 0.59 (0.17, 1.01) | 0.13 (-0.15, 0.42) | 0.60 (0.16, 1.03) | 0.12 (-0.18, 0.41) | 0.59 (0.14, 1.04) | 0.13 (-0.18, 0.43) | 0.63 (0.14, 1.11) |
| Estonia | -0.01 (-0.13, 0.11) | 0.57 (0.21, 0.92) | -0.03 (-0.15, 0.08) | 0.60 (0.36, 0.83) | 0.09 (-0.04, 0.21) | 0.54 (0.29, 0.78) | 0.19 (0.03, 0.35) | 0.52 (0.24, 0.79) | 0.22 (0.03, 0.40) | 0.53 (0.25, 0.81) | 0.21 (0.03, 0.40) | 0.57 (0.28, 0.86) | 0.22 (0.03, 0.42) | 0.62 (0.30, 0.93) |
| Finland | -0.11 (-0.36, 0.15) | 0.59 (-0.08, 1.25) | -0.08 (-0.33, 0.16) | 0.59 (0.11, 1.08) | 0.06 (-0.21, 0.33) | 0.53 (0.01, 1.04) | 0.13 (-0.20, 0.46) | 0.55 (-0.01, 1.11) | 0.19 (-0.18, 0.56) | 0.54 (-0.04, 1.11) | 0.18 (-0.20, 0.56) | 0.55 (-0.05, 1.15) | 0.13 (-0.26, 0.52) | 0.58 (-0.06, 1.22) |
| France | 0.00 (-0.06, 0.07) | 0.52 (0.35, 0.69) | -0.03 (-0.09, 0.03) | 0.56 (0.45, 0.68) | 0.07 (0.00, 0.13) | 0.50 (0.37, 0.62) | 0.15 (0.07, 0.23) | 0.47 (0.33, 0.60) | 0.17 (0.07, 0.27) | 0.47 (0.33, 0.62) | 0.17 (0.06, 0.28) | 0.51 (0.36, 0.65) | 0.16 (0.05, 0.27) | 0.55 (0.39, 0.71) |
| French Caribbean | -0.02 (-0.22, 0.17) | 0.86 (0.30, 1.42) | -0.04 (-0.22, 0.15) | 0.70 (0.33, 1.07) | 0.06 (-0.14, 0.27) | 0.66 (0.27, 1.05) | 0.16 (-0.10, 0.41) | 0.63 (0.20, 1.06) | 0.19 (-0.10, 0.48) | 0.60 (0.16, 1.04) | 0.18 (-0.12, 0.48) | 0.62 (0.16, 1.07) | 0.19 (-0.13, 0.50) | 0.61 (0.11, 1.10) |
| French Guiana | -0.02 (-0.29, 0.26) | 0.63 (-0.21, 1.46) | -0.04 (-0.29, 0.22) | 0.60 (0.07, 1.13) | 0.09 (-0.20, 0.38) | 0.55 (-0.02, 1.11) | 0.20 (-0.16, 0.57) | 0.52 (-0.11, 1.14) | 0.25 (-0.17, 0.67) | 0.53 (-0.11, 1.17) | 0.25 (-0.18, 0.68) | 0.56 (-0.11, 1.22) | 0.25 (-0.20, 0.70) | 0.61 (-0.11, 1.33) |
| Réunion | 0.02 (-0.26, 0.29) | 0.49 (-0.31, 1.30) | -0.02 (-0.28, 0.23) | 0.57 (0.05, 1.09) | 0.06 (-0.23, 0.35) | 0.51 (-0.05, 1.06) | 0.14 (-0.22, 0.50) | 0.48 (-0.13, 1.08) | 0.18 (-0.24, 0.60) | 0.41 (-0.22, 1.04) | 0.19 (-0.24, 0.62) | 0.43 (-0.23, 1.08) | 0.21 (-0.24, 0.65) | 0.45 (-0.26, 1.15) |
| Germany | 0.01 (-0.06, 0.08) | 0.34 (0.16, 0.51) | -0.02 (-0.09, 0.05) | 0.50 (0.36, 0.64) | 0.05 (-0.02, 0.13) | 0.45 (0.31, 0.60) | 0.13 (0.05, 0.22) | 0.42 (0.26, 0.57) | 0.15 (0.05, 0.24) | 0.43 (0.27, 0.60) | 0.13 (0.03, 0.23) | 0.48 (0.31, 0.65) | 0.13 (0.03, 0.24) | 0.55 (0.37, 0.73) |
| Greece | 0.05 (-0.19, 0.29) | 0.52 (-0.07, 1.10) | -0.02 (-0.25, 0.21) | 0.66 (0.21, 1.12) | 0.09 (-0.16, 0.35) | 0.70 (0.22, 1.19) | 0.20 (-0.10, 0.50) | 0.74 (0.21, 1.27) | 0.27 (-0.06, 0.61) | 0.74 (0.19, 1.29) | 0.30 (-0.05, 0.64) | 0.76 (0.19, 1.33) | 0.26 (-0.10, 0.62) | 0.82 (0.21, 1.42) |
| Guatemala | -0.04 (-0.31, 0.22) | 0.82 (0.11, 1.53) | -0.05 (-0.30, 0.20) | 0.63 (0.12, 1.13) | 0.08 (-0.21, 0.36) | 0.46 (-0.08, 0.99) | 0.13 (-0.21, 0.48) | 0.34 (-0.25, 0.93) | 0.14 (-0.26, 0.54) | 0.34 (-0.27, 0.96) | 0.10 (-0.31, 0.51) | 0.36 (-0.28, 0.99) | 0.06 (-0.36, 0.49) | 0.42 (-0.27, 1.11) |
| Iran | -0.05 (-0.21, 0.11) | 0.14 (-0.22, 0.49) | -0.07 (-0.23, 0.09) | 0.38 (0.09, 0.68) | 0.06 (-0.12, 0.24) | 0.39 (0.07, 0.70) | 0.14 (-0.07, 0.35) | 0.32 (-0.02, 0.67) | 0.27 (0.04, 0.51) | 0.22 (-0.14, 0.58) | 0.28 (0.03, 0.52) | 0.21 (-0.16, 0.59) | 0.29 (0.04, 0.54) | 0.29 (-0.11, 0.69) |
| Ireland | -0.03 (-0.14, 0.07) | 0.36 (0.13, 0.59) | -0.05 (-0.15, 0.05) | 0.45 (0.27, 0.63) | 0.07 (-0.04, 0.18) | 0.38 (0.19, 0.57) | 0.12 (-0.01, 0.25) | 0.33 (0.13, 0.54) | 0.17 (0.02, 0.32) | 0.33 (0.12, 0.55) | 0.20 (0.05, 0.36) | 0.32 (0.10, 0.54) | 0.24 (0.08, 0.40) | 0.32 (0.08, 0.55) |
| Israel | 0.05 (-0.18, 0.28) | 0.61 (0.17, 1.04) | 0.07 (-0.15, 0.29) | 0.71 (0.32, 1.10) | 0.15 (-0.10, 0.40) | 0.71 (0.29, 1.12) | 0.21 (-0.09, 0.51) | 0.72 (0.28, 1.17) | 0.23 (-0.11, 0.57) | 0.71 (0.25, 1.17) | 0.20 (-0.16, 0.56) | 0.70 (0.22, 1.18) | 0.23 (-0.14, 0.61) | 0.61 (0.10, 1.12) |
| Italy | -0.02 (-0.10, 0.06) | 0.45 (0.24, 0.67) | -0.04 (-0.12, 0.04) | 0.58 (0.43, 0.73) | 0.07 (-0.02, 0.15) | 0.56 (0.40, 0.72) | 0.17 (0.07, 0.28) | 0.54 (0.37, 0.71) | 0.22 (0.10, 0.34) | 0.55 (0.37, 0.73) | 0.23 (0.11, 0.35) | 0.58 (0.40, 0.77) | 0.25 (0.12, 0.37) | 0.63 (0.43, 0.83) |
| Japan | 0.03 (-0.00, 0.06) | 0.48 (0.42, 0.54) | 0.00 (-0.03, 0.03) | 0.61 (0.56, 0.65) | 0.12 (0.09, 0.15) | 0.61 (0.57, 0.66) | 0.25 (0.21, 0.29) | 0.64 (0.59, 0.69) | 0.32 (0.27, 0.36) | 0.68 (0.63, 0.74) | 0.31 (0.26, 0.35) | 0.73 (0.67, 0.79) | 0.30 (0.25, 0.34) | 0.80 (0.74, 0.87) |
| Kuwait | -0.04 (-0.30, 0.22) | 0.44 (-0.24, 1.12) | -0.06 (-0.31, 0.18) | 0.58 (0.09, 1.07) | 0.06 (-0.21, 0.34) | 0.60 (0.08, 1.11) | 0.14 (-0.20, 0.47) | 0.62 (0.05, 1.19) | 0.14 (-0.25, 0.53) | 0.64 (0.05, 1.22) | 0.15 (-0.25, 0.55) | 0.65 (0.04, 1.26) | 0.18 (-0.23, 0.59) | 0.68 (0.02, 1.33) |
| Mexico | -0.09 (-0.16, -0.01) | 0.78 (0.59, 0.98) | -0.08 (-0.15, -0.01) | 0.69 (0.54, 0.84) | 0.01 (-0.07, 0.09) | 0.65 (0.49, 0.80) | 0.07 (-0.03, 0.16) | 0.65 (0.48, 0.82) | 0.09 (-0.02, 0.20) | 0.64 (0.47, 0.82) | 0.09 (-0.03, 0.21) | 0.67 (0.49, 0.85) | 0.09 (-0.04, 0.22) | 0.74 (0.54, 0.93) |
| Moldova | -0.03 (-0.16, 0.11) | 0.57 (0.16, 0.98) | -0.03 (-0.16, 0.09) | 0.59 (0.32, 0.85) | 0.08 (-0.07, 0.22) | 0.53 (0.26, 0.81) | 0.18 (-0.00, 0.36) | 0.51 (0.20, 0.82) | 0.22 (0.01, 0.43) | 0.52 (0.20, 0.83) | 0.22 (0.01, 0.44) | 0.54 (0.21, 0.87) | 0.23 (0.01, 0.46) | 0.58 (0.23, 0.94) |
| Netherland | -0.01 (-0.13, 0.10) | 0.51 (0.20, 0.83) | -0.04 (-0.15, 0.07) | 0.57 (0.35, 0.79) | 0.05 (-0.07, 0.18) | 0.51 (0.27, 0.74) | 0.14 (-0.01, 0.29) | 0.45 (0.18, 0.70) | 0.15 (-0.02, 0.33) | 0.45 (0.18, 0.72) | 0.13 (-0.05, 0.30) | 0.47 (0.19, 0.75) | 0.13 (-0.05, 0.32) | 0.51 (0.21, 0.81) |
| Norway | 0.10 (-0.15, 0.34) | -0.00 (-0.62, 0.61) | 0.07 (-0.17, 0.31) | 0.34 (-0.13, 0.80) | 0.21 (-0.06, 0.47) | 0.30 (-0.19, 0.78) | 0.31 (-0.01, 0.63) | 0.37 (-0.17, 0.89) | 0.38 (0.02, 0.75) | 0.39 (-0.16, 0.93) | 0.28 (-0.09, 0.66) | 0.45 (-0.12, 1.02) | 0.28 (-0.11, 0.67) | 0.50 (-0.11, 1.11) |
| Panama | -0.00 (-0.28, 0.27) | 0.57 (-0.25, 1.39) | -0.03 (-0.29, 0.22) | 0.58 (0.05, 1.11) | 0.08 (-0.21, 0.37) | 0.54 (-0.01, 1.10) | 0.19 (-0.17, 0.55) | 0.53 (-0.09, 1.14) | 0.23 (-0.19, 0.65) | 0.54 (-0.09, 1.18) | 0.22 (-0.21, 0.65) | 0.58 (-0.08, 1.23) | 0.23 (-0.22, 0.68) | 0.61 (-0.11, 1.32) |
| Paraguay | -0.05 (-0.32, 0.22) | 1.12 (0.40, 1.83) | -0.09 (-0.34, 0.17) | 0.74 (0.24, 1.24) | -0.04 (-0.32, 0.25) | 0.63 (0.10, 1.15) | -0.01 (-0.36, 0.34) | 0.53 (-0.06, 1.10) | -0.01 (-0.42, 0.40) | 0.52 (-0.07, 1.12) | 0.02 (-0.40, 0.44) | 0.51 (-0.11, 1.13) | 0.04 (-0.40, 0.48) | 0.53 (-0.14, 1.19) |
| Peru | -0.02 (-0.09, 0.04) | 0.63 (0.44, 0.82) | -0.03 (-0.09, 0.03) | 0.60 (0.48, 0.72) | 0.07 (0.01, 0.14) | 0.55 (0.42, 0.68) | 0.17 (0.08, 0.25) | 0.52 (0.38, 0.66) | 0.20 (0.10, 0.30) | 0.52 (0.38, 0.67) | 0.19 (0.09, 0.29) | 0.56 (0.40, 0.71) | 0.20 (0.09, 0.30) | 0.60 (0.43, 0.76) |
| Philippines | -0.05 (-0.12, 0.03) | 0.50 (0.29, 0.71) | -0.05 (-0.11, 0.02) | 0.53 (0.39, 0.67) | 0.07 (-0.01, 0.14) | 0.44 (0.29, 0.58) | 0.14 (0.05, 0.24) | 0.41 (0.24, 0.57) | 0.19 (0.08, 0.29) | 0.43 (0.26, 0.60) | 0.21 (0.10, 0.32) | 0.46 (0.28, 0.63) | 0.20 (0.09, 0.32) | 0.51 (0.32, 0.70) |
| Portugal | -0.02 (-0.11, 0.08) | 0.26 (0.03, 0.49) | -0.05 (-0.14, 0.05) | 0.56 (0.38, 0.74) | 0.01 (-0.10, 0.11) | 0.60 (0.41, 0.79) | 0.07 (-0.06, 0.19) | 0.63 (0.43, 0.84) | 0.08 (-0.06, 0.22) | 0.62 (0.41, 0.83) | 0.09 (-0.05, 0.23) | 0.64 (0.42, 0.86) | 0.10 (-0.05, 0.25) | 0.68 (0.45, 0.91) |
| Puerto Rico | -0.00 (-0.27, 0.26) | 0.55 (-0.20, 1.29) | -0.01 (-0.27, 0.24) | 0.56 (0.05, 1.07) | 0.09 (-0.20, 0.38) | 0.47 (-0.07, 1.01) | 0.16 (-0.20, 0.51) | 0.46 (-0.13, 1.05) | 0.15 (-0.26, 0.56) | 0.50 (-0.11, 1.11) | 0.15 (-0.27, 0.57) | 0.54 (-0.09, 1.17) | 0.15 (-0.29, 0.58) | 0.55 (-0.13, 1.23) |
| Romania | -0.00 (-0.09, 0.09) | 0.63 (0.38, 0.87) | -0.01 (-0.10, 0.08) | 0.60 (0.43, 0.78) | 0.11 (0.01, 0.21) | 0.56 (0.38, 0.75) | 0.20 (0.08, 0.31) | 0.56 (0.36, 0.76) | 0.23 (0.10, 0.37) | 0.56 (0.35, 0.77) | 0.23 (0.09, 0.37) | 0.60 (0.38, 0.82) | 0.23 (0.09, 0.38) | 0.67 (0.43, 0.90) |
| South Africa | -0.06 (-0.09, -0.02) | 0.82 (0.71, 0.93) | -0.05 (-0.08, -0.02) | 0.58 (0.51, 0.64) | 0.08 (0.04, 0.12) | 0.48 (0.41, 0.55) | 0.20 (0.15, 0.24) | 0.44 (0.36, 0.52) | 0.25 (0.20, 0.30) | 0.46 (0.38, 0.54) | 0.24 (0.19, 0.30) | 0.51 (0.43, 0.60) | 0.23 (0.18, 0.29) | 0.58 (0.49, 0.67) |
| South Korea | -0.04 (-0.08, 0.01) | 0.51 (0.39, 0.62) | -0.04 (-0.09, -0.00) | 0.57 (0.48, 0.65) | 0.07 (0.02, 0.12) | 0.53 (0.44, 0.61) | 0.16 (0.11, 0.22) | 0.51 (0.41, 0.60) | 0.21 (0.15, 0.28) | 0.52 (0.42, 0.62) | 0.21 (0.14, 0.28) | 0.57 (0.46, 0.67) | 0.22 (0.15, 0.29) | 0.63 (0.52, 0.74) |
| Spain | -0.02 (-0.06, 0.01) | 0.44 (0.34, 0.54) | -0.04 (-0.07, -0.00) | 0.55 (0.48, 0.62) | 0.07 (0.03, 0.11) | 0.51 (0.44, 0.59) | 0.15 (0.11, 0.20) | 0.50 (0.42, 0.58) | 0.19 (0.13, 0.24) | 0.52 (0.43, 0.60) | 0.19 (0.13, 0.24) | 0.55 (0.46, 0.63) | 0.19 (0.14, 0.25) | 0.59 (0.50, 0.68) |
| Sweden | 0.05 (-0.10, 0.19) | 0.05 (-0.43, 0.53) | 0.03 (-0.11, 0.17) | 0.35 (0.07, 0.62) | 0.11 (-0.04, 0.26) | 0.32 (0.03, 0.61) | 0.20 (0.02, 0.38) | 0.25 (-0.07, 0.56) | 0.22 (0.02, 0.42) | 0.26 (-0.07, 0.59) | 0.24 (0.03, 0.45) | 0.32 (-0.02, 0.66) | 0.24 (0.02, 0.45) | 0.36 (-0.01, 0.73) |
| Switzerland | -0.01 (-0.11, 0.09) | 0.49 (0.20, 0.77) | -0.03 (-0.12, 0.06) | 0.57 (0.38, 0.75) | 0.07 (-0.03, 0.17) | 0.50 (0.31, 0.70) | 0.15 (0.02, 0.28) | 0.48 (0.26, 0.69) | 0.17 (0.03, 0.32) | 0.49 (0.27, 0.72) | 0.18 (0.03, 0.33) | 0.54 (0.31, 0.77) | 0.19 (0.04, 0.35) | 0.59 (0.34, 0.84) |
| Taiwan | -0.02 (-0.15, 0.12) | 0.43 (-0.06, 0.92) | -0.06 (-0.19, 0.08) | 0.52 (0.28, 0.75) | 0.03 (-0.11, 0.18) | 0.60 (0.35, 0.84) | 0.07 (-0.11, 0.25) | 0.64 (0.38, 0.91) | 0.05 (-0.15, 0.25) | 0.63 (0.36, 0.91) | 0.08 (-0.13, 0.28) | 0.63 (0.34, 0.92) | 0.09 (-0.13, 0.30) | 0.67 (0.37, 0.98) |
| Thailand | -0.03 (-0.06, 0.01) | 0.68 (0.58, 0.78) | -0.04 (-0.07, -0.01) | 0.61 (0.54, 0.68) | 0.07 (0.04, 0.11) | 0.55 (0.48, 0.62) | 0.17 (0.12, 0.21) | 0.51 (0.44, 0.59) | 0.20 (0.15, 0.25) | 0.52 (0.44, 0.60) | 0.20 (0.14, 0.25) | 0.55 (0.46, 0.63) | 0.19 (0.14, 0.25) | 0.60 (0.51, 0.69) |
| UK | -0.02 (-0.05, 0.01) | 0.51 (0.43, 0.59) | -0.03 (-0.06, -0.00) | 0.56 (0.50, 0.61) | 0.08 (0.05, 0.11) | 0.48 (0.42, 0.55) | 0.18 (0.14, 0.22) | 0.44 (0.37, 0.51) | 0.22 (0.17, 0.26) | 0.44 (0.37, 0.51) | 0.22 (0.17, 0.27) | 0.47 (0.40, 0.54) | 0.23 (0.18, 0.28) | 0.51 (0.44, 0.59) |
| Uruguay | 0.03 (-0.22, 0.28) | 0.72 (0.06, 1.36) | -0.08 (-0.33, 0.16) | 0.77 (0.31, 1.23) | -0.02 (-0.29, 0.25) | 0.72 (0.23, 1.20) | 0.08 (-0.25, 0.40) | 0.69 (0.18, 1.20) | 0.11 (-0.26, 0.48) | 0.67 (0.15, 1.18) | 0.12 (-0.26, 0.49) | 0.80 (0.27, 1.33) | 0.20 (-0.19, 0.59) | 0.79 (0.23, 1.34) |
| USA | -0.02 (-0.04, -0.01) | 0.59 (0.55, 0.63) | -0.04 (-0.06, -0.02) | 0.61 (0.57, 0.64) | 0.08 (0.06, 0.10) | 0.55 (0.51, 0.58) | 0.18 (0.16, 0.21) | 0.51 (0.47, 0.54) | 0.24 (0.21, 0.26) | 0.51 (0.47, 0.55) | 0.24 (0.21, 0.27) | 0.54 (0.50, 0.58) | 0.25 (0.22, 0.27) | 0.59 (0.55, 0.63) |
| Vietnam | -0.04 (-0.23, 0.15) | 0.39 (-0.14, 0.91) | -0.06 (-0.24, 0.12) | 0.51 (0.15, 0.87) | 0.04 (-0.16, 0.24) | 0.45 (0.07, 0.82) | 0.12 (-0.13, 0.37) | 0.39 (-0.03, 0.80) | 0.16 (-0.13, 0.45) | 0.43 (0.00, 0.86) | 0.19 (-0.11, 0.49) | 0.46 (0.02, 0.90) | 0.23 (-0.08, 0.54) | 0.51 (0.04, 0.98) |

## Table S6. Percentage change (%) of cardiovascular mortality risk per IQR increase in TV.

| Country/region | TV 0–1 | | TV 0–2 | | TV 0–3 | | TV 0–4 | | TV 0–5 | | TV 0–6 | | TV 0–7 | |
| --- | --- | --- | --- | --- | --- | --- | --- | --- | --- | --- | --- | --- | --- | --- |
|  | Inter-day | Intra-day | Inter-day | Intra-day | Inter-day | Intra-day | Inter-day | Intra-day | Inter-day | Intra-day | Inter-day | Intra-day | Inter-day | Intra-day |
| Brazil | 0.09 (0.08, 0.10) | 0.57 (0.44, 0.70) | 0.10 (0.03, 0.18) | 0.63 (0.51, 0.74) | 0.23 (0.14, 0.32) | 0.63 (0.50, 0.76) | 0.32 (0.21, 0.44) | 0.62 (0.48, 0.76) | 0.39 (0.27, 0.51) | 0.60 (0.46, 0.74) | 0.41 (0.29, 0.53) | 0.62 (0.47, 0.77) | 0.45 (0.32, 0.57) | 0.65 (0.49, 0.81) |
| Canada | 0.09 (0.08, 0.09) | 0.54 (0.43, 0.64) | 0.12 (0.06, 0.18) | 0.61 (0.52, 0.70) | 0.23 (0.16, 0.30) | 0.63 (0.53, 0.73) | 0.34 (0.26, 0.43) | 0.63 (0.52, 0.73) | 0.41 (0.33, 0.50) | 0.61 (0.51, 0.72) | 0.42 (0.34, 0.51) | 0.66 (0.54, 0.77) | 0.45 (0.36, 0.54) | 0.68 (0.56, 0.79) |
| China | 0.09 (0.07, 0.10) | 0.54 (0.40, 0.69) | 0.11 (0.03, 0.19) | 0.65 (0.53, 0.78) | 0.27 (0.17, 0.36) | 0.65 (0.51, 0.80) | 0.40 (0.27, 0.52) | 0.64 (0.49, 0.79) | 0.47 (0.34, 0.59) | 0.62 (0.47, 0.78) | 0.47 (0.35, 0.60) | 0.63 (0.47, 0.80) | 0.50 (0.37, 0.63) | 0.65 (0.47, 0.82) |
| Colombia | 0.09 (0.07, 0.10) | 0.47 (0.23, 0.70) | 0.11 (-0.03, 0.25) | 0.55 (0.34, 0.76) | 0.22 (0.05, 0.38) | 0.51 (0.27, 0.75) | 0.27 (0.07, 0.48) | 0.49 (0.25, 0.74) | 0.34 (0.13, 0.55) | 0.47 (0.21, 0.72) | 0.36 (0.14, 0.57) | 0.46 (0.19, 0.73) | 0.38 (0.15, 0.60) | 0.44 (0.16, 0.73) |
| Costa Rica | 0.09 (0.04, 0.13) | 0.60 (0.04, 1.15) | 0.11 (-0.20, 0.42) | 0.59 (0.11, 1.07) | 0.23 (-0.14, 0.61) | 0.62 (0.07, 1.17) | 0.31 (-0.18, 0.80) | 0.63 (0.04, 1.20) | 0.44 (-0.05, 0.93) | 0.58 (-0.02, 1.18) | 0.44 (-0.07, 0.94) | 0.65 (0.01, 1.28) | 0.47 (-0.05, 0.99) | 0.67 (-0.01, 1.35) |
| Czech Republic | 0.09 (0.06, 0.11) | 0.41 (0.15, 0.67) | 0.13 (-0.02, 0.28) | 0.60 (0.36, 0.84) | 0.24 (0.05, 0.42) | 0.60 (0.32, 0.87) | 0.30 (0.06, 0.54) | 0.60 (0.31, 0.88) | 0.38 (0.14, 0.62) | 0.57 (0.27, 0.87) | 0.37 (0.12, 0.62) | 0.60 (0.28, 0.91) | 0.41 (0.15, 0.66) | 0.61 (0.28, 0.95) |
| Ecuador | 0.09 (0.06, 0.12) | 0.58 (0.18, 0.97) | 0.03 (-0.18, 0.24) | 0.81 (0.49, 1.13) | 0.16 (-0.09, 0.42) | 0.83 (0.46, 1.19) | 0.28 (-0.05, 0.60) | 0.83 (0.44, 1.21) | 0.42 (0.09, 0.74) | 0.77 (0.38, 1.17) | 0.42 (0.09, 0.75) | 0.83 (0.41, 1.24) | 0.45 (0.11, 0.80) | 0.81 (0.37, 1.25) |
| Estonia | 0.09 (0.07, 0.10) | 0.53 (0.28, 0.78) | 0.13 (-0.01, 0.27) | 0.60 (0.39, 0.81) | 0.26 (0.10, 0.43) | 0.59 (0.36, 0.83) | 0.42 (0.21, 0.63) | 0.60 (0.35, 0.85) | 0.49 (0.28, 0.70) | 0.58 (0.32, 0.84) | 0.49 (0.27, 0.70) | 0.61 (0.33, 0.88) | 0.52 (0.29, 0.74) | 0.63 (0.33, 0.92) |
| Finland | 0.09 (0.04, 0.13) | 0.48 (-0.06, 1.02) | 0.10 (-0.21, 0.41) | 0.60 (0.12, 1.09) | 0.22 (-0.16, 0.60) | 0.59 (0.02, 1.15) | 0.33 (-0.17, 0.83) | 0.55 (-0.05, 1.14) | 0.42 (-0.08, 0.92) | 0.53 (-0.09, 1.14) | 0.42 (-0.10, 0.94) | 0.55 (-0.11, 1.20) | 0.46 (-0.08, 0.99) | 0.59 (-0.11, 1.28) |
| Greece | 0.09 (0.04, 0.13) | 0.40 (-0.11, 0.90) | 0.09 (-0.20, 0.37) | 0.59 (0.14, 1.05) | 0.23 (-0.11, 0.56) | 0.68 (0.16, 1.20) | 0.32 (-0.10, 0.73) | 0.68 (0.13, 1.23) | 0.42 (-0.01, 0.84) | 0.65 (0.07, 1.21) | 0.44 (-0.00, 0.87) | 0.65 (0.04, 1.25) | 0.45 (-0.01, 0.90) | 0.66 (0.01, 1.29) |
| Iran | 0.09 (0.06, 0.12) | 0.47 (0.13, 0.80) | 0.09 (-0.13, 0.31) | 0.64 (0.30, 0.98) | 0.20 (-0.06, 0.47) | 0.64 (0.25, 1.03) | 0.31 (-0.04, 0.65) | 0.63 (0.23, 1.04) | 0.40 (0.05, 0.75) | 0.61 (0.18, 1.03) | 0.43 (0.07, 0.79) | 0.59 (0.14, 1.04) | 0.47 (0.09, 0.84) | 0.62 (0.14, 1.09) |
| Ireland | 0.09 (0.07, 0.10) | 0.49 (0.28, 0.70) | 0.12 (-0.01, 0.24) | 0.61 (0.42, 0.81) | 0.24 (0.08, 0.39) | 0.62 (0.39, 0.84) | 0.36 (0.16, 0.55) | 0.58 (0.35, 0.82) | 0.41 (0.21, 0.61) | 0.58 (0.33, 0.82) | 0.43 (0.22, 0.64) | 0.61 (0.35, 0.87) | 0.48 (0.26, 0.69) | 0.63 (0.36, 0.91) |
| Japan | 0.09 (0.08, 0.09) | 0.46 (0.40, 0.52) | 0.12 (0.07, 0.16) | 0.61 (0.54, 0.67) | 0.24 (0.19, 0.29) | 0.63 (0.56, 0.71) | 0.35 (0.29, 0.42) | 0.62 (0.54, 0.70) | 0.44 (0.37, 0.50) | 0.61 (0.53, 0.69) | 0.45 (0.38, 0.52) | 0.66 (0.57, 0.74) | 0.48 (0.41, 0.54) | 0.69 (0.60, 0.78) |
| Kuwait | 0.09 (0.04, 0.13) | 0.52 (-0.01, 1.05) | 0.06 (-0.25, 0.37) | 0.65 (0.17, 1.13) | 0.20 (-0.18, 0.57) | 0.62 (0.06, 1.17) | 0.27 (-0.22, 0.75) | 0.59 (0.01, 1.17) | 0.33 (-0.16, 0.82) | 0.55 (-0.05, 1.16) | 0.37 (-0.13, 0.87) | 0.55 (-0.09, 1.19) | 0.39 (-0.13, 0.91) | 0.56 (-0.12, 1.24) |
| Mexico | 0.09 (0.07, 0.10) | 0.51 (0.34, 0.68) | 0.09 (-0.01, 0.19) | 0.59 (0.44, 0.74) | 0.20 (0.08, 0.32) | 0.58 (0.41, 0.75) | 0.29 (0.14, 0.44) | 0.57 (0.39, 0.75) | 0.39 (0.23, 0.54) | 0.54 (0.36, 0.73) | 0.40 (0.24, 0.55) | 0.58 (0.38, 0.77) | 0.44 (0.28, 0.60) | 0.58 (0.37, 0.79) |
| Norway | 0.09 (0.04, 0.13) | 0.41 (-0.12, 0.93) | 0.12 (-0.19, 0.43) | 0.51 (0.03, 0.98) | 0.23 (-0.15, 0.60) | 0.46 (-0.09, 1.01) | 0.36 (-0.13, 0.84) | 0.36 (-0.22, 0.94) | 0.44 (-0.05, 0.93) | 0.37 (-0.23, 0.96) | 0.43 (-0.07, 0.94) | 0.40 (-0.24, 1.03) | 0.47 (-0.06, 0.99) | 0.42 (-0.26, 1.08) |
| Panama | 0.09 (0.04, 0.13) | 0.55 (-0.02, 1.11) | 0.12 (-0.19, 0.43) | 0.62 (0.13, 1.10) | 0.23 (-0.15, 0.61) | 0.63 (0.07, 1.19) | 0.35 (-0.15, 0.85) | 0.62 (0.03, 1.21) | 0.46 (-0.04, 0.95) | 0.60 (-0.02, 1.21) | 0.45 (-0.07, 0.96) | 0.65 (-0.01, 1.30) | 0.47 (-0.07, 1.00) | 0.69 (-0.01, 1.38) |
| Paraguay | 0.09 (0.04, 0.13) | 0.68 (0.12, 1.22) | 0.09 (-0.23, 0.40) | 0.71 (0.23, 1.19) | 0.16 (-0.22, 0.54) | 0.67 (0.12, 1.23) | 0.22 (-0.28, 0.71) | 0.63 (0.05, 1.22) | 0.32 (-0.18, 0.82) | 0.61 (0.01, 1.22) | 0.34 (-0.17, 0.85) | 0.63 (-0.02, 1.26) | 0.37 (-0.17, 0.90) | 0.64 (-0.04, 1.32) |
| Philippines | 0.09 (0.07, 0.10) | 0.51 (0.36, 0.66) | 0.10 (0.01, 0.18) | 0.62 (0.48, 0.75) | 0.21 (0.11, 0.32) | 0.62 (0.47, 0.78) | 0.30 (0.16, 0.44) | 0.61 (0.44, 0.77) | 0.39 (0.25, 0.52) | 0.60 (0.43, 0.77) | 0.41 (0.27, 0.55) | 0.62 (0.44, 0.80) | 0.43 (0.28, 0.58) | 0.65 (0.45, 0.84) |
| Portugal | 0.09 (0.06, 0.11) | 0.45 (0.15, 0.75) | 0.09 (-0.13, 0.31) | 0.59 (0.25, 0.93) | 0.17 (-0.10, 0.43) | 0.61 (0.21, 1.00) | 0.22 (-0.12, 0.57) | 0.58 (0.16, 1.00) | 0.30 (-0.05, 0.64) | 0.56 (0.13, 1.00) | 0.30 (-0.06, 0.66) | 0.59 (0.13, 1.05) | 0.33 (-0.04, 0.70) | 0.62 (0.13, 1.11) |
| South Africa | 0.09 (0.08, 0.09) | 0.54 (0.47, 0.62) | 0.10 (0.05, 0.14) | 0.61 (0.54, 0.68) | 0.20 (0.15, 0.25) | 0.61 (0.53, 0.68) | 0.30 (0.23, 0.37) | 0.59 (0.52, 0.67) | 0.38 (0.32, 0.45) | 0.58 (0.50, 0.66) | 0.40 (0.33, 0.46) | 0.60 (0.52, 0.69) | 0.42 (0.35, 0.49) | 0.64 (0.55, 0.73) |
| South Korea | 0.09 (0.08, 0.09) | 0.52 (0.43, 0.62) | 0.10 (0.05, 0.15) | 0.60 (0.52, 0.67) | 0.22 (0.16, 0.28) | 0.60 (0.52, 0.69) | 0.34 (0.26, 0.41) | 0.61 (0.52, 0.70) | 0.41 (0.33, 0.49) | 0.61 (0.51, 0.71) | 0.42 (0.34, 0.50) | 0.63 (0.53, 0.73) | 0.45 (0.36, 0.53) | 0.66 (0.55, 0.77) |
| Spain | 0.09 (0.08, 0.09) | 0.49 (0.42, 0.57) | 0.10 (0.06, 0.14) | 0.62 (0.55, 0.68) | 0.21 (0.16, 0.26) | 0.62 (0.55, 0.70) | 0.30 (0.24, 0.37) | 0.61 (0.53, 0.69) | 0.38 (0.31, 0.45) | 0.60 (0.52, 0.68) | 0.39 (0.32, 0.46) | 0.62 (0.53, 0.70) | 0.42 (0.35, 0.49) | 0.65 (0.56, 0.74) |
| Sweden | 0.09 (0.06, 0.11) | 0.36 (0.05, 0.66) | 0.09 (-0.09, 0.27) | 0.59 (0.31, 0.86) | 0.20 (-0.02, 0.42) | 0.60 (0.28, 0.92) | 0.32 (0.04, 0.61) | 0.61 (0.27, 0.94) | 0.42 (0.13, 0.70) | 0.59 (0.25, 0.94) | 0.44 (0.15, 0.73) | 0.63 (0.26, 1.00) | 0.48 (0.18, 0.78) | 0.64 (0.25, 1.03) |
| Switzerland | 0.09 (0.07, 0.10) | 0.49 (0.29, 0.68) | 0.09 (-0.02, 0.19) | 0.66 (0.49, 0.82) | 0.18 (0.06, 0.31) | 0.68 (0.46, 0.89) | 0.30 (0.14, 0.46) | 0.66 (0.42, 0.90) | 0.41 (0.25, 0.57) | 0.66 (0.40, 0.91) | 0.44 (0.28, 0.61) | 0.72 (0.45, 0.99) | 0.46 (0.29, 0.63) | 0.77 (0.49, 1.05) |
| Taiwan | 0.09 (0.06, 0.11) | 0.54 (0.25, 0.84) | 0.12 (-0.06, 0.30) | 0.62 (0.34, 0.90) | 0.24 (0.02, 0.46) | 0.61 (0.29, 0.94) | 0.36 (0.08, 0.65) | 0.63 (0.29, 0.97) | 0.41 (0.12, 0.69) | 0.61 (0.26, 0.97) | 0.39 (0.10, 0.68) | 0.62 (0.24, 0.99) | 0.42 (0.11, 0.72) | 0.62 (0.23, 1.02) |
| Thailand | 0.09 (0.08, 0.09) | 0.53 (0.46, 0.60) | 0.10 (0.06, 0.14) | 0.61 (0.55, 0.67) | 0.22 (0.17, 0.27) | 0.62 (0.55, 0.69) | 0.32 (0.25, 0.38) | 0.61 (0.54, 0.68) | 0.39 (0.33, 0.45) | 0.59 (0.51, 0.66) | 0.40 (0.33, 0.46) | 0.61 (0.53, 0.69) | 0.42 (0.35, 0.48) | 0.63 (0.54, 0.71) |
| UK | 0.09 (0.08, 0.09) | 0.51 (0.45, 0.58) | 0.10 (0.07, 0.14) | 0.61 (0.55, 0.67) | 0.22 (0.18, 0.27) | 0.62 (0.56, 0.68) | 0.32 (0.27, 0.38) | 0.60 (0.53, 0.67) | 0.40 (0.34, 0.46) | 0.59 (0.52, 0.66) | 0.41 (0.35, 0.47) | 0.62 (0.54, 0.69) | 0.44 (0.38, 0.50) | 0.65 (0.58, 0.73) |
| USA | 0.09 (0.08, 0.09) | 0.54 (0.51, 0.58) | 0.10 (0.08, 0.12) | 0.62 (0.59, 0.66) | 0.21 (0.19, 0.24) | 0.63 (0.59, 0.66) | 0.32 (0.29, 0.35) | 0.62 (0.58, 0.65) | 0.40 (0.37, 0.43) | 0.60 (0.56, 0.64) | 0.41 (0.38, 0.45) | 0.63 (0.59, 0.67) | 0.44 (0.41, 0.48) | 0.66 (0.61, 0.70) |
| Vietnam | 0.09 (0.06, 0.12) | 0.49 (0.10, 0.88) | 0.08 (-0.13, 0.30) | 0.65 (0.33, 0.97) | 0.19 (-0.07, 0.45) | 0.70 (0.33, 1.06) | 0.31 (-0.03, 0.64) | 0.70 (0.31, 1.08) | 0.37 (0.03, 0.71) | 0.69 (0.29, 1.09) | 0.35 (-0.00, 0.70) | 0.73 (0.31, 1.15) | 0.36 (-0.00, 0.73) | 0.72 (0.28, 1.17) |

## Table S7. Percentage change (%) of respiratory mortality risk per IQR increase in TV.

| Country/region | TV 0–1 | | TV 0–2 | | TV 0–3 | | TV 0–4 | | TV 0–5 | | TV 0–6 | | TV 0–7 | |
| --- | --- | --- | --- | --- | --- | --- | --- | --- | --- | --- | --- | --- | --- | --- |
|  | Inter-day | Intra-day | Inter-day | Intra-day | Inter-day | Intra-day | Inter-day | Intra-day | Inter-day | Intra-day | Inter-day | Intra-day | Inter-day | Intra-day |
| Brazil | 0.00 (-0.13, 0.14) | 0.31 (0.13, 0.49) | 0.01 (-0.08, 0.11) | 0.38 (0.23, 0.54) | 0.18 (0.05, 0.31) | 0.37 (0.22, 0.53) | 0.29 (0.15, 0.43) | 0.49 (0.23, 0.74) | 0.30 (0.12, 0.48) | 0.62 (0.32, 0.93) | 0.29 (0.12, 0.45) | 0.69 (0.38, 0.99) | 0.29 (0.15, 0.43) | 0.83 (0.52, 1.14) |
| Canada | 0.05 (-0.06, 0.16) | 0.25 (0.10, 0.40) | 0.02 (-0.06, 0.09) | 0.36 (0.23, 0.48) | 0.17 (0.06, 0.28) | 0.37 (0.24, 0.49) | 0.29 (0.17, 0.40) | 0.44 (0.23, 0.66) | 0.32 (0.17, 0.47) | 0.52 (0.26, 0.78) | 0.31 (0.18, 0.45) | 0.55 (0.28, 0.81) | 0.31 (0.20, 0.43) | 0.63 (0.36, 0.90) |
| China | 0.03 (-0.12, 0.17) | 0.31 (0.12, 0.51) | 0.01 (-0.09, 0.11) | 0.40 (0.23, 0.57) | 0.17 (0.03, 0.31) | 0.39 (0.22, 0.56) | 0.30 (0.14, 0.45) | 0.51 (0.23, 0.79) | 0.33 (0.13, 0.53) | 0.64 (0.29, 0.99) | 0.32 (0.14, 0.50) | 0.69 (0.34, 1.04) | 0.33 (0.17, 0.48) | 0.79 (0.43, 1.14) |
| Colombia | 0.03 (-0.22, 0.27) | 0.29 (-0.05, 0.62) | 0.02 (-0.15, 0.19) | 0.38 (0.10, 0.67) | 0.16 (-0.07, 0.40) | 0.36 (0.07, 0.65) | 0.27 (0.01, 0.53) | 0.32 (-0.15, 0.79) | 0.27 (-0.05, 0.60) | 0.24 (-0.34, 0.81) | 0.25 (-0.05, 0.55) | 0.23 (-0.35, 0.80) | 0.28 (0.02, 0.54) | 0.30 (-0.29, 0.89) |
| Costa Rica | -0.02 (-0.60, 0.55) | 0.27 (-0.51, 1.04) | 0.01 (-0.38, 0.40) | 0.33 (-0.33, 0.98) | 0.18 (-0.37, 0.74) | 0.31 (-0.36, 0.97) | 0.32 (-0.28, 0.93) | 0.24 (-0.88, 1.36) | 0.32 (-0.46, 1.10) | 0.23 (-1.19, 1.62) | 0.33 (-0.38, 1.03) | 0.29 (-1.12, 1.68) | 0.32 (-0.28, 0.92) | 0.41 (-1.03, 1.83) |
| Czech Republic | 0.05 (-0.23, 0.34) | 0.18 (-0.21, 0.56) | 0.02 (-0.17, 0.22) | 0.31 (-0.02, 0.64) | 0.16 (-0.12, 0.43) | 0.32 (-0.01, 0.65) | 0.29 (-0.01, 0.59) | 0.35 (-0.21, 0.90) | 0.33 (-0.05, 0.71) | 0.38 (-0.32, 1.07) | 0.31 (-0.03, 0.66) | 0.47 (-0.22, 1.16) | 0.33 (0.03, 0.62) | 0.61 (-0.10, 1.31) |
| Ecuador | 0.04 (-0.36, 0.44) | 0.31 (-0.23, 0.86) | 0.03 (-0.25, 0.30) | 0.39 (-0.08, 0.85) | 0.17 (-0.22, 0.55) | 0.36 (-0.11, 0.83) | 0.26 (-0.16, 0.69) | 0.44 (-0.35, 1.22) | 0.26 (-0.28, 0.81) | 0.50 (-0.49, 1.48) | 0.25 (-0.24, 0.75) | 0.46 (-0.53, 1.44) | 0.28 (-0.14, 0.70) | 0.59 (-0.41, 1.59) |
| Estonia | 0.06 (-0.20, 0.31) | 0.25 (-0.10, 0.59) | 0.02 (-0.15, 0.20) | 0.35 (0.05, 0.64) | 0.18 (-0.07, 0.43) | 0.35 (0.05, 0.64) | 0.30 (0.03, 0.57) | 0.38 (-0.13, 0.89) | 0.33 (-0.02, 0.68) | 0.46 (-0.18, 1.10) | 0.32 (-0.00, 0.64) | 0.54 (-0.10, 1.18) | 0.32 (0.05, 0.59) | 0.65 (0.00, 1.30) |
| Finland | 0.04 (-0.52, 0.60) | 0.28 (-0.49, 1.05) | 0.02 (-0.37, 0.40) | 0.38 (-0.27, 1.04) | 0.24 (-0.31, 0.78) | 0.37 (-0.30, 1.03) | 0.41 (-0.19, 1.00) | 0.40 (-0.71, 1.50) | 0.42 (-0.35, 1.18) | 0.64 (-0.75, 2.01) | 0.34 (-0.35, 1.03) | 0.69 (-0.70, 2.07) | 0.34 (-0.25, 0.93) | 0.77 (-0.65, 2.17) |
| France | 0.03 (-0.10, 0.17) | 0.26 (0.07, 0.44) | 0.02 (-0.08, 0.11) | 0.35 (0.19, 0.50) | 0.16 (0.03, 0.29) | 0.35 (0.19, 0.50) | 0.28 (0.14, 0.42) | 0.38 (0.11, 0.64) | 0.30 (0.12, 0.48) | 0.45 (0.12, 0.78) | 0.29 (0.13, 0.46) | 0.53 (0.20, 0.86) | 0.30 (0.16, 0.44) | 0.65 (0.31, 0.99) |
| Greece | 0.10 (-0.43, 0.63) | 0.39 (-0.36, 1.13) | 0.07 (-0.31, 0.45) | 0.44 (-0.20, 1.08) | 0.19 (-0.33, 0.71) | 0.45 (-0.20, 1.10) | 0.35 (-0.22, 0.91) | 0.75 (-0.32, 1.81) | 0.31 (-0.40, 1.03) | 1.09 (-0.22, 2.38) | 0.28 (-0.38, 0.94) | 1.14 (-0.18, 2.44) | 0.24 (-0.33, 0.81) | 1.32 (-0.02, 2.65) |
| Iran | -0.05 (-0.43, 0.32) | 0.13 (-0.39, 0.65) | 0.00 (-0.26, 0.27) | 0.28 (-0.17, 0.73) | 0.23 (-0.15, 0.60) | 0.24 (-0.22, 0.69) | 0.41 (0.00, 0.82) | 0.07 (-0.68, 0.81) | 0.56 (0.04, 1.06) | -0.15 (-1.06, 0.76) | 0.49 (0.02, 0.96) | -0.12 (-1.04, 0.79) | 0.39 (-0.02, 0.79) | 0.01 (-0.93, 0.94) |
| Ireland | -0.02 (-0.25, 0.20) | 0.28 (-0.02, 0.57) | -0.01 (-0.16, 0.15) | 0.32 (0.06, 0.57) | 0.10 (-0.12, 0.32) | 0.30 (0.03, 0.56) | 0.20 (-0.04, 0.43) | 0.24 (-0.17, 0.65) | 0.21 (-0.09, 0.50) | 0.26 (-0.28, 0.80) | 0.27 (-0.00, 0.54) | 0.28 (-0.28, 0.83) | 0.32 (0.08, 0.55) | 0.34 (-0.19, 0.87) |
| Japan | 0.15 (0.08, 0.22) | 0.16 (0.07, 0.25) | 0.06 (0.01, 0.12) | 0.35 (0.26, 0.43) | 0.23 (0.16, 0.30) | 0.39 (0.31, 0.48) | 0.34 (0.27, 0.42) | 0.55 (0.43, 0.67) | 0.40 (0.31, 0.49) | 0.67 (0.52, 0.81) | 0.35 (0.27, 0.43) | 0.77 (0.62, 0.91) | 0.33 (0.26, 0.41) | 0.92 (0.77, 1.06) |
| Kuwait | 0.07 (-0.50, 0.63) | 0.18 (-0.59, 0.94) | 0.03 (-0.36, 0.41) | 0.31 (-0.35, 0.96) | 0.18 (-0.37, 0.72) | 0.34 (-0.33, 1.00) | 0.22 (-0.38, 0.81) | 0.47 (-0.64, 1.57) | 0.17 (-0.60, 0.93) | 0.58 (-0.81, 1.94) | 0.22 (-0.48, 0.91) | 0.68 (-0.70, 2.04) | 0.27 (-0.32, 0.86) | 0.79 (-0.62, 2.18) |
| Mexico | -0.03 (-0.20, 0.13) | 0.29 (0.05, 0.53) | -0.00 (-0.12, 0.12) | 0.35 (0.15, 0.56) | 0.13 (-0.04, 0.30) | 0.34 (0.13, 0.55) | 0.26 (0.08, 0.44) | 0.36 (0.03, 0.70) | 0.31 (0.08, 0.53) | 0.42 (0.01, 0.84) | 0.31 (0.10, 0.52) | 0.51 (0.09, 0.92) | 0.32 (0.14, 0.50) | 0.62 (0.19, 1.05) |
| Norway | 0.07 (-0.48, 0.61) | 0.13 (-0.62, 0.88) | -0.01 (-0.39, 0.37) | 0.26 (-0.38, 0.91) | 0.13 (-0.41, 0.66) | 0.23 (-0.42, 0.89) | 0.27 (-0.32, 0.85) | 0.22 (-0.85, 1.28) | 0.33 (-0.41, 1.07) | 0.26 (-1.06, 1.56) | 0.26 (-0.42, 0.94) | 0.29 (-1.03, 1.60) | 0.29 (-0.29, 0.87) | 0.45 (-0.90, 1.79) |
| Panama | 0.04 (-0.54, 0.61) | 0.21 (-0.57, 0.99) | 0.02 (-0.37, 0.41) | 0.32 (-0.35, 0.98) | 0.17 (-0.38, 0.73) | 0.32 (-0.35, 0.99) | 0.29 (-0.32, 0.90) | 0.35 (-0.79, 1.48) | 0.33 (-0.46, 1.11) | 0.40 (-1.04, 1.82) | 0.30 (-0.41, 1.01) | 0.49 (-0.95, 1.90) | 0.31 (-0.29, 0.91) | 0.59 (-0.88, 2.03) |
| Paraguay | -0.04 (-0.61, 0.53) | 0.21 (-0.56, 0.98) | -0.03 (-0.41, 0.36) | 0.31 (-0.35, 0.97) | 0.10 (-0.45, 0.65) | 0.32 (-0.34, 0.99) | 0.19 (-0.41, 0.80) | 0.31 (-0.81, 1.41) | 0.12 (-0.66, 0.90) | 0.37 (-1.03, 1.74) | 0.13 (-0.58, 0.84) | 0.43 (-0.96, 1.80) | 0.20 (-0.40, 0.80) | 0.58 (-0.84, 1.97) |
| Philippines | 0.07 (-0.09, 0.22) | 0.24 (0.03, 0.46) | 0.03 (-0.07, 0.14) | 0.32 (0.13, 0.50) | 0.20 (0.05, 0.35) | 0.31 (0.13, 0.49) | 0.29 (0.12, 0.45) | 0.30 (-0.01, 0.60) | 0.32 (0.11, 0.53) | 0.38 (-0.00, 0.77) | 0.31 (0.12, 0.50) | 0.50 (0.11, 0.88) | 0.32 (0.15, 0.48) | 0.62 (0.23, 1.01) |
| Portugal | 0.03 (-0.19, 0.26) | 0.12 (-0.18, 0.42) | 0.01 (-0.14, 0.17) | 0.28 (0.02, 0.54) | 0.13 (-0.09, 0.34) | 0.31 (0.04, 0.57) | 0.24 (0.01, 0.47) | 0.33 (-0.10, 0.75) | 0.24 (-0.05, 0.54) | 0.39 (-0.14, 0.90) | 0.25 (-0.02, 0.53) | 0.50 (-0.02, 1.02) | 0.28 (0.05, 0.52) | 0.59 (0.06, 1.13) |
| South Africa | 0.02 (-0.06, 0.09) | 0.28 (0.17, 0.38) | 0.01 (-0.04, 0.07) | 0.33 (0.24, 0.42) | 0.18 (0.11, 0.26) | 0.32 (0.23, 0.41) | 0.31 (0.23, 0.39) | 0.30 (0.15, 0.45) | 0.35 (0.25, 0.45) | 0.33 (0.15, 0.52) | 0.33 (0.24, 0.43) | 0.43 (0.24, 0.61) | 0.32 (0.24, 0.40) | 0.55 (0.36, 0.73) |
| South Korea | 0.04 (-0.05, 0.14) | 0.25 (0.12, 0.38) | 0.02 (-0.04, 0.09) | 0.35 (0.24, 0.46) | 0.18 (0.09, 0.27) | 0.35 (0.24, 0.46) | 0.30 (0.20, 0.40) | 0.40 (0.21, 0.58) | 0.32 (0.19, 0.45) | 0.48 (0.25, 0.71) | 0.31 (0.19, 0.43) | 0.54 (0.31, 0.78) | 0.31 (0.21, 0.41) | 0.66 (0.42, 0.89) |
| Spain | 0.05 (-0.03, 0.13) | 0.24 (0.14, 0.35) | 0.02 (-0.03, 0.07) | 0.35 (0.26, 0.44) | 0.17 (0.10, 0.25) | 0.35 (0.26, 0.44) | 0.29 (0.21, 0.37) | 0.41 (0.25, 0.56) | 0.30 (0.20, 0.41) | 0.51 (0.32, 0.70) | 0.31 (0.21, 0.40) | 0.58 (0.39, 0.77) | 0.31 (0.23, 0.39) | 0.69 (0.50, 0.89) |
| Sweden | 0.04 (-0.28, 0.36) | 0.22 (-0.22, 0.66) | 0.04 (-0.18, 0.26) | 0.34 (-0.04, 0.71) | 0.19 (-0.12, 0.49) | 0.33 (-0.05, 0.71) | 0.33 (-0.01, 0.67) | 0.30 (-0.34, 0.93) | 0.35 (-0.08, 0.78) | 0.28 (-0.51, 1.07) | 0.34 (-0.05, 0.73) | 0.40 (-0.40, 1.19) | 0.32 (-0.02, 0.65) | 0.55 (-0.27, 1.35) |
| Switzerland | 0.05 (-0.16, 0.25) | 0.25 (-0.03, 0.52) | 0.02 (-0.12, 0.16) | 0.34 (0.11, 0.57) | 0.17 (-0.03, 0.37) | 0.34 (0.10, 0.57) | 0.29 (0.07, 0.50) | 0.36 (-0.04, 0.76) | 0.29 (0.02, 0.57) | 0.42 (-0.08, 0.92) | 0.29 (0.04, 0.54) | 0.49 (-0.01, 0.99) | 0.30 (0.09, 0.52) | 0.61 (0.09, 1.12) |
| Taiwan | -0.05 (-0.36, 0.25) | 0.20 (-0.22, 0.62) | -0.03 (-0.25, 0.19) | 0.29 (-0.07, 0.65) | 0.08 (-0.23, 0.39) | 0.34 (-0.03, 0.71) | 0.23 (-0.11, 0.56) | 0.39 (-0.20, 0.98) | 0.19 (-0.23, 0.61) | 0.47 (-0.23, 1.17) | 0.26 (-0.13, 0.65) | 0.51 (-0.21, 1.22) | 0.31 (-0.02, 0.65) | 0.60 (-0.13, 1.32) |
| Thailand | 0.04 (-0.03, 0.11) | 0.26 (0.16, 0.36) | 0.02 (-0.03, 0.07) | 0.35 (0.27, 0.44) | 0.17 (0.10, 0.24) | 0.35 (0.26, 0.43) | 0.29 (0.21, 0.37) | 0.40 (0.25, 0.54) | 0.31 (0.21, 0.41) | 0.47 (0.29, 0.65) | 0.30 (0.21, 0.39) | 0.55 (0.37, 0.73) | 0.31 (0.23, 0.38) | 0.66 (0.48, 0.84) |
| UK | 0.04 (-0.03, 0.11) | 0.25 (0.15, 0.34) | 0.01 (-0.03, 0.06) | 0.35 (0.27, 0.42) | 0.16 (0.10, 0.22) | 0.33 (0.26, 0.41) | 0.28 (0.21, 0.35) | 0.36 (0.23, 0.48) | 0.29 (0.20, 0.38) | 0.41 (0.26, 0.57) | 0.28 (0.20, 0.37) | 0.49 (0.33, 0.65) | 0.30 (0.23, 0.37) | 0.60 (0.43, 0.76) |
| USA | 0.05 (0.01, 0.08) | 0.26 (0.21, 0.31) | 0.02 (-0.01, 0.05) | 0.35 (0.31, 0.40) | 0.18 (0.14, 0.21) | 0.35 (0.30, 0.39) | 0.30 (0.26, 0.34) | 0.39 (0.32, 0.47) | 0.33 (0.28, 0.38) | 0.47 (0.37, 0.56) | 0.32 (0.27, 0.36) | 0.53 (0.44, 0.62) | 0.32 (0.28, 0.36) | 0.63 (0.54, 0.72) |
| Vietnam | 0.02 (-0.39, 0.42) | 0.24 (-0.31, 0.78) | 0.01 (-0.26, 0.29) | 0.34 (-0.13, 0.80) | 0.14 (-0.25, 0.53) | 0.35 (-0.12, 0.81) | 0.27 (-0.16, 0.70) | 0.44 (-0.35, 1.22) | 0.28 (-0.27, 0.83) | 0.55 (-0.43, 1.52) | 0.28 (-0.22, 0.78) | 0.61 (-0.37, 1.58) | 0.31 (-0.11, 0.73) | 0.76 (-0.24, 1.75) |

## Table S8. Percentage change (%) of all-cause mortality risk by climate areas per IQR increase in TV.

|  |  | Cold area | Moderate cold | Moderate warm | Warm area |
| --- | --- | --- | --- | --- | --- |
| TV 0–1 | Inter-day | 0.02 (-0.04, 0.08) | -0.01 (-0.07, 0.05) | -0.02 (-0.07, 0.03) | -0.09 (-0.16, -0.03) |
|  | Intra-day | 0.38 (0.28, 0.48) | 0.57 (0.49, 0.65) | 0.56 (0.47, 0.66) | 0.96 (0.80, 1.12) |
| TV 0–2 | Inter-day | 0.00 (-0.07, 0.07) | -0.06 (-0.12, 0.00) | -0.02 (-0.07, 0.03) | -0.08 (-0.16, -0.01) |
|  | Intra-day | 0.42 (0.33, 0.52) | 0.62 (0.54, 0.70) | 0.60 (0.53, 0.68) | 0.75 (0.61, 0.88) |
| TV 0–3 | Inter-day | 0.08 (0.01, 0.14) | 0.06 (-0.01, 0.12) | 0.10 (0.04, 0.16) | 0.04 (-0.03, 0.11) |
|  | Intra-day | 0.36 (0.26, 0.46) | 0.57 (0.49, 0.65) | 0.59 (0.51, 0.67) | 0.60 (0.46, 0.74) |
| TV 0–4 | Inter-day | 0.17 (0.10, 0.23) | 0.17 (0.10, 0.23) | 0.23 (0.16, 0.30) | 0.08 (0.01, 0.16) |
|  | Intra-day | 0.31 (0.20, 0.41) | 0.56 (0.48, 0.63) | 0.59 (0.50, 0.67) | 0.53 (0.38, 0.68) |
| TV 0–5 | Inter-day | 0.19 (0.12, 0.26) | 0.21 (0.14, 0.29) | 0.31 (0.24, 0.37) | 0.09 (0.00, 0.17) |
|  | Intra-day | 0.32 (0.21, 0.43) | 0.55 (0.47, 0.63) | 0.60 (0.51, 0.69) | 0.54 (0.39, 0.70) |
| TV 0–6 | Inter-day | 0.21 (0.13, 0.28) | 0.22 (0.14, 0.30) | 0.29 (0.23, 0.36) | 0.07 (-0.02, 0.17) |
|  | Intra-day | 0.35 (0.23, 0.46) | 0.59 (0.51, 0.67) | 0.64 (0.55, 0.73) | 0.54 (0.38, 0.70) |
| TV 0–7 | Inter-day | 0.23 (0.16, 0.31) | 0.24 (0.16, 0.32) | 0.29 (0.22, 0.36) | 0.04 (-0.06, 0.14) |
|  | Intra-day | 0.39 (0.27, 0.51) | 0.64 (0.55, 0.72) | 0.71 (0.62, 0.80) | 0.56 (0.40, 0.73) |

## Table S9. Percentage change (%) of cardiovascular mortality risk by climate areas per IQR increase in TV.

|  |  | Cold area | Moderate cold | Moderate warm | Warm area |
| --- | --- | --- | --- | --- | --- |
| TV 0–1 | Inter-day | 0.09 (0.01, 0.17) | 0.08 (-0.02, 0.18) | 0.08 (-0.01, 0.16) | 0.09 (0.00, 0.17) |
|  | Intra-day | 0.56 (0.43, 0.69) | 0.59 (0.45, 0.72) | 0.43 (0.28, 0.57) | 0.49 (0.34, 0.65) |
| TV 0–2 | Inter-day | 0.11 (0.02, 0.20) | 0.08 (-0.03, 0.19) | 0.08 (-0.02, 0.19) | 0.13 (0.03, 0.24) |
|  | Intra-day | 0.73 (0.61, 0.85) | 0.65 (0.53, 0.78) | 0.52 (0.37, 0.66) | 0.49 (0.33, 0.65) |
| TV 0–3 | Inter-day | 0.25 (0.16, 0.34) | 0.25 (0.14, 0.37) | 0.17 (0.06, 0.28) | 0.21 (0.09, 0.33) |
|  | Intra-day | 0.71 (0.58, 0.84) | 0.63 (0.50, 0.77) | 0.57 (0.42, 0.73) | 0.51 (0.35, 0.68) |
| TV 0–4 | Inter-day | 0.34 (0.23, 0.44) | 0.39 (0.27, 0.52) | 0.28 (0.16, 0.40) | 0.28 (0.15, 0.42) |
|  | Intra-day | 0.69 (0.56, 0.83) | 0.59 (0.46, 0.73) | 0.59 (0.43, 0.76) | 0.52 (0.35, 0.70) |
| TV 0–5 | Inter-day | 0.45 (0.35, 0.55) | 0.49 (0.36, 0.61) | 0.36 (0.23, 0.48) | 0.32 (0.17, 0.46) |
|  | Intra-day | 0.67 (0.53, 0.82) | 0.56 (0.42, 0.70) | 0.59 (0.42, 0.76) | 0.53 (0.35, 0.70) |
| TV 0–6 | Inter-day | 0.46 (0.35, 0.57) | 0.53 (0.41, 0.65) | 0.36 (0.23, 0.50) | 0.30 (0.16, 0.45) |
|  | Intra-day | 0.71 (0.56, 0.86) | 0.54 (0.37, 0.70) | 0.63 (0.45, 0.80) | 0.58 (0.41, 0.75) |
| TV 0–7 | Inter-day | 0.48 (0.36, 0.59) | 0.57 (0.45, 0.70) | 0.38 (0.25, 0.52) | 0.32 (0.18, 0.47) |
|  | Intra-day | 0.75 (0.59, 0.91) | 0.52 (0.35, 0.70) | 0.66 (0.48, 0.84) | 0.62 (0.45, 0.80) |

## Table S10. Percentage change (%) of respiratory mortality risk by climate areas per IQR increase in TV.

|  |  | Cold area | Moderate cold | Moderate warm | Warm area |
| --- | --- | --- | --- | --- | --- |
| TV 0–1 | Inter-day | 0.14 (-0.03, 0.32) | 0.06 (-0.09, 0.22) | -0.02 (-0.17, 0.13) | 0.02 (-0.16, 0.19) |
|  | Intra-day | 0.28 (-0.00, 0.56) | 0.22 (0.01, 0.44) | 0.24 (0.07, 0.41) | 0.29 (0.06, 0.51) |
| TV 0–2 | Inter-day | 0.09 (-0.10, 0.28) | -0.03 (-0.20, 0.14) | -0.03 (-0.16, 0.11) | 0.05 (-0.13, 0.23) |
|  | Intra-day | 0.29 (0.02, 0.55) | 0.43 (0.20, 0.66) | 0.43 (0.25, 0.62) | 0.24 (0.00, 0.48) |
| TV 0–3 | Inter-day | 0.25 (0.04, 0.47) | 0.19 (0.01, 0.36) | 0.13 (-0.03, 0.30) | 0.13 (-0.07, 0.33) |
|  | Intra-day | 0.28 (0.03, 0.54) | 0.35 (0.11, 0.59) | 0.45 (0.25, 0.66) | 0.23 (-0.04, 0.50) |
| TV 0–4 | Inter-day | 0.36 (0.15, 0.56) | 0.35 (0.16, 0.54) | 0.24 (0.06, 0.42) | 0.27 (0.08, 0.45) |
|  | Intra-day | 0.40 (0.09, 0.71) | 0.39 (0.14, 0.64) | 0.44 (0.19, 0.69) | 0.27 (-0.04, 0.58) |
| TV 0–5 | Inter-day | 0.33 (0.09, 0.57) | 0.39 (0.18, 0.59) | 0.29 (0.10, 0.47) | 0.32 (0.15, 0.50) |
|  | Intra-day | 0.55 (0.23, 0.86) | 0.43 (0.15, 0.71) | 0.47 (0.19, 0.75) | 0.36 (0.02, 0.70) |
| TV 0–6 | Inter-day | 0.32 (0.07, 0.57) | 0.35 (0.16, 0.55) | 0.26 (0.06, 0.45) | 0.31 (0.13, 0.49) |
|  | Intra-day | 0.69 (0.38, 1.00) | 0.45 (0.19, 0.72) | 0.54 (0.23, 0.84) | 0.42 (0.07, 0.76) |
| TV 0–7 | Inter-day | 0.36 (0.11, 0.60) | 0.29 (0.09, 0.49) | 0.30 (0.12, 0.49) | 0.28 (0.10, 0.46) |
|  | Intra-day | 0.88 (0.57, 1.19) | 0.53 (0.25, 0.80) | 0.64 (0.32, 0.95) | 0.49 (0.14, 0.83) |

## Table S11. Percentage change (%) of all-cause mortality risk by seasons per IQR increase in TV.

|  |  | Cold season | Moderate season | Warm season |
| --- | --- | --- | --- | --- |
| TV 0–1 | Inter-day | 0.01 (-0.04, 0.05) | 0.00 (-0.04, 0.05) | -0.02 (-0.07, 0.03) |
|  | Intra-day | 0.31 (0.24, 0.38) | 0.64 (0.56, 0.72) | 0.60 (0.50, 0.70) |
| TV 0–2 | Inter-day | -0.00 (-0.06, 0.05) | -0.03 (-0.08, 0.02) | -0.15 (-0.20, -0.09) |
|  | Intra-day | 0.28 (0.21, 0.35) | 0.67 (0.59, 0.74) | 0.59 (0.50, 0.68) |
| TV 0–3 | Inter-day | 0.11 (0.06, 0.17) | 0.09 (0.04, 0.15) | -0.10 (-0.16, -0.04) |
|  | Intra-day | 0.15 (0.07, 0.23) | 0.62 (0.55, 0.69) | 0.54 (0.46, 0.63) |
| TV 0–4 | Inter-day | 0.21 (0.15, 0.27) | 0.24 (0.18, 0.29) | -0.03 (-0.09, 0.04) |
|  | Intra-day | 0.08 (0.00, 0.17) | 0.57 (0.49, 0.65) | 0.52 (0.43, 0.61) |
| TV 0–5 | Inter-day | 0.23 (0.17, 0.29) | 0.33 (0.27, 0.40) | -0.01 (-0.08, 0.06) |
|  | Intra-day | 0.08 (-0.01, 0.16) | 0.56 (0.49, 0.64) | 0.54 (0.45, 0.63) |
| TV 0–6 | Inter-day | 0.18 (0.12, 0.25) | 0.36 (0.29, 0.42) | 0.01 (-0.07, 0.08) |
|  | Intra-day | 0.08 (-0.01, 0.17) | 0.61 (0.53, 0.69) | 0.60 (0.50, 0.69) |
| TV 0–7 | Inter-day | 0.17 (0.10, 0.24) | 0.35 (0.28, 0.42) | 0.02 (-0.06, 0.10) |
|  | Intra-day | 0.13 (0.04, 0.22) | 0.70 (0.62, 0.79) | 0.67 (0.57, 0.78) |

## Table S12. Percentage change (%) of cardiovascular mortality risk by seasons per IQR increase in TV.

|  |  | Cold season | Moderate season | Warm season |
| --- | --- | --- | --- | --- |
| TV 0–1 | Inter-day | 0.11 (0.04, 0.18) | 0.05 (-0.03, 0.12) | 0.07 (-0.01, 0.16) |
|  | Intra-day | 0.30 (0.19, 0.41) | 0.58 (0.47, 0.70) | 0.40 (0.30, 0.51) |
| TV 0–2 | Inter-day | 0.12 (0.04, 0.21) | 0.07 (-0.01, 0.16) | -0.07 (-0.15, 0.02) |
|  | Intra-day | 0.30 (0.18, 0.42) | 0.78 (0.66, 0.90) | 0.49 (0.36, 0.62) |
| TV 0–3 | Inter-day | 0.26 (0.17, 0.35) | 0.19 (0.10, 0.28) | -0.05 (-0.15, 0.04) |
|  | Intra-day | 0.21 (0.07, 0.34) | 0.82 (0.69, 0.94) | 0.53 (0.39, 0.66) |
| TV 0–4 | Inter-day | 0.32 (0.22, 0.43) | 0.32 (0.23, 0.42) | 0.04 (-0.07, 0.15) |
|  | Intra-day | 0.18 (0.03, 0.32) | 0.80 (0.67, 0.93) | 0.50 (0.37, 0.64) |
| TV 0–5 | Inter-day | 0.35 (0.24, 0.46) | 0.49 (0.39, 0.59) | 0.13 (0.01, 0.24) |
|  | Intra-day | 0.17 (0.02, 0.31) | 0.73 (0.59, 0.87) | 0.52 (0.37, 0.67) |
| TV 0–6 | Inter-day | 0.30 (0.18, 0.41) | 0.57 (0.47, 0.68) | 0.17 (0.04, 0.29) |
|  | Intra-day | 0.19 (0.04, 0.34) | 0.74 (0.58, 0.89) | 0.60 (0.44, 0.75) |
| TV 0–7 | Inter-day | 0.28 (0.16, 0.39) | 0.57 (0.45, 0.68) | 0.22 (0.08, 0.35) |
|  | Intra-day | 0.21 (0.05, 0.36) | 0.80 (0.65, 0.94) | 0.67 (0.50, 0.84) |

## Table S13. Percentage change (%) of respiratory mortality risk by seasons per IQR increase in TV.

|  |  | Cold season | Moderate season | Warm season |
| --- | --- | --- | --- | --- |
| TV 0–1 | Inter-day | -0.03 (-0.15, 0.10) | 0.18 (0.04, 0.32) | 0.15 (0.01, 0.30) |
|  | Intra-day | 0.11 (-0.06, 0.27) | 0.23 (0.04, 0.43) | 0.36 (0.15, 0.56) |
| TV 0–2 | Inter-day | 0.00 (-0.13, 0.13) | 0.01 (-0.13, 0.15) | 0.06 (-0.10, 0.21) |
|  | Intra-day | 0.10 (-0.08, 0.27) | 0.37 (0.17, 0.58) | 0.53 (0.32, 0.73) |
| TV 0–3 | Inter-day | 0.15 (-0.00, 0.31) | 0.15 (-0.00, 0.31) | 0.10 (-0.07, 0.28) |
|  | Intra-day | -0.02 (-0.20, 0.16) | 0.39 (0.19, 0.59) | 0.51 (0.30, 0.72) |
| TV 0–4 | Inter-day | 0.29 (0.13, 0.46) | 0.30 (0.14, 0.46) | 0.16 (-0.02, 0.35) |
|  | Intra-day | -0.07 (-0.27, 0.12) | 0.50 (0.29, 0.71) | 0.60 (0.37, 0.82) |
| TV 0–5 | Inter-day | 0.30 (0.12, 0.48) | 0.45 (0.28, 0.62) | 0.12 (-0.08, 0.32) |
|  | Intra-day | -0.04 (-0.25, 0.17) | 0.48 (0.25, 0.71) | 0.69 (0.44, 0.94) |
| TV 0–6 | Inter-day | 0.22 (0.02, 0.41) | 0.51 (0.32, 0.69) | 0.08 (-0.12, 0.29) |
|  | Intra-day | -0.02 (-0.24, 0.20) | 0.55 (0.31, 0.80) | 0.77 (0.50, 1.04) |
| TV 0–7 | Inter-day | 0.17 (-0.03, 0.36) | 0.54 (0.34, 0.73) | 0.10 (-0.11, 0.31) |
|  | Intra-day | 0.06 (-0.18, 0.30) | 0.66 (0.40, 0.93) | 0.98 (0.70, 1.26) |

## Table S14. Percentage change (%) of all-cause mortality risk by Köppen-Geiger climate groups per IQR increase in TV.

|  |  | Tropical | Dry | Temperate | Continental | Polar |
| --- | --- | --- | --- | --- | --- | --- |
| TV 0–1 | Inter-day | -0.07 (-0.18, 0.03) | -0.24 (-0.36, -0.12) | -0.00 (-0.03, 0.03) | -0.00 (-0.08, 0.07) | 0.26 (-0.77, 1.27) |
|  | Intra-day | 1.20 (0.88, 1.52) | 0.92 (0.65, 1.18) | 0.54 (0.48, 0.59) | 0.47 (0.35, 0.59) | 0.20 (-1.68, 2.05) |
| TV 0–2 | Inter-day | -0.04 (-0.15, 0.07) | -0.15 (-0.27, -0.03) | -0.03 (-0.07, 0.01) | -0.01 (-0.09, 0.08) | -0.11 (-1.28, 1.05) |
|  | Intra-day | 0.68 (0.43, 0.93) | 0.76 (0.52, 0.99) | 0.58 (0.53, 0.64) | 0.54 (0.42, 0.65) | 0.56 (-1.49, 2.56) |
| TV 0–3 | Inter-day | 0.04 (-0.06, 0.15) | 0.05 (-0.07, 0.17) | 0.08 (0.04, 0.12) | 0.11 (0.03, 0.20) | -0.17 (-1.10, 0.74) |
|  | Intra-day | 0.44 (0.16, 0.72) | 0.61 (0.37, 0.86) | 0.55 (0.50, 0.60) | 0.49 (0.37, 0.61) | 0.94 (-1.25, 3.09) |
| TV 0–4 | Inter-day | 0.01 (-0.10, 0.13) | 0.19 (0.06, 0.31) | 0.18 (0.14, 0.23) | 0.23 (0.14, 0.32) | -0.18 (-1.69, 1.32) |
|  | Intra-day | 0.36 (0.07, 0.66) | 0.56 (0.32, 0.81) | 0.52 (0.46, 0.58) | 0.46 (0.34, 0.59) | 0.74 (-1.84, 3.27) |
| TV 0–5 | Inter-day | -0.00 (-0.12, 0.12) | 0.26 (0.11, 0.40) | 0.23 (0.19, 0.28) | 0.27 (0.19, 0.36) | -0.13 (-2.71, 2.39) |
|  | Intra-day | 0.35 (0.05, 0.64) | 0.59 (0.35, 0.83) | 0.52 (0.46, 0.58) | 0.49 (0.37, 0.61) | 0.85 (-1.59, 3.24) |
| TV 0–6 | Inter-day | -0.02 (-0.15, 0.12) | 0.21 (0.06, 0.37) | 0.23 (0.19, 0.28) | 0.28 (0.18, 0.37) | 0.19 (-1.64, 1.98) |
|  | Intra-day | 0.26 (-0.03, 0.55) | 0.70 (0.47, 0.94) | 0.56 (0.49, 0.62) | 0.51 (0.39, 0.63) | 0.17 (-2.46, 2.72) |
| TV 0–7 | Inter-day | -0.05 (-0.18, 0.09) | 0.17 (0.01, 0.33) | 0.24 (0.20, 0.29) | 0.29 (0.19, 0.39) | 0.42 (-0.69, 1.52) |
|  | Intra-day | 0.26 (-0.06, 0.58) | 0.82 (0.54, 1.10) | 0.61 (0.54, 0.68) | 0.54 (0.42, 0.67) | -0.39 (-3.17, 2.31) |

## Table S15. Percentage change (%) of cardiovascular mortality risk by Köppen-Geiger climate groups per IQR increase in TV.

|  |  | Tropical | Dry | Temperate | Continental | Polar |
| --- | --- | --- | --- | --- | --- | --- |
| TV 0–1 | Inter-day | 0.10 (-0.02, 0.21) | 0.01 (-0.18, 0.19) | 0.11 (0.05, 0.16) | 0.00 (-0.11, 0.12) | ­– |
|  | Intra-day | 0.56 (0.38, 0.74) | 0.58 (0.23, 0.94) | 0.52 (0.43, 0.61) | 0.50 (0.37, 0.64) | – |
| TV 0–2 | Inter-day | 0.16 (0.02, 0.30) | -0.05 (-0.24, 0.14) | 0.12 (0.06, 0.19) | 0.02 (-0.11, 0.15) | – |
|  | Intra-day | 0.65 (0.45, 0.85) | 0.58 (0.29, 0.88) | 0.60 (0.51, 0.69) | 0.66 (0.52, 0.80) | – |
| TV 0–3 | Inter-day | 0.18 (0.04, 0.32) | 0.16 (-0.05, 0.36) | 0.23 (0.16, 0.31) | 0.21 (0.09, 0.34) | – |
|  | Intra-day | 0.72 (0.51, 0.93) | 0.39 (0.07, 0.71) | 0.62 (0.52, 0.71) | 0.64 (0.48, 0.80) | – |
| TV 0–4 | Inter-day | 0.26 (0.10, 0.43) | 0.34 (0.12, 0.55) | 0.33 (0.25, 0.41) | 0.32 (0.19, 0.46) | – |
|  | Intra-day | 0.74 (0.53, 0.96) | 0.27 (-0.09, 0.62) | 0.61 (0.51, 0.70) | 0.64 (0.47, 0.81) | – |
| TV 0–5 | Inter-day | 0.35 (0.19, 0.51) | 0.33 (0.11, 0.55) | 0.42 (0.33, 0.50) | 0.41 (0.28, 0.54) | – |
|  | Intra-day | 0.72 (0.49, 0.95) | 0.28 (-0.11, 0.66) | 0.62 (0.52, 0.71) | 0.56 (0.37, 0.74) | – |
| TV 0–6 | Inter-day | 0.37 (0.20, 0.54) | 0.30 (0.07, 0.53) | 0.44 (0.36, 0.52) | 0.39 (0.24, 0.54) | – |
|  | Intra-day | 0.73 (0.48, 0.98) | 0.36 (0.01, 0.72) | 0.65 (0.55, 0.75) | 0.56 (0.37, 0.75) | – |
| TV 0–7 | Inter-day | 0.42 (0.24, 0.61) | 0.38 (0.15, 0.62) | 0.46 (0.38, 0.54) | 0.39 (0.23, 0.55) | – |
|  | Intra-day | 0.74 (0.48, 1.01) | 0.39 (0.00, 0.77) | 0.68 (0.58, 0.78) | 0.58 (0.39, 0.77) | – |

## Table S16. Percentage change (%) of respiratory mortality risk by Köppen-Geiger climate groups per IQR increase in TV.

|  |  | Tropical | Dry | Temperate | Continental | Polar |
| --- | --- | --- | --- | --- | --- | --- |
| TV 0–1 | Inter-day | 0.06 (-0.20, 0.33) | -0.32 (-0.62, -0.03) | 0.07 (-0.03, 0.17) | 0.07 (-0.13, 0.27) | ­– |
|  | Intra-day | 0.38 (-0.12, 0.87) | 0.22 (-0.30, 0.73) | 0.23 (0.10, 0.36) | 0.36 (0.10, 0.61) | – |
| TV 0–2 | Inter-day | 0.17 (-0.13, 0.47) | -0.07 (-0.40, 0.25) | 0.02 (-0.08, 0.12) | -0.05 (-0.26, 0.17) | – |
|  | Intra-day | 0.14 (-0.42, 0.70) | 0.10 (-0.41, 0.61) | 0.34 (0.21, 0.47) | 0.54 (0.26, 0.82) | – |
| TV 0–3 | Inter-day | 0.35 (0.04, 0.67) | 0.21 (-0.15, 0.56) | 0.15 (0.04, 0.26) | 0.20 (-0.03, 0.42) | – |
|  | Intra-day | -0.33 (-1.05, 0.39) | -0.16 (-0.67, 0.36) | 0.37 (0.24, 0.50) | 0.58 (0.28, 0.88) | – |
| TV 0–4 | Inter-day | 0.18 (-0.15, 0.51) | 0.27 (-0.10, 0.64) | 0.31 (0.19, 0.42) | 0.33 (0.10, 0.56) | – |
|  | Intra-day | -0.57 (-1.41, 0.25) | -0.13 (-0.66, 0.40) | 0.42 (0.27, 0.57) | 0.71 (0.42, 1.00) | – |
| TV 0–5 | Inter-day | 0.11 (-0.23, 0.45) | 0.23 (-0.16, 0.61) | 0.36 (0.23, 0.48) | 0.35 (0.11, 0.58) | – |
|  | Intra-day | -0.42 (-1.29, 0.45) | 0.01 (-0.59, 0.60) | 0.48 (0.31, 0.65) | 0.81 (0.45, 1.17) | – |
| TV 0–6 | Inter-day | 0.10 (-0.27, 0.47) | 0.16 (-0.24, 0.55) | 0.35 (0.22, 0.47) | 0.32 (0.07, 0.56) | – |
|  | Intra-day | -0.35 (-1.27, 0.56) | 0.21 (-0.40, 0.82) | 0.56 (0.39, 0.74) | 0.78 (0.46, 1.09) | – |
| TV 0–7 | Inter-day | -0.01 (-0.40, 0.38) | 0.15 (-0.26, 0.56) | 0.35 (0.23, 0.47) | 0.34 (0.09, 0.58) | – |
|  | Intra-day | -0.16 (-1.11, 0.78) | 0.45 (-0.21, 1.10) | 0.68 (0.49, 0.86) | 0.80 (0.46, 1.13) | – |

## Table S17. Attributable fractions (%) of both inter-day TV and intra-day TV for all-cause mortality

| Country/region | TV 0–1 | | TV 0–2 | | TV 0–3 | | TV 0–4 | | TV 0–5 | | TV 0–6 | |
| --- | --- | --- | --- | --- | --- | --- | --- | --- | --- | --- | --- | --- |
|  | Inter-day | Intra-day | Inter-day | Intra-day | Inter-day | Intra-day | Inter-day | Intra-day | Inter-day | Intra-day | Inter-day | Intra-day |
| Argentina | -0.03 (-0.06, 0.00) | 1.25 (1.14, 1.37) | -0.06 (-0.11, -0.02) | 1.44 (1.32, 1.56) | 0.12 (0.07, 0.17) | 1.36 (1.23, 1.49) | 0.31 (0.25, 0.37) | 1.31 (1.17, 1.45) | 0.41 (0.34, 0.48) | 1.33 (1.19, 1.48) | 0.41 (0.34, 0.48) | 1.41 (1.26, 1.56) |
| Australia | -0.02 (-0.05, 0.00) | 0.95 (0.86, 1.03) | -0.05 (-0.09, -0.02) | 1.02 (0.94, 1.11) | 0.10 (0.06, 0.14) | 0.97 (0.88, 1.07) | 0.25 (0.20, 0.30) | 0.96 (0.85, 1.06) | 0.32 (0.27, 0.37) | 1.07 (0.95, 1.18) | 0.33 (0.27, 0.39) | 1.22 (1.09, 1.35) |
| Brazil | -0.02 (-0.04, 0.00) | 1.08 (0.98, 1.18) | -0.04 (-0.06, -0.01) | 1.20 (1.10, 1.30) | 0.07 (0.04, 0.10) | 1.14 (1.03, 1.25) | 0.18 (0.15, 0.22) | 1.14 (1.02, 1.27) | 0.24 (0.20, 0.28) | 1.22 (1.09, 1.36) | 0.25 (0.20, 0.29) | 1.39 (1.24, 1.54) |
| Canada | -0.04 (-0.08, 0.00) | 1.16 (1.05, 1.27) | -0.08 (-0.13, -0.02) | 1.29 (1.19, 1.40) | 0.15 (0.08, 0.21) | 1.25 (1.13, 1.37) | 0.37 (0.30, 0.45) | 1.23 (1.10, 1.36) | 0.49 (0.41, 0.57) | 1.31 (1.17, 1.46) | 0.50 (0.42, 0.58) | 1.46 (1.31, 1.62) |
| Chile | -0.02 (-0.04, 0.00) | 1.35 (1.23, 1.47) | -0.04 (-0.06, -0.01) | 1.49 (1.37, 1.61) | 0.06 (0.04, 0.09) | 1.38 (1.24, 1.51) | 0.16 (0.13, 0.20) | 1.34 (1.20, 1.49) | 0.22 (0.18, 0.25) | 1.36 (1.21, 1.51) | 0.22 (0.19, 0.26) | 1.49 (1.33, 1.64) |
| China | -0.02 (-0.04, 0.00) | 0.88 (0.80, 0.96) | -0.05 (-0.08, -0.01) | 0.98 (0.90, 1.06) | 0.09 (0.05, 0.12) | 0.92 (0.83, 1.01) | 0.22 (0.18, 0.27) | 0.87 (0.77, 0.96) | 0.29 (0.25, 0.34) | 0.91 (0.81, 1.01) | 0.30 (0.25, 0.36) | 1.00 (0.90, 1.11) |
| Colombia | -0.01 (-0.03, 0.00) | 0.90 (0.81, 0.98) | -0.03 (-0.04, -0.01) | 0.88 (0.81, 0.95) | 0.05 (0.03, 0.07) | 0.83 (0.75, 0.91) | 0.11 (0.09, 0.14) | 0.83 (0.74, 0.92) | 0.14 (0.12, 0.16) | 0.91 (0.81, 1.01) | 0.14 (0.12, 0.16) | 1.04 (0.93, 1.15) |
| Costa Rica | -0.01 (-0.02, 0.00) | 0.98 (0.89, 1.07) | -0.02 (-0.04, -0.01) | 1.02 (0.93, 1.10) | 0.04 (0.02, 0.06) | 0.91 (0.82, 1.00) | 0.11 (0.09, 0.13) | 0.92 (0.82, 1.02) | 0.13 (0.11, 0.16) | 1.04 (0.92, 1.15) | 0.12 (0.10, 0.14) | 1.20 (1.07, 1.32) |
| Czech Republic | -0.03 (-0.06, 0.00) | 0.83 (0.75, 0.90) | -0.06 (-0.11, -0.02) | 0.96 (0.88, 1.04) | 0.12 (0.07, 0.17) | 0.93 (0.84, 1.02) | 0.31 (0.25, 0.37) | 0.92 (0.82, 1.02) | 0.41 (0.34, 0.48) | 0.98 (0.87, 1.09) | 0.43 (0.35, 0.50) | 1.09 (0.98, 1.21) |
| Ecuador | -0.01 (-0.03, 0.00) | 1.01 (0.92, 1.11) | -0.03 (-0.05, -0.01) | 0.89 (0.81, 0.96) | 0.05 (0.03, 0.07) | 0.73 (0.66, 0.80) | 0.13 (0.10, 0.15) | 0.73 (0.65, 0.81) | 0.15 (0.12, 0.17) | 0.84 (0.75, 0.93) | 0.13 (0.11, 0.15) | 0.99 (0.89, 1.10) |
| Estonia | -0.03 (-0.06, 0.00) | 1.01 (0.92, 1.10) | -0.06 (-0.10, -0.02) | 1.15 (1.06, 1.25) | 0.11 (0.06, 0.16) | 1.11 (1.00, 1.21) | 0.28 (0.23, 0.34) | 1.09 (0.97, 1.21) | 0.36 (0.30, 0.42) | 1.13 (1.01, 1.25) | 0.38 (0.31, 0.44) | 1.24 (1.10, 1.37) |
| Finland | -0.02 (-0.05, 0.00) | 0.86 (0.78, 0.94) | -0.06 (-0.09, -0.02) | 0.97 (0.89, 1.05) | 0.10 (0.06, 0.15) | 0.93 (0.84, 1.02) | 0.27 (0.22, 0.33) | 0.93 (0.83, 1.03) | 0.37 (0.31, 0.43) | 1.00 (0.89, 1.11) | 0.37 (0.31, 0.44) | 1.13 (1.01, 1.25) |
| France | -0.02 (-0.05, 0.00) | 1.05 (0.96, 1.15) | -0.05 (-0.09, -0.02) | 1.19 (1.09, 1.29) | 0.09 (0.05, 0.14) | 1.13 (1.02, 1.23) | 0.25 (0.20, 0.29) | 1.12 (1.00, 1.24) | 0.32 (0.26, 0.37) | 1.19 (1.06, 1.32) | 0.32 (0.27, 0.38) | 1.34 (1.20, 1.48) |
| French Caribbean | -0.01 (-0.02, 0.00) | 0.70 (0.64, 0.77) | -0.02 (-0.03, -0.01) | 0.80 (0.73, 0.86) | 0.03 (0.02, 0.05) | 0.71 (0.64, 0.78) | 0.08 (0.06, 0.09) | 0.76 (0.68, 0.84) | 0.10 (0.08, 0.11) | 0.85 (0.76, 0.95) | 0.09 (0.08, 0.11) | 0.98 (0.88, 1.09) |
| French Guiana | -0.01 (-0.02, 0.00) | 0.83 (0.75, 0.90) | -0.02 (-0.03, -0.01) | 0.94 (0.86, 1.02) | 0.03 (0.02, 0.04) | 0.97 (0.88, 1.07) | 0.07 (0.06, 0.09) | 1.01 (0.90, 1.12) | 0.09 (0.08, 0.11) | 1.10 (0.98, 1.23) | 0.09 (0.07, 0.10) | 1.25 (1.12, 1.38) |
| Réunion | -0.01 (-0.02, 0.00) | 0.71 (0.65, 0.78) | -0.02 (-0.03, -0.01) | 0.78 (0.72, 0.85) | 0.03 (0.02, 0.05) | 0.75 (0.67, 0.82) | 0.08 (0.07, 0.10) | 0.67 (0.59, 0.74) | 0.10 (0.08, 0.12) | 0.66 (0.59, 0.74) | 0.10 (0.09, 0.12) | 0.74 (0.66, 0.82) |
| Germany | -0.03 (-0.05, 0.00) | 1.09 (0.99, 1.19) | -0.06 (-0.10, -0.02) | 1.22 (1.12, 1.32) | 0.11 (0.06, 0.15) | 1.17 (1.05, 1.28) | 0.28 (0.23, 0.34) | 1.16 (1.03, 1.28) | 0.36 (0.30, 0.42) | 1.23 (1.09, 1.36) | 0.37 (0.31, 0.43) | 1.37 (1.22, 1.51) |
| Greece | -0.02 (-0.04, 0.00) | 1.04 (0.95, 1.14) | -0.04 (-0.07, -0.01) | 1.13 (1.04, 1.23) | 0.08 (0.04, 0.11) | 1.10 (1.00, 1.21) | 0.20 (0.16, 0.24) | 1.08 (0.96, 1.20) | 0.28 (0.23, 0.32) | 1.17 (1.04, 1.30) | 0.29 (0.24, 0.34) | 1.32 (1.18, 1.46) |
| Guatemala | -0.01 (-0.03, 0.00) | 1.05 (0.95, 1.14) | -0.03 (-0.04, -0.01) | 1.15 (1.06, 1.25) | 0.05 (0.03, 0.07) | 1.04 (0.94, 1.14) | 0.13 (0.10, 0.15) | 0.90 (0.80, 1.00) | 0.16 (0.13, 0.19) | 0.97 (0.87, 1.08) | 0.15 (0.12, 0.17) | 1.14 (1.01, 1.26) |
| Iran | -0.02 (-0.05, 0.00) | 1.30 (1.18, 1.42) | -0.05 (-0.09, -0.02) | 1.43 (1.31, 1.55) | 0.10 (0.06, 0.14) | 1.32 (1.19, 1.44) | 0.26 (0.21, 0.31) | 1.35 (1.21, 1.50) | 0.34 (0.29, 0.40) | 1.49 (1.32, 1.65) | 0.36 (0.30, 0.42) | 1.69 (1.51, 1.87) |
| Ireland | -0.02 (-0.04, 0.00) | 0.84 (0.77, 0.92) | -0.04 (-0.07, -0.01) | 0.95 (0.87, 1.03) | 0.08 (0.04, 0.11) | 0.91 (0.83, 1.00) | 0.20 (0.16, 0.24) | 0.91 (0.81, 1.01) | 0.26 (0.22, 0.30) | 0.92 (0.82, 1.02) | 0.27 (0.22, 0.31) | 1.01 (0.91, 1.12) |
| Israel | -0.02 (-0.04, 0.00) | 1.17 (1.07, 1.28) | -0.04 (-0.06, -0.01) | 1.26 (1.16, 1.37) | 0.07 (0.04, 0.10) | 1.19 (1.08, 1.31) | 0.18 (0.14, 0.21) | 1.17 (1.04, 1.30) | 0.24 (0.20, 0.27) | 1.17 (1.04, 1.30) | 0.24 (0.20, 0.28) | 1.20 (1.07, 1.32) |
| Italy | -0.02 (-0.04, 0.00) | 0.98 (0.89, 1.07) | -0.04 (-0.06, -0.01) | 1.10 (1.01, 1.19) | 0.07 (0.04, 0.10) | 1.05 (0.95, 1.15) | 0.18 (0.14, 0.22) | 1.08 (0.97, 1.20) | 0.24 (0.20, 0.28) | 1.19 (1.06, 1.32) | 0.25 (0.21, 0.30) | 1.35 (1.21, 1.49) |
| Japan | -0.02 (-0.05, 0.00) | 1.02 (0.92, 1.11) | -0.05 (-0.08, -0.02) | 1.11 (1.02, 1.20) | 0.09 (0.05, 0.13) | 1.05 (0.95, 1.15) | 0.23 (0.18, 0.27) | 1.03 (0.91, 1.14) | 0.30 (0.25, 0.35) | 1.08 (0.97, 1.20) | 0.30 (0.25, 0.36) | 1.18 (1.06, 1.31) |
| Kuwait | -0.01 (-0.03, 0.00) | 0.90 (0.82, 0.98) | -0.03 (-0.06, -0.01) | 0.99 (0.91, 1.07) | 0.06 (0.04, 0.09) | 0.95 (0.86, 1.04) | 0.16 (0.13, 0.20) | 0.91 (0.81, 1.01) | 0.22 (0.18, 0.26) | 0.92 (0.81, 1.02) | 0.23 (0.19, 0.27) | 1.00 (0.89, 1.10) |
| Mexico | -0.01 (-0.03, 0.00) | 1.51 (1.37, 1.65) | -0.03 (-0.06, -0.01) | 1.63 (1.50, 1.76) | 0.06 (0.03, 0.09) | 1.48 (1.33, 1.62) | 0.16 (0.13, 0.19) | 1.37 (1.22, 1.52) | 0.21 (0.17, 0.24) | 1.37 (1.22, 1.51) | 0.21 (0.18, 0.25) | 1.54 (1.38, 1.70) |
| Moldova | -0.03 (-0.06, 0.00) | 1.16 (1.06, 1.27) | -0.06 (-0.10, -0.02) | 1.28 (1.17, 1.39) | 0.11 (0.06, 0.16) | 1.19 (1.08, 1.31) | 0.29 (0.23, 0.35) | 1.21 (1.08, 1.34) | 0.38 (0.32, 0.45) | 1.31 (1.17, 1.46) | 0.40 (0.33, 0.47) | 1.48 (1.32, 1.64) |
| Netherland | -0.02 (-0.05, 0.00) | 1.00 (0.90, 1.09) | -0.05 (-0.09, -0.02) | 1.12 (1.02, 1.21) | 0.10 (0.05, 0.14) | 1.05 (0.95, 1.15) | 0.25 (0.20, 0.30) | 0.98 (0.87, 1.08) | 0.33 (0.27, 0.38) | 1.05 (0.93, 1.16) | 0.34 (0.28, 0.40) | 1.19 (1.07, 1.32) |
| Norway | -0.03 (-0.05, 0.00) | 0.96 (0.88, 1.05) | -0.06 (-0.09, -0.02) | 1.10 (1.01, 1.19) | 0.11 (0.06, 0.15) | 1.08 (0.98, 1.18) | 0.28 (0.22, 0.33) | 1.08 (0.96, 1.20) | 0.36 (0.30, 0.42) | 1.15 (1.02, 1.27) | 0.38 (0.32, 0.45) | 1.25 (1.12, 1.39) |
| Panama | -0.01 (-0.02, 0.00) | 0.82 (0.74, 0.89) | -0.02 (-0.04, -0.01) | 0.78 (0.72, 0.85) | 0.04 (0.02, 0.06) | 0.77 (0.69, 0.84) | 0.11 (0.09, 0.13) | 0.72 (0.65, 0.80) | 0.14 (0.12, 0.17) | 0.72 (0.64, 0.80) | 0.15 (0.12, 0.17) | 0.80 (0.71, 0.88) |
| Paraguay | -0.03 (-0.06, 0.00) | 1.37 (1.25, 1.50) | -0.07 (-0.12, -0.02) | 1.48 (1.36, 1.60) | 0.13 (0.07, 0.19) | 1.43 (1.30, 1.57) | 0.36 (0.29, 0.43) | 1.40 (1.25, 1.56) | 0.48 (0.40, 0.56) | 1.48 (1.32, 1.65) | 0.51 (0.42, 0.60) | 1.63 (1.46, 1.80) |
| Peru | -0.01 (-0.02, 0.00) | 1.07 (0.97, 1.16) | -0.02 (-0.04, -0.01) | 1.15 (1.06, 1.25) | 0.04 (0.02, 0.05) | 1.09 (0.98, 1.19) | 0.09 (0.07, 0.11) | 1.10 (0.98, 1.22) | 0.12 (0.10, 0.13) | 1.21 (1.08, 1.35) | 0.12 (0.10, 0.14) | 1.38 (1.24, 1.53) |
| Philippines | -0.01 (-0.02, 0.00) | 0.60 (0.55, 0.66) | -0.02 (-0.03, -0.00) | 0.62 (0.56, 0.67) | 0.03 (0.02, 0.04) | 0.56 (0.51, 0.62) | 0.07 (0.06, 0.09) | 0.56 (0.50, 0.62) | 0.10 (0.08, 0.11) | 0.61 (0.55, 0.68) | 0.10 (0.08, 0.12) | 0.70 (0.62, 0.77) |
| Portugal | -0.02 (-0.04, 0.00) | 1.16 (1.05, 1.26) | -0.04 (-0.07, -0.01) | 1.29 (1.19, 1.40) | 0.08 (0.05, 0.12) | 1.26 (1.14, 1.38) | 0.22 (0.17, 0.26) | 1.26 (1.12, 1.40) | 0.27 (0.23, 0.31) | 1.36 (1.21, 1.51) | 0.28 (0.23, 0.33) | 1.52 (1.36, 1.69) |
| Puerto Rico | -0.01 (-0.02, 0.00) | 0.63 (0.58, 0.69) | -0.02 (-0.03, -0.01) | 0.64 (0.58, 0.69) | 0.04 (0.02, 0.05) | 0.63 (0.57, 0.69) | 0.09 (0.08, 0.11) | 0.68 (0.61, 0.75) | 0.11 (0.09, 0.13) | 0.77 (0.69, 0.86) | 0.11 (0.09, 0.13) | 0.89 (0.80, 0.99) |
| Romania | -0.03 (-0.05, 0.00) | 1.50 (1.36, 1.64) | -0.06 (-0.09, -0.02) | 1.71 (1.57, 1.85) | 0.10 (0.06, 0.15) | 1.66 (1.50, 1.82) | 0.28 (0.22, 0.33) | 1.65 (1.47, 1.82) | 0.37 (0.31, 0.44) | 1.75 (1.56, 1.94) | 0.39 (0.33, 0.46) | 1.90 (1.70, 2.10) |
| South Africa | -0.02 (-0.05, 0.00) | 1.74 (1.58, 1.90) | -0.05 (-0.09, -0.02) | 1.88 (1.72, 2.03) | 0.10 (0.05, 0.14) | 1.74 (1.57, 1.91) | 0.25 (0.20, 0.30) | 1.66 (1.48, 1.84) | 0.32 (0.27, 0.37) | 1.74 (1.55, 1.92) | 0.33 (0.27, 0.38) | 1.94 (1.73, 2.15) |
| South Korea | -0.02 (-0.05, 0.00) | 1.13 (1.03, 1.24) | -0.05 (-0.09, -0.02) | 1.25 (1.15, 1.36) | 0.10 (0.06, 0.14) | 1.16 (1.04, 1.27) | 0.26 (0.21, 0.31) | 1.10 (0.98, 1.22) | 0.34 (0.29, 0.40) | 1.14 (1.02, 1.27) | 0.35 (0.29, 0.41) | 1.23 (1.10, 1.36) |
| Spain | -0.02 (-0.04, 0.00) | 1.07 (0.97, 1.17) | -0.04 (-0.07, -0.01) | 1.20 (1.10, 1.30) | 0.07 (0.04, 0.10) | 1.16 (1.05, 1.27) | 0.19 (0.15, 0.23) | 1.16 (1.04, 1.29) | 0.25 (0.21, 0.29) | 1.25 (1.11, 1.39) | 0.26 (0.21, 0.30) | 1.41 (1.26, 1.56) |
| Sweden | -0.02 (-0.05, 0.00) | 0.85 (0.77, 0.93) | -0.05 (-0.08, -0.02) | 0.97 (0.89, 1.05) | 0.09 (0.05, 0.13) | 0.95 (0.86, 1.04) | 0.24 (0.19, 0.29) | 0.96 (0.85, 1.06) | 0.31 (0.26, 0.36) | 1.02 (0.90, 1.13) | 0.32 (0.26, 0.37) | 1.12 (1.00, 1.24) |
| Switzerland | -0.02 (-0.05, 0.00) | 1.01 (0.92, 1.10) | -0.05 (-0.09, -0.02) | 1.14 (1.05, 1.24) | 0.10 (0.06, 0.14) | 1.08 (0.98, 1.19) | 0.26 (0.20, 0.31) | 1.06 (0.95, 1.18) | 0.34 (0.28, 0.40) | 1.12 (1.00, 1.25) | 0.35 (0.29, 0.41) | 1.24 (1.11, 1.38) |
| Taiwan | -0.02 (-0.04, 0.00) | 0.87 (0.79, 0.95) | -0.04 (-0.07, -0.01) | 0.95 (0.87, 1.03) | 0.08 (0.04, 0.11) | 0.89 (0.80, 0.97) | 0.20 (0.16, 0.24) | 0.90 (0.80, 1.00) | 0.27 (0.22, 0.31) | 0.95 (0.85, 1.06) | 0.28 (0.23, 0.33) | 1.09 (0.97, 1.20) |
| Thailand | -0.01 (-0.02, 0.00) | 1.07 (0.98, 1.17) | -0.02 (-0.04, -0.01) | 1.14 (1.04, 1.23) | 0.04 (0.02, 0.06) | 1.03 (0.93, 1.13) | 0.11 (0.09, 0.13) | 0.96 (0.85, 1.06) | 0.15 (0.12, 0.17) | 1.00 (0.89, 1.11) | 0.15 (0.13, 0.18) | 1.14 (1.02, 1.26) |
| UK | -0.02 (-0.04, 0.00) | 0.76 (0.69, 0.83) | -0.04 (-0.08, -0.01) | 0.86 (0.79, 0.94) | 0.08 (0.05, 0.12) | 0.84 (0.76, 0.92) | 0.21 (0.17, 0.25) | 0.83 (0.74, 0.92) | 0.27 (0.23, 0.32) | 0.89 (0.79, 0.99) | 0.28 (0.23, 0.33) | 1.00 (0.90, 1.11) |
| Uruguay | -0.03 (-0.06, 0.00) | 0.97 (0.88, 1.06) | -0.06 (-0.10, -0.02) | 1.12 (1.03, 1.22) | 0.10 (0.06, 0.15) | 1.05 (0.95, 1.15) | 0.27 (0.21, 0.32) | 0.99 (0.88, 1.10) | 0.32 (0.27, 0.38) | 1.01 (0.90, 1.12) | 0.34 (0.28, 0.40) | 1.14 (1.02, 1.26) |
| USA | -0.03 (-0.06, 0.00) | 1.07 (0.97, 1.17) | -0.06 (-0.11, -0.02) | 1.19 (1.09, 1.28) | 0.12 (0.07, 0.17) | 1.12 (1.01, 1.22) | 0.31 (0.25, 0.37) | 1.08 (0.96, 1.20) | 0.41 (0.34, 0.48) | 1.12 (1.00, 1.25) | 0.42 (0.35, 0.49) | 1.21 (1.08, 1.34) |
| Vietnam | -0.01 (-0.02, 0.00) | 0.72 (0.66, 0.79) | -0.02 (-0.04, -0.01) | 0.68 (0.62, 0.74) | 0.04 (0.02, 0.06) | 0.65 (0.58, 0.71) | 0.10 (0.08, 0.12) | 0.72 (0.65, 0.80) | 0.12 (0.10, 0.15) | 0.84 (0.75, 0.93) | 0.13 (0.10, 0.15) | 0.99 (0.88, 1.09) |
| Pooled | -0.02 (-0.05, 0.00) | 1.09 (0.99, 1.19) | -0.05 (-0.09, -0.02) | 1.20 (1.10, 1.30) | 0.10 (0.05, 0.14) | 1.13 (1.02, 1.24) | 0.25 (0.20, 0.30) | 1.10 (0.98, 1.22) | 0.33 (0.27, 0.38) | 1.16 (1.03, 1.29) | 0.33 (0.28, 0.39) | 1.28 (1.14, 1.41) |

## Table S18. Attributable fractions (%) of both inter-day TV and intra-day TV for cardiovascular mortality

| Country/region | TV 0–1 | | TV 0–2 | | TV 0–3 | | TV 0–4 | | TV 0–5 | | TV 0–6 | |
| --- | --- | --- | --- | --- | --- | --- | --- | --- | --- | --- | --- | --- |
|  | Inter-day | Intra-day | Inter-day | Intra-day | Inter-day | Intra-day | Inter-day | Intra-day | Inter-day | Intra-day | Inter-day | Intra-day |
| Brazil | 0.06 (0.03, 0.09) | 1.05 (0.91, 1.19) | 0.09 (0.04, 0.13) | 1.31 (1.15, 1.46) | 0.21 (0.16, 0.26) | 1.39 (1.22, 1.57) | 0.33 (0.27, 0.40) | 1.43 (1.24, 1.63) | 0.44 (0.37, 0.50) | 1.48 (1.27, 1.69) | 0.46 (0.38, 0.53) | 1.64 (1.41, 1.88) |
| Canada | 0.12 (0.06, 0.17) | 1.12 (0.97, 1.27) | 0.17 (0.08, 0.26) | 1.40 (1.23, 1.57) | 0.42 (0.31, 0.53) | 1.51 (1.32, 1.70) | 0.66 (0.54, 0.79) | 1.54 (1.33, 1.75) | 0.87 (0.74, 1.00) | 1.58 (1.36, 1.81) | 0.90 (0.76, 1.04) | 1.72 (1.47, 1.96) |
| China | 0.07 (0.04, 0.11) | 0.88 (0.77, 1.00) | 0.11 (0.05, 0.16) | 1.10 (0.97, 1.24) | 0.26 (0.19, 0.32) | 1.15 (1.00, 1.29) | 0.40 (0.33, 0.48) | 1.11 (0.96, 1.27) | 0.53 (0.45, 0.62) | 1.13 (0.97, 1.29) | 0.56 (0.47, 0.65) | 1.22 (1.05, 1.40) |
| Colombia | 0.04 (0.02, 0.06) | 0.86 (0.75, 0.98) | 0.06 (0.03, 0.09) | 0.96 (0.84, 1.07) | 0.13 (0.10, 0.17) | 1.01 (0.88, 1.14) | 0.20 (0.16, 0.24) | 1.03 (0.89, 1.17) | 0.24 (0.21, 0.28) | 1.09 (0.93, 1.24) | 0.25 (0.21, 0.29) | 1.22 (1.04, 1.39) |
| Costa Rica | 0.04 (0.02, 0.05) | 0.95 (0.82, 1.08) | 0.05 (0.02, 0.08) | 1.10 (0.97, 1.23) | 0.12 (0.09, 0.15) | 1.10 (0.96, 1.24) | 0.19 (0.15, 0.22) | 1.15 (0.99, 1.31) | 0.24 (0.20, 0.27) | 1.25 (1.07, 1.43) | 0.21 (0.18, 0.24) | 1.41 (1.21, 1.61) |
| Czech Republic | 0.09 (0.05, 0.14) | 0.80 (0.69, 0.90) | 0.14 (0.07, 0.21) | 1.04 (0.91, 1.16) | 0.35 (0.26, 0.44) | 1.12 (0.98, 1.26) | 0.55 (0.45, 0.65) | 1.14 (0.98, 1.30) | 0.72 (0.61, 0.83) | 1.17 (1.00, 1.34) | 0.76 (0.64, 0.88) | 1.28 (1.09, 1.46) |
| Ecuador | 0.04 (0.02, 0.06) | 0.95 (0.82, 1.07) | 0.06 (0.03, 0.09) | 0.92 (0.81, 1.03) | 0.14 (0.10, 0.17) | 0.83 (0.72, 0.93) | 0.22 (0.18, 0.26) | 0.87 (0.75, 0.98) | 0.26 (0.22, 0.30) | 0.96 (0.82, 1.10) | 0.23 (0.19, 0.27) | 1.12 (0.96, 1.28) |
| Estonia | 0.09 (0.04, 0.13) | 0.97 (0.84, 1.10) | 0.13 (0.06, 0.20) | 1.24 (1.09, 1.39) | 0.32 (0.24, 0.40) | 1.33 (1.16, 1.50) | 0.50 (0.40, 0.59) | 1.35 (1.17, 1.54) | 0.64 (0.54, 0.74) | 1.35 (1.16, 1.55) | 0.68 (0.57, 0.79) | 1.45 (1.24, 1.65) |
| Finland | 0.08 (0.04, 0.12) | 0.83 (0.72, 0.94) | 0.12 (0.06, 0.18) | 1.05 (0.92, 1.18) | 0.30 (0.22, 0.38) | 1.12 (0.98, 1.27) | 0.49 (0.39, 0.58) | 1.16 (1.00, 1.32) | 0.65 (0.55, 0.75) | 1.20 (1.03, 1.37) | 0.67 (0.57, 0.78) | 1.33 (1.14, 1.52) |
| Greece | 0.06 (0.03, 0.09) | 1.00 (0.87, 1.13) | 0.09 (0.04, 0.14) | 1.22 (1.07, 1.36) | 0.22 (0.17, 0.28) | 1.32 (1.15, 1.49) | 0.36 (0.29, 0.43) | 1.34 (1.16, 1.53) | 0.49 (0.42, 0.56) | 1.40 (1.20, 1.60) | 0.53 (0.44, 0.61) | 1.55 (1.33, 1.77) |
| Iran | 0.08 (0.04, 0.12) | 1.24 (1.07, 1.40) | 0.12 (0.06, 0.18) | 1.52 (1.34, 1.71) | 0.28 (0.21, 0.36) | 1.56 (1.36, 1.76) | 0.45 (0.37, 0.53) | 1.66 (1.43, 1.88) | 0.60 (0.51, 0.70) | 1.76 (1.51, 2.01) | 0.64 (0.54, 0.74) | 1.95 (1.67, 2.22) |
| Ireland | 0.06 (0.03, 0.09) | 0.81 (0.70, 0.92) | 0.09 (0.04, 0.14) | 1.02 (0.90, 1.15) | 0.22 (0.16, 0.28) | 1.10 (0.96, 1.24) | 0.35 (0.29, 0.42) | 1.13 (0.98, 1.29) | 0.46 (0.39, 0.53) | 1.10 (0.94, 1.25) | 0.47 (0.40, 0.55) | 1.18 (1.01, 1.35) |
| Japan | 0.07 (0.04, 0.11) | 0.99 (0.86, 1.12) | 0.11 (0.05, 0.17) | 1.21 (1.06, 1.35) | 0.26 (0.19, 0.33) | 1.27 (1.11, 1.44) | 0.41 (0.33, 0.48) | 1.29 (1.11, 1.47) | 0.53 (0.45, 0.61) | 1.31 (1.12, 1.50) | 0.55 (0.46, 0.64) | 1.40 (1.20, 1.60) |
| Kuwait | 0.05 (0.02, 0.07) | 0.87 (0.75, 0.99) | 0.07 (0.04, 0.11) | 1.07 (0.94, 1.20) | 0.18 (0.13, 0.22) | 1.14 (1.00, 1.29) | 0.29 (0.23, 0.34) | 1.14 (0.98, 1.29) | 0.39 (0.33, 0.45) | 1.10 (0.94, 1.26) | 0.42 (0.35, 0.49) | 1.17 (1.00, 1.33) |
| Mexico | 0.05 (0.02, 0.07) | 1.45 (1.26, 1.64) | 0.07 (0.04, 0.11) | 1.76 (1.54, 1.97) | 0.18 (0.13, 0.23) | 1.77 (1.55, 2.00) | 0.28 (0.23, 0.34) | 1.70 (1.47, 1.93) | 0.37 (0.32, 0.43) | 1.63 (1.40, 1.86) | 0.39 (0.33, 0.46) | 1.80 (1.55, 2.06) |
| Norway | 0.08 (0.04, 0.13) | 0.93 (0.81, 1.05) | 0.13 (0.06, 0.19) | 1.19 (1.05, 1.33) | 0.31 (0.23, 0.39) | 1.30 (1.14, 1.47) | 0.50 (0.40, 0.59) | 1.35 (1.16, 1.53) | 0.63 (0.54, 0.73) | 1.38 (1.18, 1.57) | 0.69 (0.58, 0.80) | 1.47 (1.26, 1.68) |
| Panama | 0.04 (0.02, 0.06) | 0.79 (0.68, 0.89) | 0.05 (0.02, 0.08) | 0.85 (0.74, 0.95) | 0.12 (0.09, 0.15) | 0.93 (0.81, 1.05) | 0.19 (0.16, 0.23) | 0.90 (0.78, 1.02) | 0.25 (0.21, 0.29) | 0.87 (0.74, 0.99) | 0.26 (0.22, 0.31) | 0.93 (0.80, 1.07) |
| Paraguay | 0.10 (0.05, 0.15) | 1.33 (1.15, 1.50) | 0.15 (0.07, 0.23) | 1.60 (1.41, 1.79) | 0.38 (0.28, 0.48) | 1.73 (1.51, 1.95) | 0.63 (0.51, 0.75) | 1.75 (1.51, 1.99) | 0.85 (0.72, 0.98) | 1.78 (1.53, 2.04) | 0.92 (0.78, 1.06) | 1.92 (1.64, 2.19) |
| Philippines | 0.02 (0.01, 0.04) | 0.58 (0.51, 0.66) | 0.04 (0.02, 0.05) | 0.67 (0.59, 0.75) | 0.08 (0.06, 0.11) | 0.69 (0.60, 0.77) | 0.13 (0.11, 0.16) | 0.70 (0.61, 0.80) | 0.17 (0.14, 0.19) | 0.74 (0.63, 0.85) | 0.18 (0.15, 0.21) | 0.82 (0.71, 0.94) |
| Portugal | 0.06 (0.03, 0.10) | 1.11 (0.96, 1.26) | 0.10 (0.05, 0.15) | 1.39 (1.22, 1.56) | 0.24 (0.18, 0.30) | 1.50 (1.31, 1.69) | 0.38 (0.31, 0.45) | 1.56 (1.34, 1.77) | 0.47 (0.40, 0.54) | 1.62 (1.39, 1.85) | 0.50 (0.42, 0.58) | 1.78 (1.52, 2.03) |
| South Africa | 0.08 (0.04, 0.12) | 1.69 (1.47, 1.92) | 0.11 (0.06, 0.17) | 2.05 (1.80, 2.29) | 0.27 (0.20, 0.34) | 2.11 (1.85, 2.38) | 0.43 (0.35, 0.51) | 2.09 (1.80, 2.37) | 0.56 (0.47, 0.64) | 2.11 (1.81, 2.40) | 0.58 (0.49, 0.67) | 2.30 (1.97, 2.62) |
| South Korea | 0.08 (0.04, 0.12) | 1.09 (0.95, 1.24) | 0.12 (0.06, 0.18) | 1.35 (1.19, 1.51) | 0.29 (0.21, 0.36) | 1.39 (1.21, 1.57) | 0.46 (0.38, 0.55) | 1.37 (1.18, 1.56) | 0.61 (0.52, 0.70) | 1.37 (1.18, 1.57) | 0.64 (0.54, 0.74) | 1.44 (1.24, 1.65) |
| Spain | 0.06 (0.03, 0.08) | 1.03 (0.89, 1.16) | 0.09 (0.04, 0.13) | 1.29 (1.13, 1.44) | 0.21 (0.15, 0.26) | 1.39 (1.21, 1.56) | 0.33 (0.27, 0.39) | 1.43 (1.24, 1.63) | 0.44 (0.37, 0.50) | 1.49 (1.27, 1.70) | 0.46 (0.39, 0.53) | 1.64 (1.41, 1.87) |
| Sweden | 0.07 (0.04, 0.11) | 0.82 (0.71, 0.93) | 0.11 (0.05, 0.16) | 1.04 (0.92, 1.17) | 0.26 (0.19, 0.33) | 1.14 (1.00, 1.29) | 0.42 (0.34, 0.50) | 1.19 (1.02, 1.35) | 0.54 (0.46, 0.63) | 1.22 (1.04, 1.39) | 0.57 (0.48, 0.66) | 1.31 (1.13, 1.50) |
| Switzerland | 0.08 (0.04, 0.11) | 0.97 (0.84, 1.09) | 0.12 (0.06, 0.18) | 1.23 (1.08, 1.37) | 0.28 (0.21, 0.35) | 1.30 (1.13, 1.46) | 0.45 (0.37, 0.54) | 1.31 (1.13, 1.49) | 0.60 (0.51, 0.69) | 1.34 (1.15, 1.53) | 0.63 (0.53, 0.73) | 1.45 (1.24, 1.65) |
| Taiwan | 0.06 (0.03, 0.09) | 0.84 (0.73, 0.95) | 0.10 (0.05, 0.15) | 1.04 (0.91, 1.16) | 0.23 (0.17, 0.29) | 1.08 (0.94, 1.22) | 0.37 (0.30, 0.44) | 1.13 (0.97, 1.28) | 0.48 (0.41, 0.55) | 1.15 (0.99, 1.32) | 0.51 (0.43, 0.59) | 1.29 (1.10, 1.47) |
| Thailand | 0.03 (0.02, 0.05) | 1.03 (0.89, 1.16) | 0.05 (0.02, 0.08) | 1.22 (1.08, 1.37) | 0.12 (0.09, 0.15) | 1.23 (1.08, 1.39) | 0.19 (0.16, 0.23) | 1.19 (1.03, 1.35) | 0.25 (0.22, 0.29) | 1.20 (1.03, 1.37) | 0.27 (0.23, 0.31) | 1.33 (1.14, 1.52) |
| UK | 0.07 (0.03, 0.10) | 0.73 (0.63, 0.82) | 0.10 (0.05, 0.15) | 0.93 (0.82, 1.04) | 0.24 (0.17, 0.30) | 1.01 (0.88, 1.14) | 0.37 (0.30, 0.44) | 1.03 (0.89, 1.17) | 0.48 (0.41, 0.55) | 1.06 (0.91, 1.21) | 0.50 (0.42, 0.58) | 1.17 (1.00, 1.34) |
| USA | 0.09 (0.05, 0.14) | 1.02 (0.89, 1.16) | 0.14 (0.07, 0.22) | 1.27 (1.12, 1.42) | 0.35 (0.26, 0.43) | 1.33 (1.16, 1.50) | 0.55 (0.45, 0.65) | 1.33 (1.15, 1.52) | 0.72 (0.61, 0.83) | 1.34 (1.15, 1.53) | 0.75 (0.64, 0.87) | 1.41 (1.21, 1.61) |
| Vietnam | 0.03 (0.02, 0.05) | 0.71 (0.61, 0.80) | 0.05 (0.02, 0.07) | 0.76 (0.67, 0.85) | 0.11 (0.08, 0.14) | 0.81 (0.70, 0.91) | 0.18 (0.14, 0.21) | 0.93 (0.80, 1.06) | 0.23 (0.19, 0.26) | 1.03 (0.88, 1.18) | 0.23 (0.20, 0.27) | 1.18 (1.02, 1.35) |
| Pooled | 0.08 (0.04, 0.12) | 1.02 (0.88, 1.15) | 0.12 (0.06, 0.18) | 1.26 (1.10, 1.41) | 0.28 (0.21, 0.36) | 1.32 (1.16, 1.49) | 0.45 (0.36, 0.53) | 1.34 (1.15, 1.52) | 0.58 (0.50, 0.67) | 1.35 (1.16, 1.55) | 0.61 (0.51, 0.70) | 1.45 (1.25, 1.66) |

## Table S19. Attributable fractions (%) of both inter-day TV and intra-day TV for respiratory mortality

| Country/region | TV 0–1 | | TV 0–2 | | TV 0–3 | | TV 0–4 | | TV 0–5 | | TV 0–6 | |
| --- | --- | --- | --- | --- | --- | --- | --- | --- | --- | --- | --- | --- |
|  | Inter-day | Intra-day | Inter-day | Intra-day | Inter-day | Intra-day | Inter-day | Intra-day | Inter-day | Intra-day | Inter-day | Intra-day |
| Brazil | 0.02 (-0.03, 0.08) | 0.52 (0.30, 0.73) | -0.00 (-0.08, 0.07) | 0.72 (0.48, 0.96) | 0.16 (0.07, 0.25) | 0.74 (0.46, 1.01) | 0.31 (0.21, 0.41) | 0.91 (0.58, 1.23) | 0.34 (0.22, 0.46) | 1.14 (0.76, 1.51) | 0.33 (0.21, 0.45) | 1.38 (0.97, 1.78) |
| Canada | 0.05 (-0.07, 0.17) | 0.54 (0.31, 0.77) | -0.01 (-0.16, 0.14) | 0.76 (0.51, 1.01) | 0.33 (0.15, 0.51) | 0.78 (0.49, 1.07) | 0.63 (0.42, 0.83) | 0.96 (0.62, 1.30) | 0.69 (0.45, 0.92) | 1.19 (0.80, 1.58) | 0.66 (0.41, 0.90) | 1.42 (1.00, 1.83) |
| China | 0.03 (-0.04, 0.09) | 0.38 (0.22, 0.54) | -0.00 (-0.09, 0.08) | 0.54 (0.36, 0.71) | 0.18 (0.08, 0.29) | 0.54 (0.34, 0.74) | 0.35 (0.23, 0.47) | 0.63 (0.40, 0.85) | 0.39 (0.25, 0.52) | 0.77 (0.51, 1.02) | 0.38 (0.24, 0.52) | 0.90 (0.64, 1.17) |
| Colombia | 0.02 (-0.02, 0.06) | 0.43 (0.25, 0.61) | -0.00 (-0.05, 0.05) | 0.52 (0.35, 0.70) | 0.10 (0.05, 0.16) | 0.53 (0.33, 0.73) | 0.19 (0.13, 0.25) | 0.65 (0.42, 0.89) | 0.19 (0.13, 0.26) | 0.83 (0.56, 1.11) | 0.18 (0.11, 0.25) | 1.02 (0.72, 1.32) |
| Costa Rica | 0.02 (-0.02, 0.05) | 0.46 (0.27, 0.66) | -0.00 (-0.05, 0.04) | 0.60 (0.40, 0.79) | 0.09 (0.04, 0.15) | 0.57 (0.36, 0.78) | 0.18 (0.12, 0.24) | 0.72 (0.46, 0.98) | 0.19 (0.12, 0.25) | 0.94 (0.63, 1.25) | 0.15 (0.10, 0.21) | 1.16 (0.82, 1.51) |
| Czech Republic | 0.04 (-0.06, 0.14) | 0.38 (0.22, 0.55) | -0.01 (-0.13, 0.11) | 0.56 (0.38, 0.75) | 0.27 (0.12, 0.42) | 0.58 (0.37, 0.80) | 0.52 (0.35, 0.69) | 0.71 (0.46, 0.97) | 0.57 (0.37, 0.76) | 0.89 (0.59, 1.18) | 0.56 (0.35, 0.76) | 1.06 (0.75, 1.37) |
| Ecuador | 0.02 (-0.03, 0.06) | 0.48 (0.28, 0.69) | -0.00 (-0.06, 0.05) | 0.53 (0.36, 0.71) | 0.11 (0.05, 0.17) | 0.47 (0.30, 0.65) | 0.21 (0.14, 0.28) | 0.58 (0.38, 0.79) | 0.20 (0.13, 0.27) | 0.78 (0.52, 1.04) | 0.17 (0.11, 0.23) | 0.99 (0.70, 1.28) |
| Estonia | 0.04 (-0.05, 0.13) | 0.48 (0.28, 0.68) | -0.01 (-0.12, 0.11) | 0.68 (0.46, 0.91) | 0.25 (0.11, 0.39) | 0.70 (0.44, 0.96) | 0.47 (0.32, 0.63) | 0.86 (0.55, 1.16) | 0.51 (0.33, 0.69) | 1.04 (0.69, 1.38) | 0.50 (0.32, 0.69) | 1.22 (0.86, 1.57) |
| Finland | 0.04 (-0.05, 0.12) | 0.41 (0.24, 0.58) | -0.01 (-0.11, 0.10) | 0.58 (0.39, 0.77) | 0.25 (0.11, 0.38) | 0.60 (0.38, 0.81) | 0.48 (0.32, 0.64) | 0.74 (0.48, 1.00) | 0.53 (0.35, 0.72) | 0.93 (0.62, 1.23) | 0.51 (0.32, 0.69) | 1.12 (0.79, 1.45) |
| France | 0.03 (-0.04, 0.11) | 0.49 (0.28, 0.69) | -0.00 (-0.10, 0.09) | 0.69 (0.46, 0.92) | 0.21 (0.09, 0.33) | 0.70 (0.44, 0.95) | 0.41 (0.27, 0.55) | 0.86 (0.55, 1.17) | 0.44 (0.29, 0.59) | 1.07 (0.72, 1.42) | 0.42 (0.27, 0.58) | 1.29 (0.91, 1.66) |
| Greece | 0.03 (-0.04, 0.09) | 0.49 (0.29, 0.70) | -0.00 (-0.08, 0.07) | 0.67 (0.45, 0.90) | 0.18 (0.08, 0.27) | 0.70 (0.44, 0.96) | 0.34 (0.23, 0.45) | 0.85 (0.55, 1.16) | 0.38 (0.25, 0.52) | 1.08 (0.72, 1.43) | 0.38 (0.24, 0.52) | 1.30 (0.92, 1.69) |
| Iran | 0.03 (-0.05, 0.12) | 0.60 (0.35, 0.85) | -0.01 (-0.11, 0.10) | 0.83 (0.55, 1.10) | 0.23 (0.10, 0.35) | 0.81 (0.51, 1.11) | 0.43 (0.29, 0.58) | 1.04 (0.67, 1.41) | 0.48 (0.32, 0.65) | 1.33 (0.89, 1.76) | 0.48 (0.30, 0.65) | 1.62 (1.14, 2.09) |
| Ireland | 0.03 (-0.04, 0.09) | 0.39 (0.23, 0.56) | -0.00 (-0.08, 0.08) | 0.56 (0.37, 0.74) | 0.18 (0.08, 0.27) | 0.57 (0.36, 0.78) | 0.34 (0.23, 0.45) | 0.71 (0.46, 0.96) | 0.37 (0.24, 0.49) | 0.83 (0.56, 1.11) | 0.35 (0.22, 0.48) | 0.99 (0.70, 1.28) |
| Japan | 0.03 (-0.04, 0.11) | 0.48 (0.28, 0.68) | -0.00 (-0.10, 0.09) | 0.66 (0.44, 0.88) | 0.20 (0.09, 0.31) | 0.67 (0.42, 0.91) | 0.38 (0.25, 0.51) | 0.81 (0.52, 1.10) | 0.41 (0.27, 0.56) | 1.00 (0.67, 1.32) | 0.39 (0.25, 0.54) | 1.16 (0.82, 1.51) |
| Kuwait | 0.02 (-0.03, 0.07) | 0.42 (0.24, 0.60) | -0.00 (-0.07, 0.06) | 0.58 (0.39, 0.77) | 0.14 (0.06, 0.22) | 0.59 (0.37, 0.81) | 0.27 (0.18, 0.36) | 0.71 (0.45, 0.96) | 0.31 (0.20, 0.41) | 0.82 (0.55, 1.10) | 0.31 (0.19, 0.42) | 0.96 (0.68, 1.24) |
| Mexico | 0.02 (-0.03, 0.07) | 0.73 (0.42, 1.04) | -0.00 (-0.07, 0.06) | 0.99 (0.66, 1.32) | 0.14 (0.06, 0.22) | 0.96 (0.61, 1.31) | 0.26 (0.18, 0.35) | 1.11 (0.71, 1.50) | 0.29 (0.19, 0.39) | 1.29 (0.86, 1.71) | 0.28 (0.18, 0.38) | 1.56 (1.10, 2.01) |
| Norway | 0.04 (-0.05, 0.12) | 0.45 (0.26, 0.65) | -0.01 (-0.12, 0.10) | 0.65 (0.43, 0.86) | 0.25 (0.11, 0.39) | 0.68 (0.43, 0.93) | 0.48 (0.32, 0.64) | 0.85 (0.54, 1.15) | 0.52 (0.34, 0.69) | 1.05 (0.70, 1.39) | 0.52 (0.33, 0.71) | 1.23 (0.86, 1.59) |
| Panama | 0.02 (-0.02, 0.05) | 0.38 (0.22, 0.54) | -0.00 (-0.05, 0.04) | 0.45 (0.30, 0.60) | 0.09 (0.04, 0.15) | 0.48 (0.30, 0.65) | 0.18 (0.12, 0.24) | 0.56 (0.36, 0.75) | 0.19 (0.13, 0.26) | 0.64 (0.43, 0.86) | 0.19 (0.12, 0.26) | 0.76 (0.54, 0.98) |
| Paraguay | 0.04 (-0.06, 0.14) | 0.65 (0.37, 0.92) | -0.01 (-0.14, 0.12) | 0.88 (0.58, 1.17) | 0.30 (0.13, 0.47) | 0.91 (0.58, 1.25) | 0.61 (0.41, 0.81) | 1.11 (0.71, 1.50) | 0.68 (0.44, 0.91) | 1.36 (0.92, 1.81) | 0.67 (0.43, 0.92) | 1.61 (1.13, 2.08) |
| Philippines | 0.01 (-0.01, 0.03) | 0.29 (0.16, 0.41) | -0.00 (-0.03, 0.03) | 0.37 (0.24, 0.49) | 0.07 (0.03, 0.10) | 0.36 (0.23, 0.49) | 0.12 (0.08, 0.16) | 0.44 (0.29, 0.60) | 0.13 (0.09, 0.18) | 0.56 (0.38, 0.75) | 0.13 (0.08, 0.18) | 0.69 (0.48, 0.89) |
| Portugal | 0.03 (-0.04, 0.09) | 0.54 (0.31, 0.77) | -0.00 (-0.09, 0.08) | 0.76 (0.50, 1.01) | 0.19 (0.08, 0.29) | 0.79 (0.50, 1.08) | 0.36 (0.24, 0.48) | 0.98 (0.63, 1.33) | 0.37 (0.24, 0.50) | 1.23 (0.83, 1.64) | 0.37 (0.23, 0.50) | 1.48 (1.05, 1.92) |
| South Africa | 0.03 (-0.05, 0.11) | 0.86 (0.50, 1.22) | -0.00 (-0.10, 0.09) | 1.16 (0.78, 1.55) | 0.22 (0.09, 0.34) | 1.15 (0.73, 1.57) | 0.41 (0.27, 0.54) | 1.37 (0.88, 1.85) | 0.44 (0.29, 0.59) | 1.66 (1.11, 2.20) | 0.42 (0.27, 0.58) | 1.99 (1.40, 2.57) |
| South Korea | 0.03 (-0.05, 0.12) | 0.55 (0.32, 0.78) | -0.01 (-0.11, 0.10) | 0.76 (0.51, 1.01) | 0.23 (0.10, 0.36) | 0.75 (0.47, 1.03) | 0.44 (0.30, 0.59) | 0.89 (0.57, 1.20) | 0.48 (0.32, 0.65) | 1.07 (0.72, 1.42) | 0.47 (0.30, 0.64) | 1.23 (0.87, 1.60) |
| Spain | 0.02 (-0.03, 0.08) | 0.51 (0.30, 0.73) | -0.00 (-0.08, 0.07) | 0.72 (0.48, 0.97) | 0.17 (0.07, 0.26) | 0.75 (0.47, 1.03) | 0.32 (0.21, 0.43) | 0.93 (0.60, 1.27) | 0.35 (0.23, 0.47) | 1.17 (0.79, 1.55) | 0.34 (0.21, 0.46) | 1.42 (1.00, 1.83) |
| Sweden | 0.03 (-0.04, 0.11) | 0.40 (0.23, 0.57) | -0.00 (-0.10, 0.09) | 0.57 (0.38, 0.76) | 0.21 (0.09, 0.32) | 0.60 (0.38, 0.82) | 0.41 (0.27, 0.54) | 0.75 (0.48, 1.01) | 0.43 (0.28, 0.58) | 0.92 (0.62, 1.22) | 0.42 (0.26, 0.57) | 1.09 (0.77, 1.41) |
| Switzerland | 0.03 (-0.04, 0.11) | 0.47 (0.27, 0.67) | -0.00 (-0.10, 0.09) | 0.67 (0.45, 0.89) | 0.22 (0.10, 0.34) | 0.68 (0.43, 0.92) | 0.42 (0.28, 0.56) | 0.82 (0.53, 1.11) | 0.47 (0.31, 0.63) | 1.01 (0.68, 1.34) | 0.45 (0.28, 0.62) | 1.20 (0.85, 1.55) |
| Taiwan | 0.03 (-0.04, 0.09) | 0.42 (0.24, 0.59) | -0.00 (-0.08, 0.08) | 0.57 (0.38, 0.76) | 0.18 (0.08, 0.28) | 0.57 (0.36, 0.78) | 0.35 (0.23, 0.46) | 0.72 (0.46, 0.98) | 0.37 (0.24, 0.50) | 0.89 (0.60, 1.19) | 0.37 (0.23, 0.50) | 1.09 (0.77, 1.41) |
| Thailand | 0.01 (-0.02, 0.05) | 0.51 (0.30, 0.73) | -0.00 (-0.05, 0.04) | 0.69 (0.46, 0.91) | 0.09 (0.04, 0.15) | 0.66 (0.42, 0.91) | 0.18 (0.12, 0.24) | 0.77 (0.49, 1.04) | 0.20 (0.13, 0.27) | 0.94 (0.63, 1.25) | 0.20 (0.12, 0.27) | 1.13 (0.80, 1.47) |
| UK | 0.03 (-0.04, 0.10) | 0.35 (0.20, 0.50) | -0.00 (-0.09, 0.08) | 0.50 (0.33, 0.67) | 0.19 (0.08, 0.29) | 0.52 (0.33, 0.71) | 0.36 (0.24, 0.48) | 0.64 (0.41, 0.87) | 0.39 (0.25, 0.52) | 0.80 (0.53, 1.06) | 0.37 (0.23, 0.51) | 0.96 (0.68, 1.24) |
| USA | 0.04 (-0.06, 0.14) | 0.51 (0.30, 0.73) | -0.01 (-0.13, 0.12) | 0.71 (0.48, 0.95) | 0.27 (0.12, 0.42) | 0.72 (0.45, 0.98) | 0.52 (0.35, 0.69) | 0.87 (0.56, 1.17) | 0.57 (0.37, 0.77) | 1.05 (0.70, 1.39) | 0.55 (0.35, 0.75) | 1.21 (0.85, 1.57) |
| Vietnam | 0.01 (-0.02, 0.05) | 0.34 (0.19, 0.48) | -0.00 (-0.04, 0.04) | 0.39 (0.26, 0.52) | 0.08 (0.04, 0.13) | 0.40 (0.25, 0.54) | 0.16 (0.10, 0.21) | 0.56 (0.36, 0.76) | 0.17 (0.11, 0.22) | 0.75 (0.51, 1.00) | 0.16 (0.10, 0.22) | 0.95 (0.67, 1.23) |
| Pooled | 0.03 (-0.04, 0.11) | 0.52 (0.30, 0.73) | -0.00 (-0.10, 0.09) | 0.71 (0.47, 0.95) | 0.21 (0.09, 0.33) | 0.72 (0.45, 0.98) | 0.41 (0.27, 0.54) | 0.87 (0.56, 1.18) | 0.44 (0.29, 0.59) | 1.06 (0.71, 1.41) | 0.42 (0.27, 0.58) | 1.25 (0.88, 1.62) |

## Table S20. Annual average attributable deaths of both inter-day TV and intra-day TV for all-cause mortality

| Country/region | TV 0–1 | | TV 0–2 | | TV 0–3 | | TV 0–4 | | TV 0–5 | | TV 0–6 | |
| --- | --- | --- | --- | --- | --- | --- | --- | --- | --- | --- | --- | --- |
|  | Inter-day | Intra-day | Inter-day | Intra-day | Inter-day | Intra-day | Inter-day | Intra-day | Inter-day | Intra-day | Inter-day | Intra-day |
| Argentina | -19 (-40, 2) | 781 (710, 853) | -40 (-68, -13) | 900 (826, 974) | 76 (43, 108) | 847 (765, 928) | 195 (157, 233) | 818 (729, 906) | 255 (213, 297) | 832 (741, 923) | 258 (214, 301) | 883 (789, 976) |
| Australia | -13 (-28, 2) | 506 (460, 553) | -29 (-48, -9) | 547 (502, 592) | 52 (30, 75) | 521 (471, 572) | 133 (107, 159) | 513 (457, 568) | 172 (144, 201) | 572 (509, 634) | 177 (147, 207) | 656 (586, 725) |
| Brazil | -31 (-66, 4) | 1,925 (1,749, 2,100) | -68 (-115, -22) | 2,134 (1,958, 2,310) | 127 (72, 183) | 2,039 (1,843, 2,235) | 328 (263, 392) | 2,035 (1,814, 2,255) | 431 (360, 502) | 2,183 (1,943, 2,422) | 441 (366, 516) | 2,474 (2,211, 2,736) |
| Canada | -44 (-94, 6) | 1,443 (1,311, 1,575) | -97 (-164, -31) | 1,608 (1,475, 1,740) | 181 (102, 259) | 1,553 (1,403, 1,702) | 465 (374, 557) | 1,533 (1,366, 1,699) | 611 (511, 711) | 1,636 (1,456, 1,815) | 622 (517, 728) | 1,821 (1,628, 2,014) |
| Chile | -7 (-16, 1) | 605 (550, 660) | -16 (-27, -5) | 668 (613, 723) | 29 (16, 41) | 616 (557, 675) | 72 (58, 87) | 601 (536, 666) | 96 (80, 111) | 610 (543, 677) | 99 (83, 116) | 665 (594, 735) |
| China | -51 (-108, 6) | 2,229 (2,025, 2,432) | -112 (-189, -35) | 2,483 (2,278, 2,688) | 208 (118, 298) | 2,317 (2,094, 2,540) | 534 (429, 639) | 2,186 (1,949, 2,423) | 707 (591, 823) | 2,298 (2,046, 2,550) | 731 (607, 855) | 2,559 (2,287, 2,831) |
| Colombia | -8 (-16, 1) | 536 (487, 585) | -16 (-27, -5) | 527 (484, 571) | 27 (16, 39) | 499 (451, 547) | 68 (55, 81) | 494 (440, 547) | 83 (70, 97) | 542 (482, 601) | 84 (69, 98) | 620 (554, 686) |
| Costa Rica | -0 (-0, 0) | 17 (15, 19) | -0 (-1, -0) | 18 (16, 19) | 1 (0, 1) | 16 (14, 17) | 2 (1, 2) | 16 (14, 18) | 2 (2, 3) | 18 (16, 20) | 2 (2, 2) | 21 (18, 23) |
| Czech Republic | -9 (-20, 1) | 268 (243, 293) | -21 (-35, -6) | 312 (286, 337) | 39 (22, 56) | 302 (273, 331) | 101 (81, 121) | 298 (265, 330) | 133 (111, 154) | 317 (282, 352) | 138 (115, 162) | 353 (316, 391) |
| Ecuador | -3 (-6, 0) | 227 (207, 248) | -6 (-10, -2) | 199 (183, 215) | 11 (6, 16) | 164 (148, 180) | 28 (23, 34) | 164 (146, 182) | 33 (28, 38) | 188 (167, 209) | 29 (24, 34) | 223 (200, 247) |
| Estonia | -2 (-4, 0) | 77 (70, 84) | -4 (-8, -1) | 88 (81, 95) | 8 (5, 12) | 84 (76, 93) | 21 (17, 26) | 83 (74, 92) | 28 (23, 32) | 86 (77, 96) | 29 (24, 34) | 94 (84, 104) |
| Finland | -2 (-4, 0) | 63 (57, 68) | -4 (-7, -1) | 71 (65, 77) | 8 (4, 11) | 68 (61, 74) | 20 (16, 24) | 68 (61, 75) | 27 (23, 31) | 73 (65, 81) | 27 (23, 32) | 82 (74, 91) |
| France | -26 (-54, 3) | 1,154 (1,049, 1,260) | -56 (-94, -18) | 1,307 (1,199, 1,414) | 104 (59, 149) | 1,234 (1,115, 1,353) | 270 (216, 322) | 1,229 (1,096, 1,363) | 346 (289, 402) | 1,308 (1,165, 1,452) | 355 (295, 415) | 1,470 (1,314, 1,626) |
| French Caribbean | -0 (-1, 0) | 20 (18, 22) | -1 (-1, -0) | 23 (21, 25) | 1 (1, 1) | 21 (19, 23) | 2 (2, 3) | 22 (19, 24) | 3 (2, 3) | 25 (22, 27) | 3 (2, 3) | 28 (25, 31) |
| French Guiana | -0 (-0, 0) | 4 (3, 4) | -0 (-0, -0) | 4 (4, 5) | 0 (0, 0) | 4 (4, 5) | 0 (0, 0) | 4 (4, 5) | 0 (0, 0) | 5 (4, 5) | 0 (0, 0) | 6 (5, 6) |
| Réunion | -0 (-0, 0) | 6 (6, 7) | -0 (-0, -0) | 7 (6, 7) | 0 (0, 0) | 6 (6, 7) | 1 (1, 1) | 6 (5, 6) | 1 (1, 1) | 6 (5, 6) | 1 (1, 1) | 6 (6, 7) |
| Germany | -35 (-74, 4) | 1,478 (1,343, 1,613) | -77 (-130, -24) | 1,651 (1,515, 1,787) | 145 (82, 208) | 1,575 (1,424, 1,727) | 378 (304, 453) | 1,564 (1,395, 1,734) | 491 (410, 571) | 1,659 (1,477, 1,841) | 502 (417, 587) | 1,848 (1,651, 2,044) |
| Greece | -5 (-11, 1) | 300 (273, 328) | -12 (-20, -4) | 326 (299, 353) | 22 (13, 32) | 317 (287, 348) | 59 (47, 70) | 312 (278, 345) | 80 (67, 93) | 338 (300, 375) | 84 (70, 98) | 381 (341, 422) |
| Guatemala | -1 (-2, 0) | 82 (75, 90) | -2 (-4, -1) | 90 (83, 98) | 4 (2, 6) | 82 (74, 90) | 10 (8, 12) | 71 (63, 78) | 13 (11, 15) | 76 (68, 85) | 12 (10, 14) | 89 (80, 98) |
| Iran | -15 (-32, 2) | 818 (744, 893) | -33 (-56, -11) | 900 (826, 974) | 61 (35, 88) | 830 (751, 910) | 159 (127, 190) | 854 (761, 946) | 214 (178, 249) | 937 (834, 1,040) | 222 (184, 260) | 1,063 (950, 1,176) |
| Ireland | -9 (-18, 1) | 372 (338, 406) | -19 (-31, -6) | 419 (385, 454) | 34 (19, 49) | 403 (364, 442) | 89 (71, 106) | 403 (359, 446) | 115 (96, 134) | 405 (360, 449) | 117 (97, 137) | 448 (400, 495) |
| Israel | -2 (-4, 0) | 114 (104, 125) | -4 (-6, -1) | 123 (113, 133) | 7 (4, 10) | 116 (105, 127) | 17 (14, 21) | 114 (102, 126) | 23 (19, 27) | 114 (102, 127) | 24 (20, 28) | 116 (104, 129) |
| Italy | -9 (-19, 1) | 508 (461, 554) | -20 (-33, -6) | 568 (522, 615) | 37 (21, 53) | 541 (489, 593) | 97 (78, 116) | 551 (491, 610) | 130 (108, 151) | 602 (536, 668) | 135 (112, 158) | 681 (609, 754) |
| Japan | -204 (-434, 26) | 9,214 (8,371, 10,057) | -444 (-748, -140) | 10,049 (9,219, 10,878) | 814 (461, 1,167) | 9,495 (8,579, 10,410) | 2,080 (1,671, 2,490) | 9,312 (8,301, 10,322) | 2,722 (2,275, 3,169) | 9,839 (8,757, 10,918) | 2,763 (2,294, 3,232) | 10,741 (9,598, 11,882) |
| Kuwait | -1 (-1, 0) | 39 (36, 43) | -1 (-2, -0) | 43 (39, 47) | 3 (2, 4) | 41 (37, 45) | 7 (6, 8) | 40 (35, 44) | 10 (8, 11) | 40 (35, 44) | 10 (8, 12) | 43 (39, 48) |
| Mexico | -26 (-56, 3) | 2,647 (2,405, 2,888) | -58 (-97, -18) | 2,858 (2,622, 3,093) | 108 (61, 154) | 2,587 (2,338, 2,836) | 276 (222, 331) | 2,400 (2,140, 2,660) | 364 (304, 424) | 2,393 (2,131, 2,655) | 375 (311, 439) | 2,701 (2,414, 2,987) |
| Moldova | -2 (-3, 0) | 71 (65, 78) | -4 (-6, -1) | 79 (72, 85) | 7 (4, 10) | 73 (66, 80) | 18 (14, 21) | 74 (66, 82) | 24 (20, 27) | 81 (72, 89) | 24 (20, 28) | 91 (81, 100) |
| Netherland | -5 (-10, 1) | 205 (186, 224) | -11 (-18, -3) | 230 (211, 249) | 20 (11, 28) | 217 (196, 238) | 51 (41, 61) | 202 (180, 224) | 67 (56, 79) | 216 (192, 240) | 70 (58, 82) | 246 (220, 272) |
| Norway | -1 (-3, 0) | 51 (46, 56) | -3 (-5, -1) | 58 (53, 63) | 6 (3, 8) | 57 (52, 63) | 15 (12, 18) | 57 (51, 64) | 19 (16, 22) | 61 (54, 67) | 20 (17, 24) | 66 (59, 73) |
| Panama | -0 (-1, 0) | 23 (21, 26) | -1 (-1, -0) | 22 (21, 24) | 1 (1, 2) | 22 (20, 24) | 3 (3, 4) | 21 (18, 23) | 4 (3, 5) | 21 (18, 23) | 4 (3, 5) | 23 (20, 25) |
| Paraguay | -1 (-2, 0) | 41 (37, 45) | -2 (-3, -1) | 44 (41, 48) | 4 (2, 6) | 43 (39, 47) | 11 (9, 13) | 42 (38, 47) | 14 (12, 17) | 45 (40, 49) | 15 (13, 18) | 49 (44, 54) |
| Peru | -10 (-20, 1) | 963 (875, 1,051) | -20 (-33, -6) | 1,044 (958, 1,130) | 33 (19, 48) | 984 (889, 1,079) | 83 (67, 100) | 996 (888, 1,104) | 104 (87, 121) | 1,098 (977, 1,218) | 106 (88, 124) | 1,252 (1,119, 1,384) |
| Philippines | -5 (-11, 1) | 428 (389, 467) | -11 (-19, -4) | 442 (406, 479) | 21 (12, 30) | 406 (366, 445) | 54 (43, 65) | 405 (361, 449) | 70 (58, 81) | 440 (392, 489) | 73 (61, 86) | 499 (445, 552) |
| Portugal | -10 (-21, 1) | 571 (519, 623) | -22 (-37, -7) | 639 (586, 692) | 41 (23, 59) | 621 (561, 680) | 107 (86, 129) | 623 (555, 690) | 133 (111, 155) | 671 (597, 744) | 139 (116, 163) | 753 (673, 832) |
| Puerto Rico | -0 (-1, 0) | 21 (19, 23) | -1 (-1, -0) | 21 (19, 23) | 1 (1, 2) | 21 (19, 23) | 3 (2, 4) | 23 (20, 25) | 4 (3, 4) | 26 (23, 28) | 4 (3, 4) | 30 (27, 33) |
| Romania | -11 (-22, 1) | 621 (564, 677) | -23 (-39, -7) | 708 (650, 766) | 43 (25, 62) | 685 (619, 751) | 115 (93, 138) | 681 (607, 754) | 155 (129, 180) | 724 (645, 803) | 163 (135, 190) | 786 (703, 869) |
| South Africa | -121 (-256, 15) | 8,724 (7,929, 9,517) | -262 (-441, -83) | 9,393 (8,621, 10,164) | 481 (272, 689) | 8,703 (7,867, 9,538) | 1,235 (992, 1,478) | 8,310 (7,411, 9,208) | 1,605 (1,341, 1,868) | 8,686 (7,735, 9,635) | 1,635 (1,357, 1,912) | 9,710 (8,681, 10,737) |
| South Korea | -34 (-72, 4) | 1,583 (1,438, 1,727) | -75 (-126, -24) | 1,749 (1,605, 1,893) | 139 (79, 200) | 1,612 (1,457, 1,767) | 364 (292, 435) | 1,540 (1,373, 1,707) | 480 (401, 559) | 1,597 (1,422, 1,772) | 495 (411, 579) | 1,714 (1,532, 1,897) |
| Spain | -21 (-45, 3) | 1,293 (1,175, 1,411) | -47 (-79, -15) | 1,449 (1,330, 1,569) | 88 (50, 126) | 1,399 (1,264, 1,533) | 229 (184, 274) | 1,401 (1,249, 1,553) | 302 (252, 351) | 1,508 (1,342, 1,673) | 311 (258, 364) | 1,702 (1,521, 1,882) |
| Sweden | -6 (-13, 1) | 226 (206, 247) | -13 (-22, -4) | 258 (236, 279) | 24 (14, 35) | 253 (228, 277) | 64 (51, 76) | 254 (227, 282) | 82 (69, 95) | 270 (240, 299) | 84 (70, 98) | 298 (266, 330) |
| Switzerland | -3 (-6, 0) | 129 (118, 141) | -7 (-11, -2) | 147 (135, 159) | 13 (7, 18) | 139 (125, 152) | 33 (26, 39) | 136 (121, 151) | 44 (36, 51) | 144 (128, 160) | 45 (37, 52) | 159 (142, 176) |
| Taiwan | -11 (-23, 1) | 499 (454, 545) | -24 (-41, -8) | 547 (502, 593) | 45 (25, 64) | 511 (462, 561) | 117 (94, 141) | 518 (462, 574) | 153 (128, 178) | 548 (488, 608) | 160 (133, 187) | 625 (559, 692) |
| Thailand | -19 (-41, 2) | 1,964 (1,784, 2,143) | -42 (-71, -13) | 2,080 (1,908, 2,251) | 78 (44, 112) | 1,879 (1,698, 2,060) | 204 (163, 244) | 1,751 (1,561, 1,941) | 269 (225, 313) | 1,834 (1,632, 2,035) | 279 (232, 327) | 2,076 (1,855, 2,297) |
| UK | -46 (-98, 6) | 1,728 (1,569, 1,886) | -102 (-171, -32) | 1,973 (1,810, 2,136) | 187 (106, 269) | 1,921 (1,735, 2,106) | 486 (390, 581) | 1,901 (1,695, 2,108) | 626 (523, 729) | 2,031 (1,808, 2,254) | 641 (532, 750) | 2,288 (2,045, 2,532) |
| Uruguay | -9 (-18, 1) | 298 (270, 325) | -18 (-31, -6) | 345 (317, 374) | 32 (18, 46) | 323 (291, 354) | 82 (66, 98) | 304 (271, 337) | 100 (83, 116) | 310 (276, 344) | 104 (86, 122) | 350 (313, 387) |
| USA | -330 (-701, 42) | 12,353 (11,224, 13,481) | -736 (-1,239, -232) | 13,681 (12,552, 14,809) | 1,379 (781, 1,977) | 12,861 (11,622, 14,099) | 3,583 (2,878, 4,287) | 12,471 (11,118, 13,823) | 4,716 (3,942, 5,490) | 12,975 (11,550, 14,398) | 4,831 (4,011, 5,650) | 13,982 (12,495, 15,467) |
| Vietnam | -3 (-6, 0) | 192 (174, 209) | -6 (-10, -2) | 180 (165, 195) | 10 (6, 15) | 171 (154, 187) | 26 (21, 31) | 192 (171, 213) | 33 (28, 38) | 222 (198, 247) | 33 (28, 39) | 262 (234, 290) |
| Pooled | -1,168 (-2,484, 147) | 57,420 (52,172, 62,661) | -2,568 (-4,327, -810) | 63,014 (57,816, 68,206) | 4,762 (2,697, 6,826) | 59,179 (53,477, 64,872) | 12,296 (9,878, 14,713) | 57,589 (51,342, 63,828) | 16,092 (13,449, 18,732) | 60,605 (53,951, 67,248) | 16,476 (13,678, 19,271) | 67,035 (59,909, 74,149) |

## Table S21. Annual average attributable deaths of both inter-day TV and intra-day TV for cardiovascular mortality

| Country/region | TV 0–1 | | TV 0–2 | | TV 0–3 | | TV 0–4 | | TV 0–5 | | TV 0–6 | |
| --- | --- | --- | --- | --- | --- | --- | --- | --- | --- | --- | --- | --- |
|  | Inter-day | Intra-day | Inter-day | Intra-day | Inter-day | Intra-day | Inter-day | Intra-day | Inter-day | Intra-day | Inter-day | Intra-day |
| Brazil | 33 (16, 49) | 589 (511, 667) | 49 (23, 74) | 732 (644, 820) | 118 (88, 149) | 781 (682, 880) | 187 (152, 222) | 804 (693, 914) | 246 (208, 283) | 831 (712, 949) | 257 (216, 297) | 921 (790, 1,052) |
| Canada | 49 (24, 74) | 473 (410, 535) | 74 (35, 112) | 590 (519, 661) | 177 (131, 222) | 636 (555, 716) | 279 (227, 332) | 648 (559, 736) | 366 (310, 421) | 666 (571, 761) | 380 (320, 439) | 725 (622, 828) |
| China | 69 (35, 104) | 899 (780, 1,018) | 103 (50, 157) | 1,116 (982, 1,251) | 248 (184, 311) | 1,160 (1,012, 1,307) | 389 (316, 462) | 1,125 (971, 1,279) | 515 (437, 593) | 1,142 (979, 1,305) | 542 (457, 627) | 1,243 (1,066, 1,420) |
| Colombia | 7 (4, 11) | 145 (126, 164) | 10 (5, 15) | 160 (141, 179) | 22 (16, 28) | 169 (148, 191) | 34 (27, 40) | 173 (149, 196) | 41 (35, 47) | 182 (156, 208) | 42 (35, 48) | 204 (175, 233) |
| Costa Rica | 0 (0, 0) | 5 (4, 6) | 0 (0, 0) | 6 (5, 6) | 1 (0, 1) | 6 (5, 6) | 1 (1, 1) | 6 (5, 7) | 1 (1, 1) | 6 (6, 7) | 1 (1, 1) | 7 (6, 8) |
| Czech Republic | 15 (8, 23) | 130 (113, 147) | 23 (11, 35) | 170 (149, 190) | 57 (42, 71) | 183 (160, 207) | 90 (73, 107) | 187 (161, 212) | 118 (100, 136) | 192 (164, 219) | 125 (106, 145) | 209 (179, 238) |
| Ecuador | 3 (1, 4) | 62 (54, 71) | 4 (2, 6) | 60 (53, 68) | 9 (7, 11) | 54 (47, 61) | 14 (12, 17) | 57 (49, 65) | 17 (14, 19) | 63 (54, 72) | 15 (13, 17) | 73 (63, 84) |
| Estonia | 3 (2, 5) | 38 (33, 43) | 5 (2, 8) | 48 (43, 54) | 12 (9, 16) | 52 (45, 59) | 19 (16, 23) | 53 (46, 60) | 25 (21, 29) | 53 (45, 60) | 27 (22, 31) | 56 (48, 64) |
| Finland | 2 (1, 3) | 23 (20, 26) | 3 (2, 5) | 29 (25, 32) | 8 (6, 10) | 31 (27, 35) | 13 (11, 16) | 32 (27, 36) | 18 (15, 20) | 33 (28, 38) | 18 (15, 21) | 36 (31, 42) |
| Greece | 8 (4, 12) | 136 (118, 154) | 12 (6, 19) | 166 (146, 186) | 31 (23, 38) | 180 (157, 203) | 49 (40, 59) | 183 (158, 208) | 67 (57, 77) | 191 (164, 218) | 72 (61, 83) | 211 (181, 241) |
| Iran | 21 (10, 32) | 334 (290, 379) | 32 (15, 49) | 412 (363, 462) | 76 (56, 96) | 423 (369, 477) | 121 (98, 143) | 449 (388, 511) | 162 (137, 186) | 476 (408, 544) | 172 (145, 199) | 528 (453, 603) |
| Ireland | 9 (4, 13) | 115 (100, 130) | 13 (6, 20) | 145 (127, 162) | 31 (23, 39) | 155 (136, 175) | 50 (41, 60) | 160 (138, 182) | 65 (55, 75) | 155 (133, 178) | 67 (57, 78) | 168 (144, 192) |
| Japan | 229 (114, 344) | 3,061 (2,655, 3,466) | 339 (163, 515) | 3,741 (3,290, 4,193) | 805 (598, 1,013) | 3,944 (3,442, 4,446) | 1,262 (1,026, 1,499) | 3,994 (3,445, 4,541) | 1,647 (1,397, 1,897) | 4,064 (3,484, 4,644) | 1,704 (1,437, 1,972) | 4,335 (3,717, 4,952) |
| Kuwait | 1 (1, 2) | 18 (16, 20) | 2 (1, 2) | 22 (20, 25) | 4 (3, 5) | 24 (21, 27) | 6 (5, 7) | 24 (20, 27) | 8 (7, 9) | 23 (20, 26) | 9 (7, 10) | 24 (21, 28) |
| Mexico | 22 (11, 34) | 651 (565, 737) | 34 (16, 51) | 790 (695, 885) | 81 (60, 102) | 799 (697, 900) | 128 (104, 152) | 765 (661, 870) | 169 (143, 194) | 734 (630, 839) | 177 (149, 205) | 812 (696, 927) |
| Norway | 2 (1, 3) | 19 (16, 21) | 3 (1, 4) | 24 (21, 27) | 6 (5, 8) | 26 (23, 29) | 10 (8, 12) | 27 (23, 31) | 13 (11, 15) | 27 (24, 31) | 14 (12, 16) | 29 (25, 34) |
| Panama | 0 (0, 1) | 8 (7, 9) | 1 (0, 1) | 8 (7, 9) | 1 (1, 1) | 9 (8, 10) | 2 (2, 2) | 9 (7, 10) | 2 (2, 3) | 8 (7, 10) | 3 (2, 3) | 9 (8, 10) |
| Paraguay | 1 (0, 1) | 13 (11, 14) | 1 (1, 2) | 15 (14, 17) | 4 (3, 5) | 17 (14, 19) | 6 (5, 7) | 17 (14, 19) | 8 (7, 9) | 17 (15, 20) | 9 (7, 10) | 18 (16, 21) |
| Philippines | 6 (3, 9) | 149 (129, 169) | 9 (4, 14) | 173 (152, 194) | 22 (16, 27) | 177 (154, 199) | 34 (28, 41) | 182 (157, 207) | 44 (37, 51) | 191 (164, 218) | 47 (40, 55) | 211 (181, 241) |
| Portugal | 12 (6, 18) | 204 (177, 231) | 18 (9, 27) | 256 (225, 287) | 44 (33, 55) | 277 (242, 312) | 70 (57, 84) | 287 (248, 326) | 87 (74, 100) | 298 (256, 340) | 93 (78, 107) | 327 (281, 373) |
| South Africa | 59 (30, 89) | 1,294 (1,123, 1,465) | 88 (42, 133) | 1,563 (1,375, 1,751) | 209 (155, 262) | 1,616 (1,411, 1,820) | 329 (268, 391) | 1,595 (1,377, 1,812) | 428 (363, 493) | 1,610 (1,381, 1,838) | 445 (375, 514) | 1,758 (1,509, 2,007) |
| South Korea | 25 (13, 38) | 349 (302, 395) | 38 (18, 58) | 431 (379, 483) | 92 (68, 116) | 444 (387, 500) | 147 (120, 175) | 438 (378, 498) | 194 (165, 224) | 438 (376, 501) | 204 (172, 236) | 460 (394, 525) |
| Spain | 24 (12, 35) | 427 (371, 484) | 36 (17, 54) | 536 (472, 601) | 87 (64, 109) | 578 (504, 651) | 139 (113, 164) | 597 (515, 679) | 182 (154, 210) | 620 (531, 708) | 191 (161, 221) | 683 (586, 780) |
| Sweden | 8 (4, 13) | 94 (82, 107) | 13 (6, 19) | 120 (106, 135) | 30 (22, 38) | 132 (115, 148) | 49 (40, 58) | 137 (118, 155) | 63 (53, 72) | 140 (120, 160) | 65 (55, 76) | 151 (130, 173) |
| Switzerland | 4 (2, 5) | 46 (40, 52) | 6 (3, 8) | 59 (52, 66) | 13 (10, 17) | 62 (54, 70) | 22 (18, 26) | 63 (54, 71) | 29 (24, 33) | 64 (55, 73) | 30 (25, 35) | 69 (59, 79) |
| Taiwan | 8 (4, 12) | 108 (94, 122) | 12 (6, 19) | 133 (117, 149) | 30 (22, 37) | 138 (121, 156) | 48 (39, 57) | 145 (125, 165) | 62 (52, 71) | 148 (127, 169) | 66 (56, 76) | 165 (141, 188) |
| Thailand | 12 (6, 17) | 348 (302, 394) | 17 (8, 26) | 414 (364, 464) | 41 (30, 51) | 418 (365, 471) | 66 (53, 78) | 403 (347, 458) | 86 (73, 99) | 407 (349, 465) | 91 (77, 106) | 451 (386, 515) |
| UK | 55 (27, 83) | 606 (526, 687) | 82 (40, 125) | 776 (682, 870) | 197 (146, 248) | 843 (736, 951) | 313 (254, 372) | 862 (744, 981) | 403 (342, 464) | 888 (761, 1,015) | 421 (355, 487) | 978 (839, 1,117) |
| USA | 386 (192, 579) | 4,202 (3,645, 4,758) | 585 (281, 888) | 5,217 (4,587, 5,845) | 1,421 (1,055, 1,786) | 5,474 (4,777, 6,170) | 2,264 (1,840, 2,688) | 5,487 (4,734, 6,238) | 2,972 (2,521, 3,422) | 5,508 (4,722, 6,293) | 3,101 (2,615, 3,587) | 5,795 (4,969, 6,618) |
| Vietnam | 2 (1, 3) | 42 (37, 48) | 3 (1, 4) | 45 (39, 50) | 7 (5, 8) | 48 (42, 54) | 10 (8, 12) | 55 (47, 63) | 13 (11, 15) | 61 (52, 70) | 14 (12, 16) | 70 (60, 80) |
| Pooled | 1,077 (537, 1,617) | 14,589 (12,657, 16,518) | 1,613 (775, 2,450) | 17,959 (15,791, 20,122) | 3,883 (2,884, 4,882) | 18,854 (16,453, 21,250) | 6,153 (5,000, 7,305) | 18,960 (16,360, 21,555) | 8,048 (6,826, 9,269) | 19,239 (16,493, 21,978) | 8,401 (7,082, 9,718) | 20,727 (17,776, 23,671) |

## Table S22. Annual average attributable deaths of both inter-day TV and intra-day TV for respiratory mortality

| Country/region | TV 0–1 | | TV 0–2 | | TV 0–3 | | TV 0–4 | | TV 0–5 | | TV 0–6 | |
| --- | --- | --- | --- | --- | --- | --- | --- | --- | --- | --- | --- | --- |
|  | Inter-day | Intra-day | Inter-day | Intra-day | Inter-day | Intra-day | Inter-day | Intra-day | Inter-day | Intra-day | Inter-day | Intra-day |
| Brazil | 5 (-7, 18) | 109 (63, 155) | -1 (-16, 15) | 152 (101, 202) | 35 (15, 54) | 155 (98, 212) | 66 (44, 88) | 192 (123, 260) | 72 (47, 97) | 240 (161, 318) | 69 (44, 95) | 291 (205, 377) |
| Canada | 5 (-7, 18) | 57 (33, 81) | -1 (-17, 15) | 80 (53, 106) | 35 (15, 54) | 83 (52, 113) | 66 (44, 88) | 101 (65, 137) | 72 (47, 97) | 126 (84, 167) | 69 (44, 95) | 150 (106, 194) |
| China | 9 (-12, 30) | 128 (74, 182) | -1 (-28, 25) | 180 (120, 240) | 58 (26, 91) | 181 (114, 248) | 111 (74, 148) | 212 (136, 287) | 122 (80, 165) | 258 (173, 343) | 119 (75, 164) | 307 (216, 397) |
| Colombia | 1 (-2, 4) | 27 (15, 38) | -0 (-3, 3) | 33 (22, 44) | 6 (3, 10) | 33 (21, 45) | 12 (8, 16) | 41 (26, 55) | 12 (8, 16) | 52 (35, 69) | 11 (7, 15) | 64 (45, 82) |
| Costa Rica | 0 (-0, 0) | 1 (0, 1) | -0 (-0, 0) | 1 (1, 1) | 0 (0, 0) | 1 (1, 1) | 0 (0, 0) | 1 (1, 1) | 0 (0, 0) | 1 (1, 2) | 0 (0, 0) | 2 (1, 2) |
| Czech Republic | 1 (-1, 2) | 7 (4, 10) | -0 (-2, 2) | 10 (7, 14) | 5 (2, 8) | 11 (7, 14) | 9 (6, 13) | 13 (8, 18) | 10 (7, 14) | 16 (11, 21) | 10 (6, 14) | 19 (14, 25) |
| Ecuador | 0 (-1, 2) | 13 (7, 18) | -0 (-1, 1) | 14 (9, 18) | 3 (1, 5) | 12 (8, 17) | 5 (4, 7) | 15 (10, 21) | 5 (3, 7) | 20 (14, 27) | 4 (3, 6) | 26 (18, 33) |
| Estonia | 0 (-0, 0) | 1 (1, 2) | -0 (-0, 0) | 2 (1, 2) | 1 (0, 1) | 2 (1, 2) | 1 (1, 2) | 2 (1, 3) | 1 (1, 2) | 3 (2, 4) | 1 (1, 2) | 3 (2, 4) |
| Finland | 0 (-0, 1) | 2 (1, 3) | -0 (-1, 0) | 3 (2, 4) | 1 (1, 2) | 3 (2, 4) | 2 (1, 3) | 3 (2, 5) | 2 (2, 3) | 4 (3, 6) | 2 (1, 3) | 5 (4, 7) |
| France | 2 (-3, 8) | 33 (19, 48) | -0 (-7, 6) | 47 (32, 63) | 15 (6, 23) | 48 (30, 66) | 28 (19, 37) | 59 (38, 80) | 30 (20, 40) | 73 (49, 97) | 29 (18, 40) | 88 (62, 114) |
| Greece | 1 (-1, 3) | 14 (8, 20) | -0 (-2, 2) | 19 (13, 26) | 5 (2, 8) | 20 (13, 28) | 10 (7, 13) | 25 (16, 33) | 11 (7, 15) | 31 (21, 41) | 11 (7, 15) | 38 (26, 49) |
| Iran | 2 (-2, 5) | 27 (16, 38) | -0 (-5, 4) | 37 (25, 50) | 10 (4, 16) | 37 (23, 50) | 19 (13, 26) | 47 (30, 63) | 22 (14, 29) | 60 (40, 79) | 21 (13, 29) | 73 (51, 94) |
| Ireland | 2 (-3, 6) | 27 (16, 38) | -0 (-6, 5) | 38 (25, 51) | 12 (5, 19) | 39 (25, 54) | 23 (15, 31) | 49 (31, 66) | 25 (16, 34) | 57 (38, 76) | 24 (15, 33) | 68 (48, 87) |
| Japan | 36 (-50, 122) | 550 (317, 782) | -5 (-111, 101) | 752 (502, 1,002) | 231 (102, 360) | 762 (481, 1,042) | 435 (291, 579) | 926 (595, 1,256) | 474 (310, 637) | 1,139 (764, 1,514) | 451 (284, 618) | 1,331 (938, 1,722) |
| Kuwait | 0 (-0, 0) | 1 (1, 2) | -0 (-0, 0) | 2 (1, 3) | 0 (0, 1) | 2 (1, 3) | 1 (1, 1) | 2 (2, 3) | 1 (1, 1) | 3 (2, 4) | 1 (1, 1) | 3 (2, 4) |
| Mexico | 4 (-5, 12) | 122 (71, 174) | -1 (-11, 10) | 165 (110, 220) | 23 (10, 36) | 160 (101, 219) | 44 (29, 59) | 185 (119, 251) | 48 (32, 65) | 215 (144, 286) | 47 (29, 64) | 260 (184, 337) |
| Norway | 0 (-0, 1) | 2 (1, 3) | -0 (-1, 1) | 3 (2, 5) | 1 (1, 2) | 4 (2, 5) | 3 (2, 3) | 4 (3, 6) | 3 (2, 4) | 6 (4, 7) | 3 (2, 4) | 6 (5, 8) |
| Panama | 0 (-0, 0) | 1 (1, 1) | -0 (-0, 0) | 1 (1, 1) | 0 (0, 0) | 1 (1, 2) | 0 (0, 1) | 1 (1, 2) | 0 (0, 1) | 2 (1, 2) | 0 (0, 1) | 2 (1, 2) |
| Paraguay | 0 (-0, 0) | 2 (1, 3) | -0 (-0, 0) | 2 (2, 3) | 1 (0, 1) | 3 (2, 3) | 2 (1, 2) | 3 (2, 4) | 2 (1, 3) | 4 (3, 5) | 2 (1, 3) | 4 (3, 6) |
| Philippines | 1 (-1, 4) | 29 (17, 41) | -0 (-3, 3) | 38 (25, 50) | 7 (3, 11) | 37 (23, 51) | 13 (9, 17) | 46 (30, 62) | 14 (9, 19) | 58 (39, 77) | 14 (9, 19) | 70 (50, 91) |
| Portugal | 1 (-2, 4) | 25 (14, 36) | -0 (-4, 4) | 35 (23, 47) | 9 (4, 14) | 37 (23, 50) | 17 (11, 22) | 46 (29, 62) | 17 (11, 23) | 57 (38, 76) | 17 (11, 23) | 69 (49, 89) |
| South Africa | 21 (-28, 70) | 530 (306, 753) | -3 (-64, 58) | 715 (478, 952) | 133 (58, 207) | 709 (448, 969) | 251 (168, 334) | 841 (541, 1,139) | 272 (178, 365) | 1,021 (685, 1,354) | 260 (164, 356) | 1,224 (864, 1,582) |
| South Korea | 4 (-5, 12) | 55 (32, 79) | -1 (-11, 10) | 77 (51, 102) | 23 (10, 36) | 76 (48, 104) | 45 (30, 59) | 90 (58, 122) | 49 (32, 66) | 108 (73, 144) | 47 (30, 65) | 125 (88, 161) |
| Spain | 3 (-5, 11) | 70 (40, 100) | -1 (-11, 10) | 99 (66, 131) | 23 (10, 36) | 102 (65, 140) | 44 (29, 58) | 127 (82, 172) | 48 (31, 64) | 159 (107, 212) | 46 (29, 63) | 193 (136, 249) |
| Sweden | 1 (-1, 2) | 8 (5, 12) | -0 (-2, 2) | 12 (8, 15) | 4 (2, 7) | 12 (8, 17) | 8 (6, 11) | 15 (10, 21) | 9 (6, 12) | 19 (13, 25) | 8 (5, 12) | 22 (16, 29) |
| Switzerland | 0 (-0, 1) | 4 (2, 6) | -0 (-1, 1) | 6 (4, 7) | 2 (1, 3) | 6 (4, 8) | 4 (2, 5) | 7 (4, 9) | 4 (3, 5) | 9 (6, 11) | 4 (2, 5) | 10 (7, 13) |
| Taiwan | 1 (-2, 5) | 23 (13, 33) | -0 (-5, 4) | 32 (21, 42) | 10 (4, 15) | 32 (20, 44) | 19 (13, 25) | 40 (26, 54) | 21 (14, 28) | 50 (33, 66) | 20 (13, 28) | 60 (43, 78) |
| Thailand | 3 (-5, 11) | 116 (67, 165) | -0 (-10, 9) | 155 (103, 206) | 21 (9, 33) | 150 (94, 205) | 41 (27, 55) | 174 (111, 235) | 45 (29, 61) | 211 (142, 281) | 44 (28, 60) | 256 (180, 331) |
| UK | 10 (-13, 33) | 117 (68, 167) | -1 (-30, 28) | 168 (112, 224) | 63 (28, 99) | 176 (111, 240) | 122 (81, 162) | 216 (138, 293) | 130 (85, 175) | 268 (180, 356) | 126 (79, 172) | 324 (228, 419) |
| USA | 43 (-58, 143) | 542 (313, 771) | -6 (-135, 122) | 753 (502, 1,003) | 286 (126, 445) | 758 (478, 1,037) | 549 (367, 730) | 912 (586, 1,237) | 600 (393, 807) | 1,106 (741, 1,469) | 578 (364, 791) | 1,278 (901, 1,653) |
| Vietnam | 0 (-0, 1) | 7 (4, 11) | -0 (-1, 1) | 9 (6, 12) | 2 (1, 3) | 9 (6, 12) | 3 (2, 5) | 12 (8, 17) | 4 (2, 5) | 17 (11, 22) | 4 (2, 5) | 21 (15, 28) |
| Pooled | 157 (-215, 528) | 2,652 (1,529, 3,772) | -23 (-489, 442) | 3,640 (2,429, 4,847) | 1,025 (451, 1,598) | 3,658 (2,309, 5,003) | 1,954 (1,307, 2,599) | 4,407 (2,831, 5,977) | 2,126 (1,390, 2,861) | 5,392 (3,615, 7,162) | 2,045 (1,289, 2,799) | 6,392 (4,508, 8,268) |


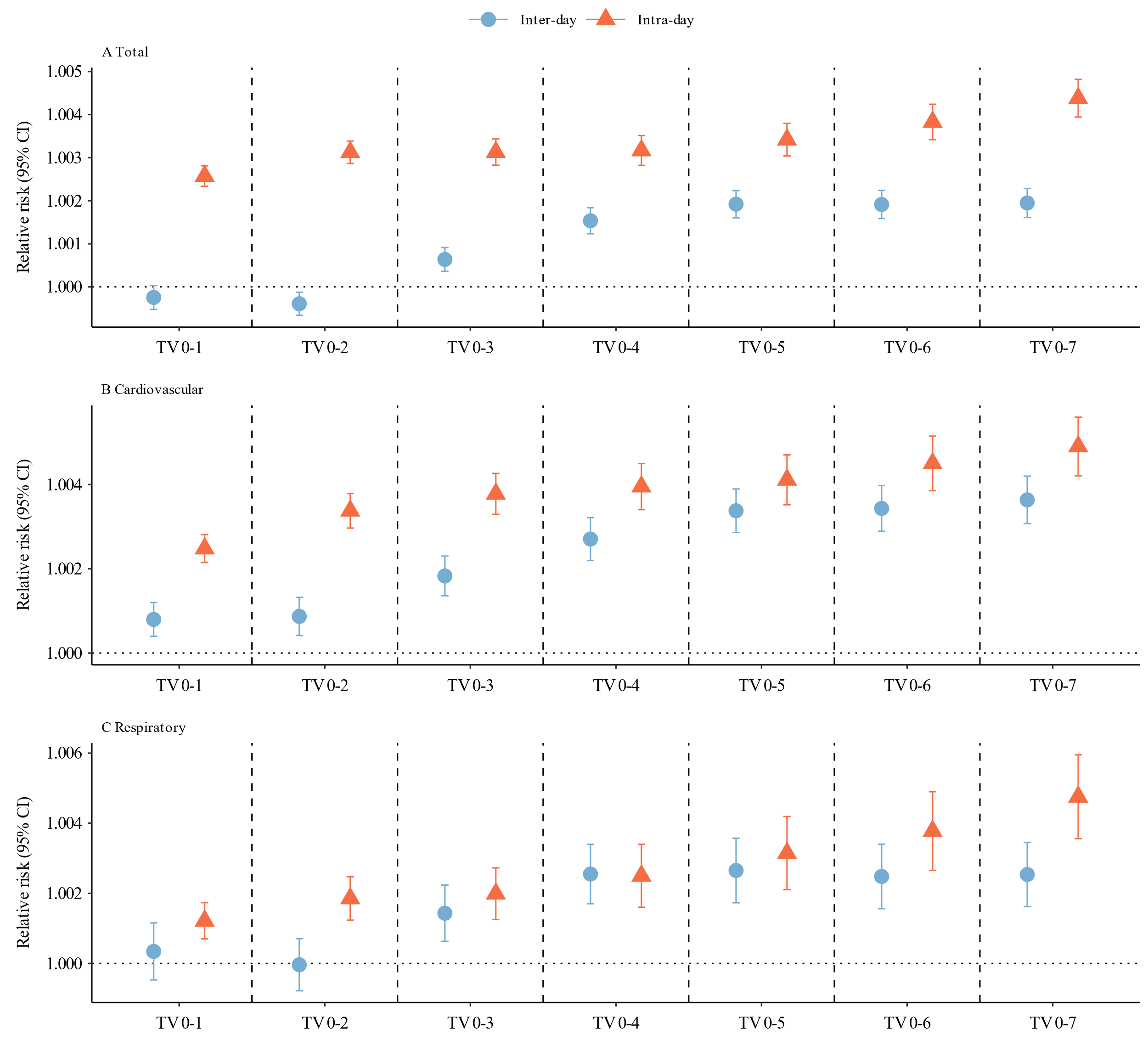


## Figure S1. Sensitivity analyses of comparing effects of inter-day and intra-day TV per 1℃ increase in TV.


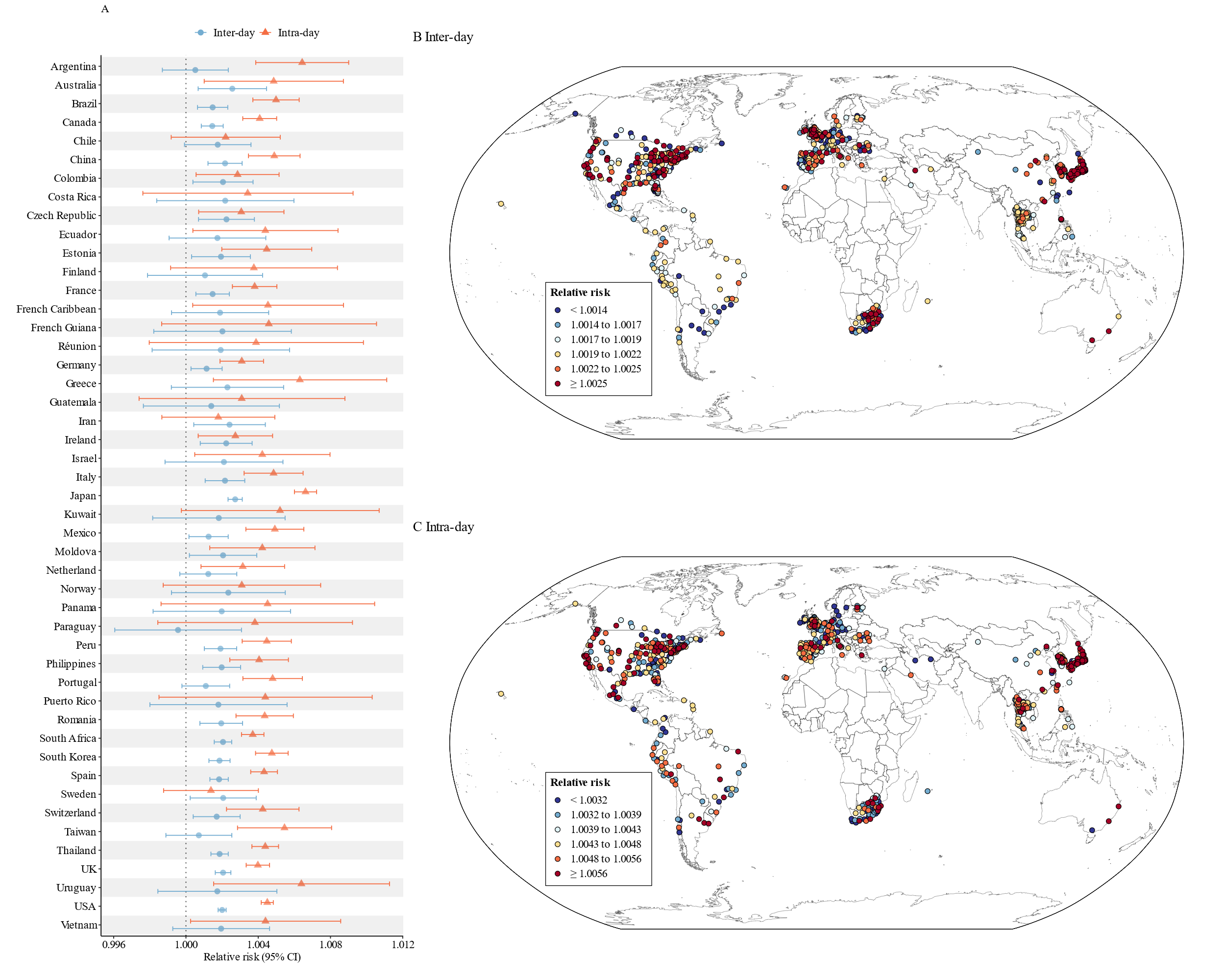


## Figure S2. Sensitivity analyses of comparing effects of inter-day and intra-day TV per 1℃ increase of TV in all countries/regions (A) and locations (B and C).


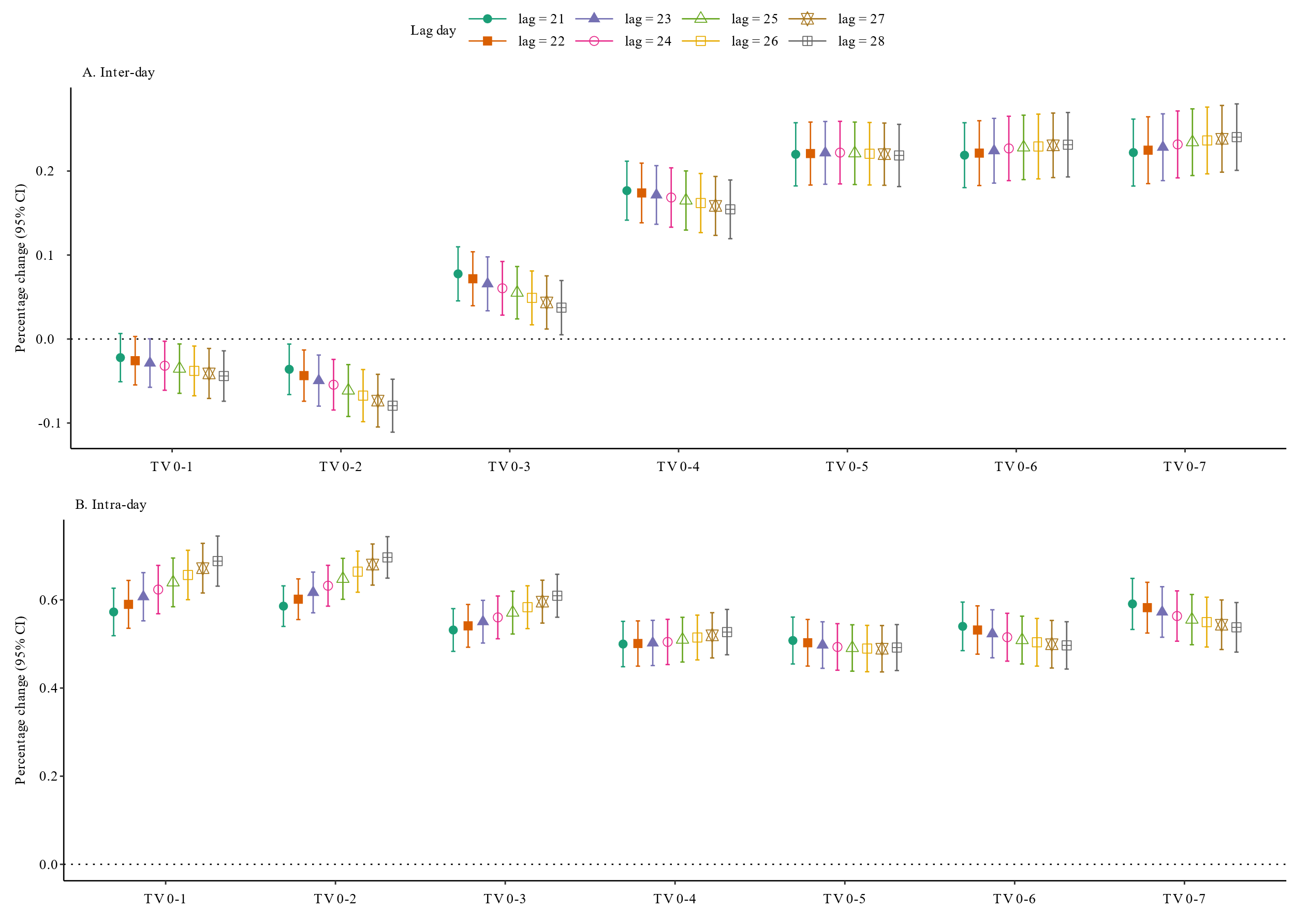


## Figure S3. Sensitivity analyses to change lag days for daily mean temperatures (21 to 28 days)


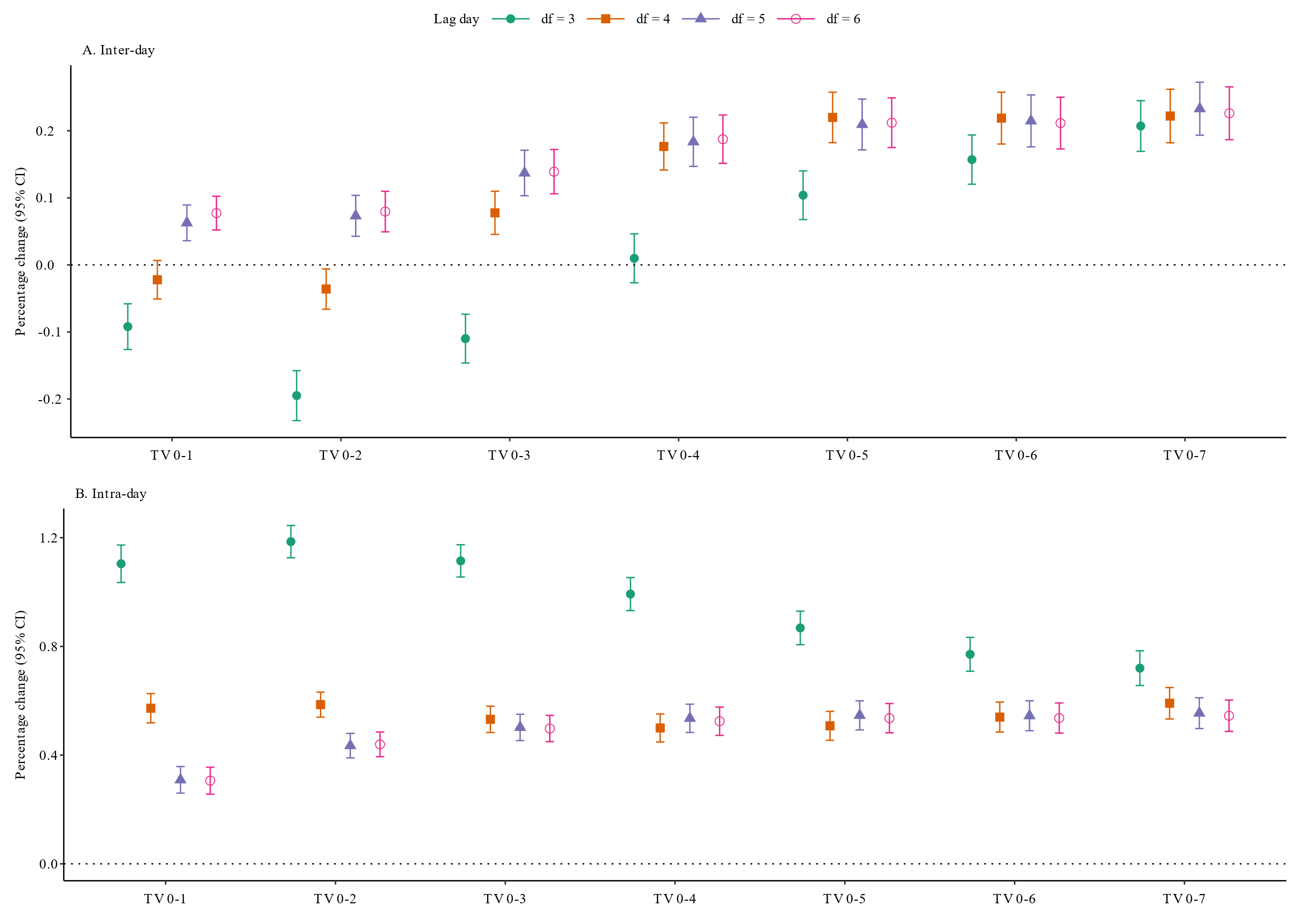


## Figure S4. Sensitivity analyses to change df for daily mean temperatures (3–6 df)


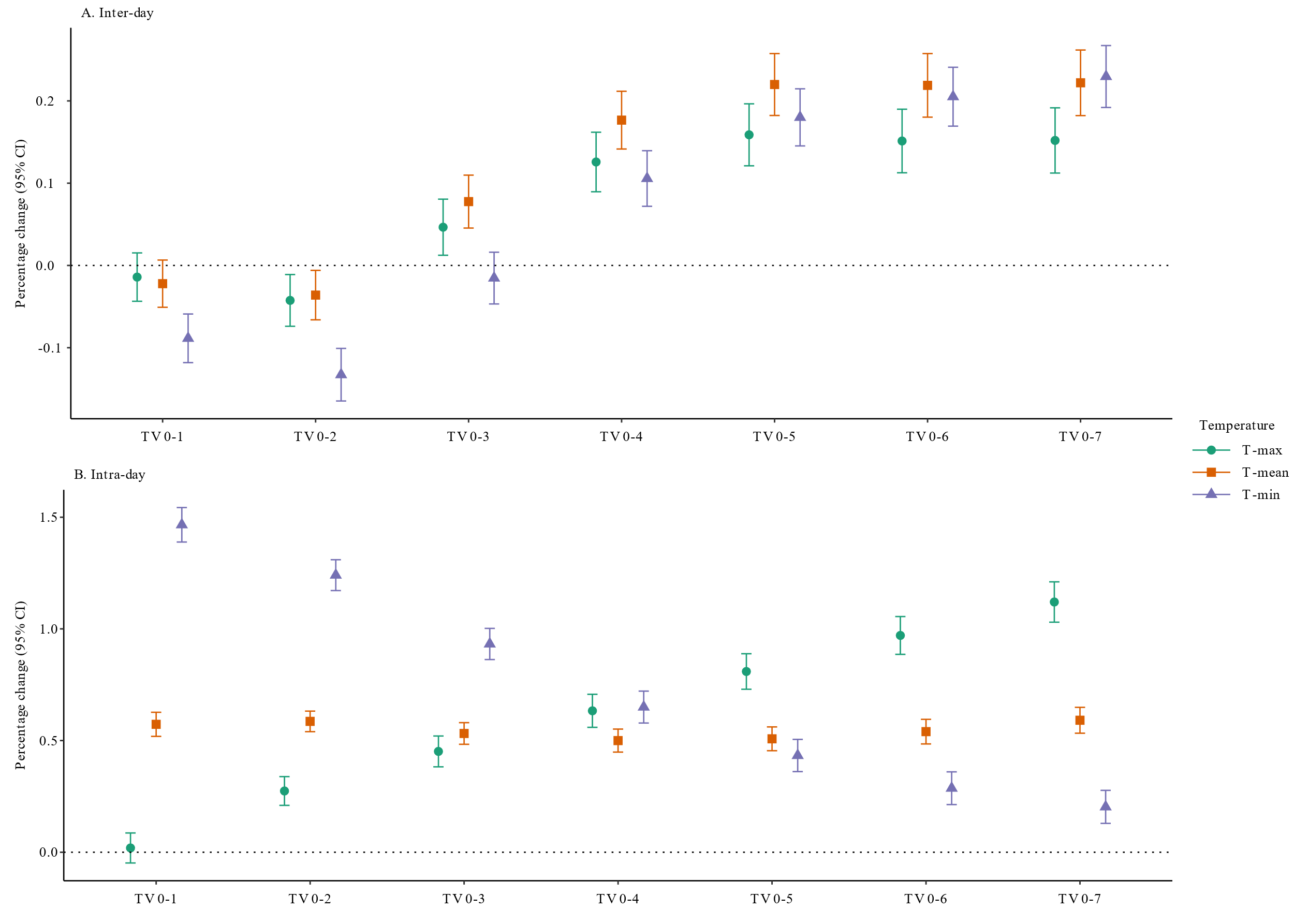


## Figure S5. Sensitivity analyses to change daily mean temperature to daily maximum or minimum temperature. T-max = daily maximum temperature, T-mean = daily mean temperature, T-min = daily minimum temperature.


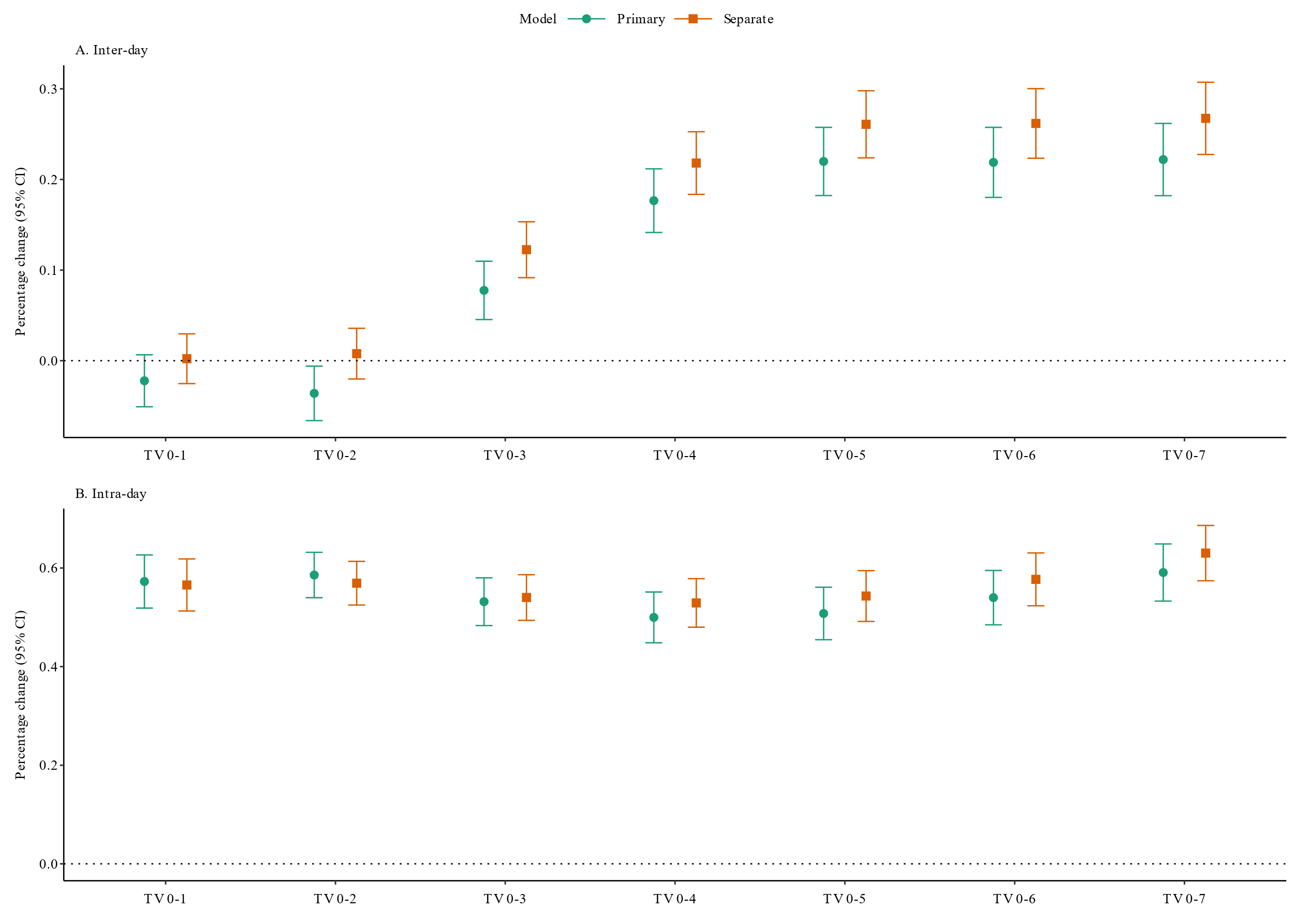


## Figure S6. Sensitivity analyses to include only one of TV indicators (inter-day TV and intra-day TV) in the models.


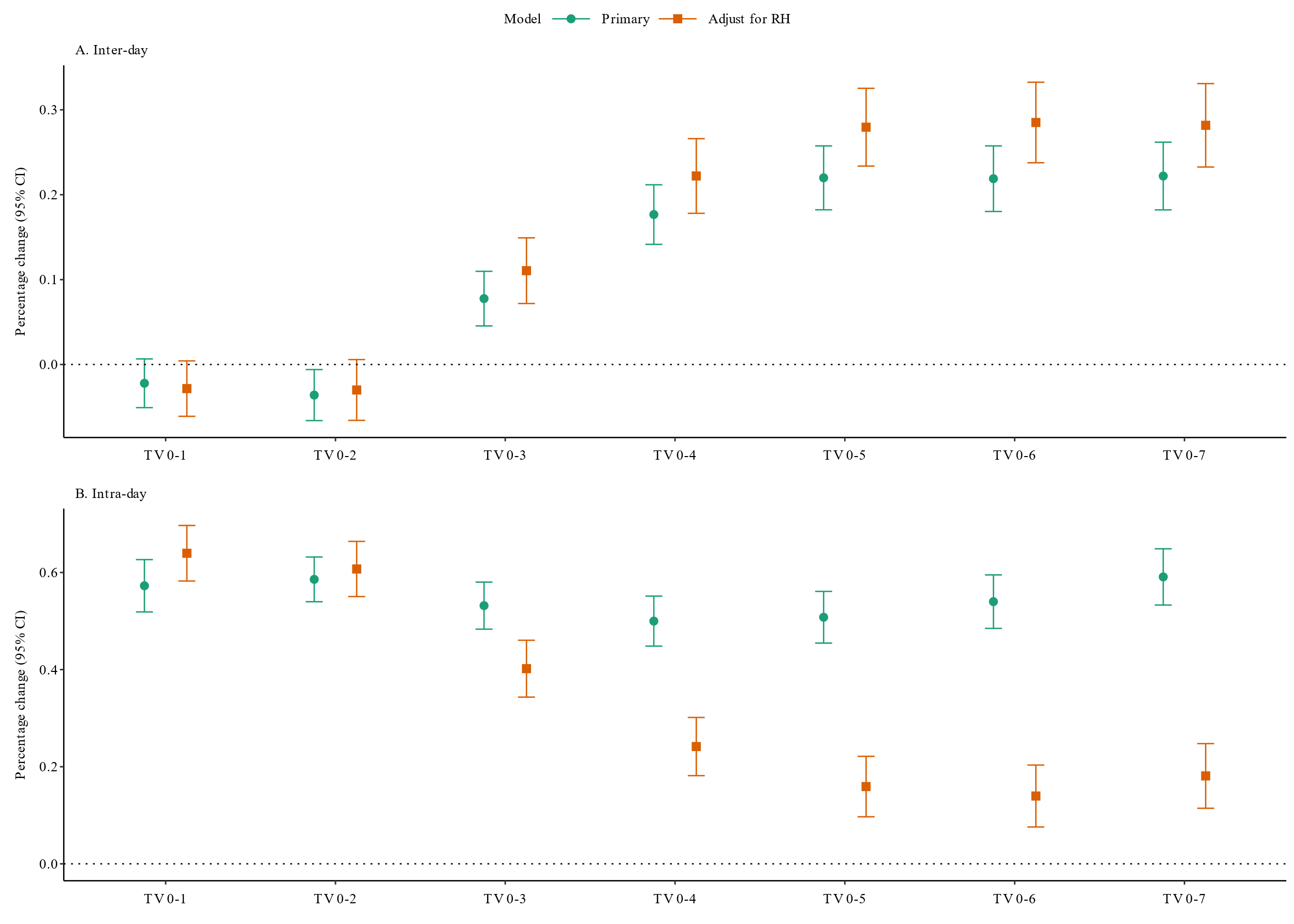


## Figure S7. Sensitivity analyses to include relative humidity in the model.


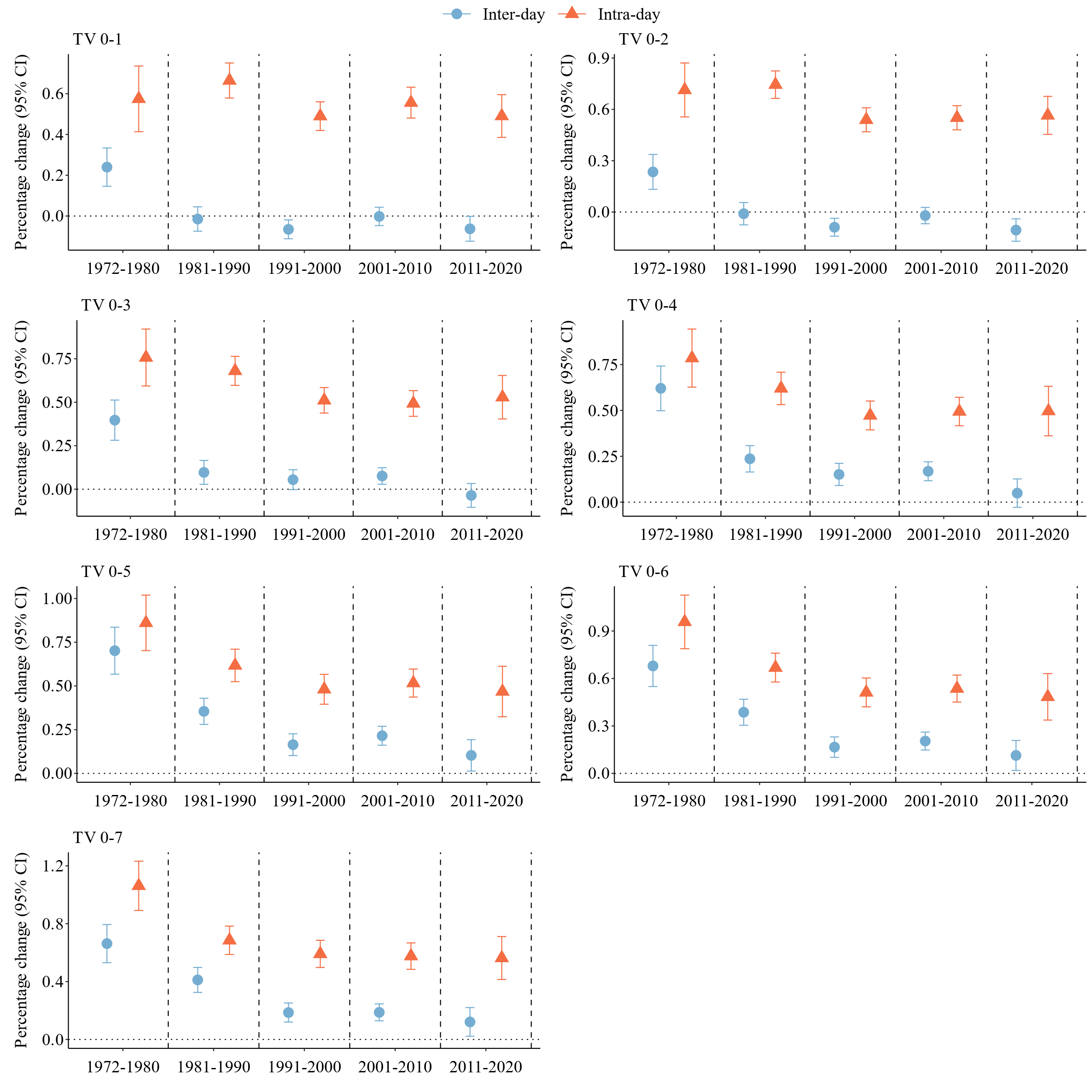


## Figure S8. Overall percentage change (%) of mortality risk over one interquartile range of inter-day and intra-day TV (TV0–1 to TV0–7) by study period (1972–1980, 1981–1990, 1991–2000, 2001–2010, 2011–2020).
